# Single-Atom Tuning of Structural and Optoelectronic Properties in Halogenated Anthracene-Based Covalent Organic Frameworks

**DOI:** 10.1021/acsomega.6c01720

**Published:** 2026-05-19

**Authors:** Klaudija Paliušytė, Laura Fuchs, Zehua Xu, Kuangjie Liu, Kornel Roztocki, Shuo Sun, Hendrik Zipse, Achim Hartschuh, Frank Ortmann, Jenny Schneider

**Affiliations:** † Department of Chemistry and Center for NanoScience (CeNS), 2331 University of Munich (LMU), Munich 81377, Germany; ‡ Department of Chemistry, TUM School of Natural Sciences and Atomistic Modeling Center, Munich Data Science Institute, 9184 Technische Universität München, Garching bei München 85748, Germany; § Faculty of Chemistry, 49562 Adam Mickiewicz University, Uniwersytetu Poznańskiego 8, Poznań 61-614, Poland

## Abstract

Strategies for tuning structural and (opto-)­electronic properties are fundamental to the rational design of functional materials. Here, we present a molecular-design approach that enables precise modulation of the optoelectronic properties of covalent organic frameworks (COFs) through single-atom halogen substitution on π-extended anthracene linkers. Using a Wurster-type tetratopic amine (W–NH_2_) and a series of anthracene-based dialdehydes bearing H, Cl, Br, or I at the 2-position, a family of imine-linked COFs, W-A-X (X = H, Cl, Br, I), was synthesized. The halogen substituent strongly influences framework formation, with brominated COFs forming substantially larger crystalline domains than the unsubstituted analogue. UV–vis absorption and photoluminescence measurements reveal a systematic redshift across the series (H < Cl < Br < I), demonstrating that a single-atom modification tunes the optical response. In addition, the significantly longer excited-state lifetime of W-A-Cl COF highlights the strengthened donor–acceptor interactions induced by the more electron-withdrawing chlorine substituent, further confirming the decisive role of single-atom functionalization in controlling excited-state dynamics. Time-dependent density functional theory calculations on both isolated fragments and extended COF models attribute the observed trends in optical response to halogen-induced changes in the COF band structure and provide a mechanistic understanding of how a single-atom substitution influences the optoelectronic properties of the extended π-framework. Overall, this study establishes single-atom halogen substitution as a powerful and modular tool for tailoring the structural and optical properties of anthracene-based COFs.

## Graphical abstract

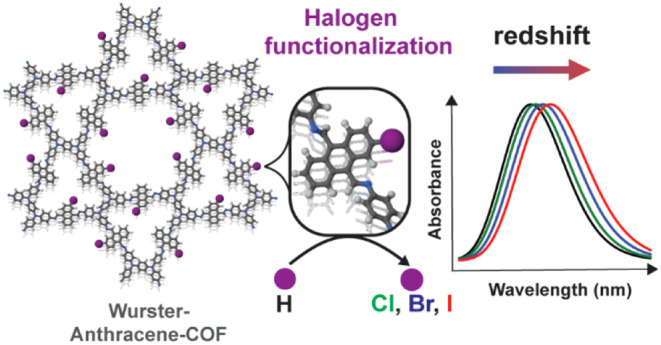

## 1 Introduction

Covalent organic frameworks (COFs) are porous crystalline polymers constructed from a diverse set of molecular organic building blocks, linked through covalent bonds in a periodic arrangement.^1^ This structural regularity enables precise control over their properties, resulting in a rich spectrum of functionalities. The ability to design COFs with high structural precision allows for a direct connection between their architecture and (opto-)­electronic properties, establishing well-defined structure–property relationships that are critical for the development of novel functional materials. These frameworks can be designed with numerous organic molecules capable of engaging in extensive chemical interactions, further broadening their utility.^2^ The choice of molecular building units is therefore pivotal in defining structural, electronic, and chemical characteristics of COFs, directly influencing their applicability in fields such as photocatalysis,^3^ photovoltaics,^4^ sensing,^5^ and gas storage.^6^

Anthracene, an aromatic polycyclic compound composed of three linearly fused benzene rings, is a particularly compelling molecular building block for COF synthesis. In its native form, anthracene absorbs in the UV region and emits in the blue region of the visible spectrum, which limits direct applicability in visible-light-driven technologies. To fully exploit its potential in solar energy conversion systems, a redshift of the optical response is required.^7,8^ This can be achieved by integrating anthracene units into extended π-conjugated frameworks such as COFs, or through peripheral chemical functionalization that preserves the integrity of the aromatic core.^9,10^

Owing to the favorable optoelectronic properties of anthracene derivatives such as strong photoluminescence,^11^ efficient intramolecular charge transfer,^11^ and mechanochromic^12^ behavior, anthracene offers significant promise as a versatile structural motif for advanced COF design. The anthracene-based COFs reported to date have been explored for catalysis,^13–15^ fluorescence quenching,^16,17^ sensing,^18,19^ supercapacitors,^20^ and optoelectronic applications.^21,22^ However, the large size and inherent planarity of anthracene, together with the positioning of its functional groups, can hinder the formation of highly crystalline COFs. Consequently, combining anthracene with more flexible comonomers may enable the synthesis of well-ordered, crystalline frameworks.

In this context, *N*,*N*,*N*′,*N*′-tetraphenyl-1,4-phenylen (Wurster-type) building blocks offer a particularly attractive option for COF synthesis due to their structural flexibility, which stems from free rotation around single bonds.^23,24^ This adaptability allows them to conform to more rigid components such as anthracene, facilitating the formation of well-ordered, crystalline frameworks. Notably, the additional aromatic rings in Wurster-type building blocks enhance π-conjugation, enabling fine-tuning of the (opto-)­electronic properties and expanding the design space for modular and functional COF architectures.^23,25^ Specifically, the combination of electron-donating Wurster-type units with electron-accepting anthracene moieties can promote the formation of donor–acceptor COFs, a design strategy known to enhance charge carrier separation and improve the efficiency of light-induced processes.^26^

One effective strategy for modulating the properties of COFs is the integration of functional groups or atoms, among which halogen functionalization stands out as particularly impactful. The incorporation of halogens into the COF backbone can enhance charge separation and transfer efficiency owing to their electron-withdrawing characteristics^27,28^ or tune the COF’s electrostatic potential.^29^ Furthermore, halogen-framework interactions have been reported to induce changes in both the electronic structure and the molecular geometry of the framework, making halogenation a powerful strategy for tuning properties of organic porous semiconductors.^30^ However, the majority of halogenated COFs described to date are derived from small benzene-based building blocks bearing halogen substituents,^31–37^ whereas examples incorporating larger, π-extended halogenated units are still limited. To the best of our knowledge, halogenated anthracene units have not previously been incorporated into COFs, although even a single halogen atom is expected to significantly alter orbital energies and the resulting electronic structure of the extended framework.^38^

Here, we introduce a concept for the atom-precise tuning of structural and optoelectronic properties in COFs based on single-atom halogen substitution of π-extended anthracene linkers. By combining a Wurster-type tetratopic amine node (W–NH_2_) with a series of anthracene-based dialdehydes functionalized at the 2-position with H, Cl, Br, or I, we construct a family of imine-linked anthracene COFs, denoted W-A-X (X = H, Cl, Br, I), that are isostructural yet electronically distinct. This systematic variation of a single substituent enables us to isolate and investigate the influence of minimal atomic-level modifications on framework formation, crystallinity, morphology, porosity, and optoelectronic response. Through a combination of optical spectroscopy, time-resolved photophysical measurements, and density functional theory (DFT) calculations on functionalized molecular building blocks, extended framework fragments, and periodic COF models, we obtain a fundamental understanding of how halogen identity modulates orbital energies and the band structure in the extended π-framework. Altogether, this work establishes single-atom halogen substitution as a powerful and modular strategy for engineering the structure–property relationships of anthracene-based COFs, providing a rational design principle for tailoring porous organic semiconductors for optoelectronic and photocatalytic applications.

## 2 Methods

### 2.1 Chemicals

All materials were purchased from Aldrich, Fluka, Acros, Activate Scientific, or TCI Europe in the common purities purum, puriss, or reagent grade. Materials were used as received without additional purification and handled under air unless noted otherwise. All used solvents were anhydrous and purged with inert gas.

### 2.2 Syntheses of Halogenated Building Blocks and COFs

Detailed synthesis procedures and schematic illustrations are provided in Supporting Information.

### 2.3 Powder X-ray Diffraction (PXRD) Measurements

PXRD measurements were performed on a Bruker D8 Discover diffractometer using Ni-filtered Cu Kα radiation and a position sensitive LynxEye detector in Bragg–Brentano geometry.

### 2.4 Nitrogen Sorption Measurements

Nitrogen sorption isotherms were recorded on a Quantachrome Autosorb 1 at 77 K within a pressure range from P/P_0_ = 0.001 to 0.98. Prior to the measurement of the sorption isotherms, the samples were heated for 24 h at 393 K under turbo-pumped vacuum. For the evaluation of the surface area, the Brunauer–Emmett–Teller (BET) model was applied between 0.05 and 0.28 P/P_0_, considering an experimentally determined standard deviation of 8%. Pore size distributions were calculated using the NLDFT equilibrium model with a carbon kernel for slit/cylindrical pores.^39^

The **structure models of the COFs** were constructed using the Accelrys Materials Studio software package. For W-A-H, P-3 symmetry and for W-A-Cl, W-A-Br and W-A-I COFs P-1 symmetry was applied. The structure models were optimized using the Forcite module with the Dreiding force-field. Further refinements using the Pawley method were carried out as implemented in the Reflex module of the Materials Studio software. Thompson-Cox-Hastings peak profiles were used, and peak asymmetry was corrected using the Berar-Baldinozzi method.

### 2.5 Liquid State ^1^H and ^13^C Nuclear Magnetic Resonance (NMR) Analysis

Liquid state NMR spectra were recorded on Bruker AV 400 and AV 400 TR spectrometers. Proton chemical shifts are expressed in parts per million (δ scale) and are calibrated using residual nondeuterated solvent peaks as internal reference (e.g., CDCl_3_: 7.26 ppm in ^1^H NMR and 77.0 ppm in ^13^C NMR).

### 2.6 Solid State ^13^C NMR Analysis

The solid state ^13^C cross-polarization magic angle spinning (CP/MAS) spectra were obtained on a Bruker Avance III-500 solid state NMR spectrometer with a 4 mm double resonance MAS probe and at a MAS rate of 10.0 kHz with a contact time of 2–5 ms and a pulse delay of 4 s.

### 2.7 Fourier-Transform Infrared (FT-IR) Spectra

FT-IR measurements were performed with a Bruker Vertex 70 FTIR instrument by focusing light of a globar (silicon carbide) as MIR light source through a KBr beam splitter with integrated gold mirrors and an ATR sample stage with a Ge crystal. The spectra were recorded by an N_2_-cooled MCT detector with a resolution of 2 cm^–1^ and averaged over 1000 scans.

### 2.8 Thermogravimetric Analysis (TGA)

TGA measurements were performed on a Netzsch Jupiter STA 449 C instrument equipped with a Netzsch TASC 414/4 controller. The samples were heated from room temperature to 900 °C under a synthetic air flow (25 mL min^–1^) at a heating rate of 10 K min^–1^.

### 2.9 Scanning Electron Microscopy (SEM)

SEM images were recorded with an FEI Helios NanoLab G3 UC scanning electron microscope equipped with a field emission gun operated at 3 kV. Prior to the measurements, the samples were sputtered with carbon.

### 2.10 High-Resolution Transmission Electron Microscopy (HRTEM)

HRTEM images were recorded with an FEI Titan Themis 60–300 equipped with a field emission gun operated at 300 kV.

### 2.11 Ultraviolet-Vis (UV–Vis) Absorption Spectroscopy

The UV–vis spectra were recorded on a PerkinElmer Lambda 1050 spectrometer equipped with a 150 mm integrating sphere with InGaAs detector. Diffuse reflectance spectra were collected with a Praying Mantis (Harrick) accessory and were referenced to barium sulfate powder as white standard. The specular reflection of the sample surface was removed from the signal using apertures that allow only light scattered at angles >20° to pass.

### 2.12 Steady-State Photoluminescence (PL) and Time-Correlated Single-Photon Counting (TCSPC)

A home-built confocal laser scanning microscope (CLSM) setup was used for characterizing the photoluminescence of powder of W-A-X COFs (X = H, Cl, Br, I) and their linkers A-X-CHO (X = H, Cl, Br, I). The samples were measured in the epi-direction using an air objective (0.85 NA, Fluor 40, NIKON). A beamsplitter (MELLES GRIOT 03BTL005) and a 490 nm long-pass filter were utilized to separate the laser from the photoluminescence (PL) light. Excitation was provided by a subpicosecond laser (iChrome TOPTICA) operating at 476 nm with repetition rate of 40 MHz. The detection system was divided into two components. The first part featured an avalanche photodiode (APD, type: MPD PDM, with a detector size of 50 × 50 μm), which was used in combination with time-correlated single-photon counting (TCSPC) electronics (BECKER & HICKL) to measure time-resolved PL transients. The second part comprised a spectrometer (ANDOR SHAMROCK SRi303) connected to a CCD camera (ANDOR NEWTON DU920) for capturing spectra. The data were recorded using a customized LABVIEW (National Instruments) program that integrated the manufacturers’ software with our specific measurement requirements. Further data processing and analysis, including extracting PL spectra and TCSPC transients, were performed using MATLAB (MATHWORKS).

### 2.13 Density Functional Theory

Theoretical calculations of structural and electronic properties of the four COFs and their fragments were performed with Vienna Ab initio Simulation Package (VASP),^40–43^ using the projector-augmented wave (PAW) method^44,45^ in combination with the Perdew–Burke–Ernzerhof (PBE) exchange correlation functional^46^ and periodic boundary conditions. For the gas-phase calculations for all building blocks and their extended fragments, a vacuum of at least 5 Å in each direction was applied. For the COF structures, we employed the Becke–Johnson damping variant of DFT-D3 ^47^ to correct the van der Waals (vdW) dispersion. We used 1 × 1 × 1 (gas-phase calculations) and 1 × 1 × 5 (periodic calculations) grids for k-point sampling. Geometry optimization of the atomic positions and the lattice parameters of the molecular structures were optimized in an alternating fashion with multiple steps, where an energy convergence value of 10^–6^ eV and a kinetic energy cutoff of 400 eV (for atom relaxation) and 520 eV (for lattice relaxation, only for 2D COFs) were used. The electronic band structure was computed along the high symmetry paths in the Brillouin zone of the primitive hexagonal unit cell. Each segment along the high symmetry paths Γ – M – K – Γ – A was sampled by 10 points. To account for the opening of the band gap and to estimate the band gap at the Hybrid-DFT level (Heyd–Scuseria–Ernzerhof (HSE06) functional),^48^ we employed a scissors shift^49^ along the high symmetry path.

### 2.14 Time-Dependent Density Functional Theory

Theoretical calculations of optical properties of the gas-phase molecules were performed with the Gaussian 16 software package.^50^ We employed the HSE06 exchange-correlation functional and the Def2TZVP^51,52^ as basis set.

## 3 Results and Discussion

### 3.1 Synthesis and Characterization

Novel anthracene-based linkers, functionalized with different halogens at the 2-position, were synthesized following a general synthetic route outlined in Scheme S1.^53^ The synthesis involves a Diels–Alder reaction between halogenated anthracene precursors (2-X-anthracene (A-X); X = Cl, Br, I) and vinylene carbonate to form a cyclic carbonate intermediate. This intermediate is subsequently converted into the corresponding diol, which is then oxidized to yield the target compound: 2-halogen-9,10-anthracenedialdehyde (A-X-CHO; X = Cl, Br, I). Details on the synthesis are provided in the SI (Figures S1–S16).

The freshly synthesized halogenated A-X-CHO linkers, along with the commercially available nonhalogenated analog, were employed to construct four crystalline COFs (W-A-X, where X = H, Cl, Br, I) via a Schiff-base condensation reaction with the electron-rich *N*,*N*,*N*′,*N*′-tetrakis­(4-aminophenyl)-1,4-phenylenediamine (W-NH_2_) building block (see Figure 1a, Figures S17–S20). Powder X-ray diffraction (PXRD) analysis reveals well-ordered structures of all synthesized COFs (see Figure 1b–d). All four COFs exhibit prominent diffraction peaks corresponding to the (100), (110), and (210) lattice planes at similar positions. Additionally, (200) and (310) peaks were observed for W-A-H, W-A-Cl, and W-A-Br. Intense and sharp diffraction peaks for W-A-H, W-A-Cl, and W-A-Br COFs confirm their high crystallinity, while the W-A-I COF features lower crystallinity.

**Figure 1:**
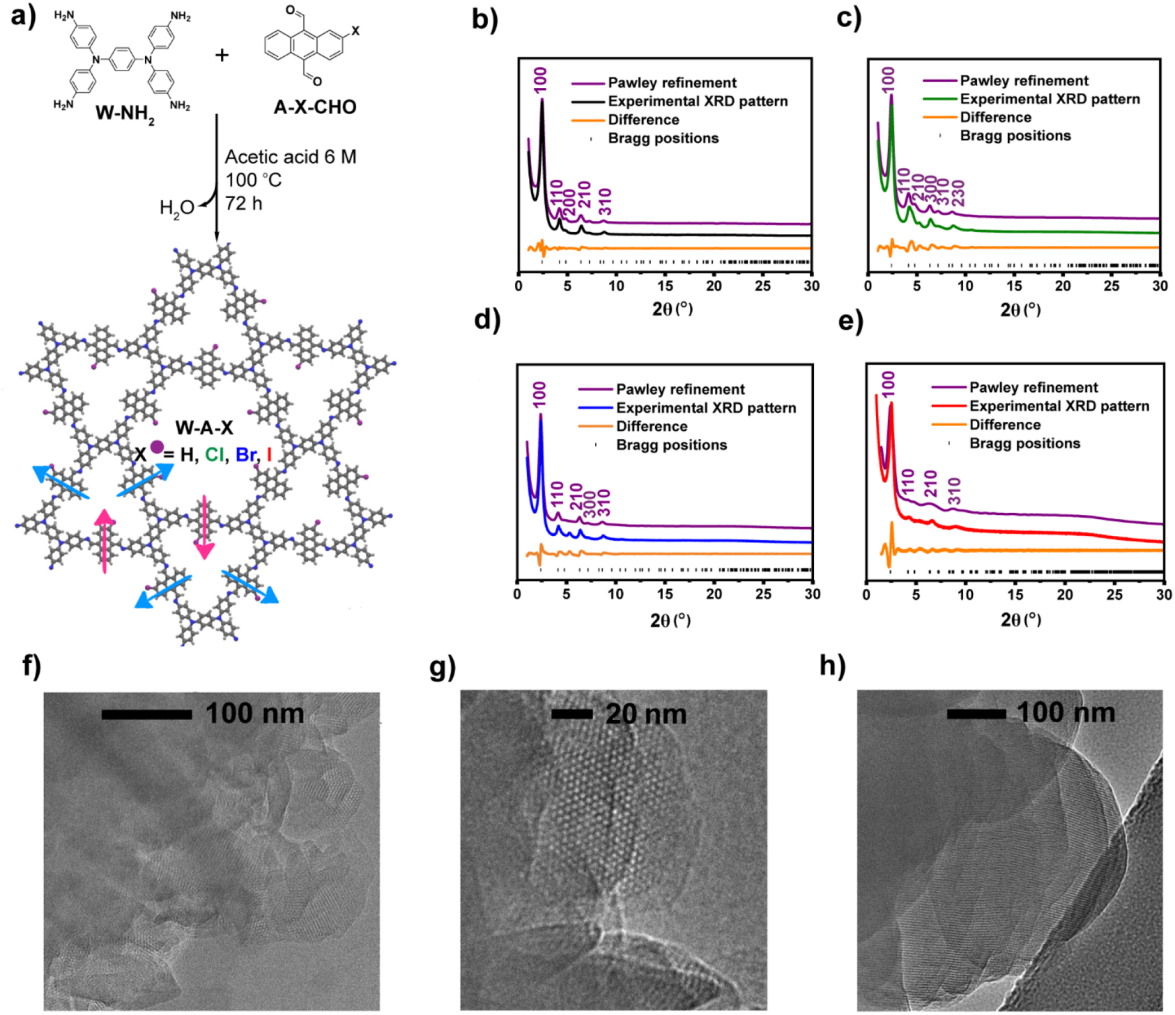
(a) Schematic representation of Schiff-base reaction to obtain W-A-X COFs (X = H, Cl, Br, I). Arrows indicate the orientation of the halogen atoms of the energetically most stable configuration with two halogens facing the smaller trigonal pore (pink) and the remaining four halogens directed toward the larger hexagonal pore shown in blue. Simulated and experimental PXRD patterns of (b) W-A-H, (c) W-A-Cl, (d) W-A-Br, and (e) W-A-I. HRTEM images of (f) W-A-H, (g) W-A-Cl, and (h) W-A-Br.

Building on the PXRD characterization, we further investigated the structure of all four W-A-X COFs using DFT simulations. For better comparison of the four models, we assumed hexagonal lattice symmetry with a kagome lattice structure in all cases. Each unit cell comprises six monohalogenated anthracenes and three Wurster units per layer, arranged in an eclipsed vertical stacking geometry. Due to the asymmetric structure of the functionalized anthracene building blocks, various orientations and combinations of the halogenated units relative to the smaller and larger pores are possible. Multiple halogen atom arrangements were explored (see Figure S21), yielding the most energetically favorable configuration (shown in Figure 1a), which also exhibited the best agreement with the experimental PXRD data. In this structural model, two halogen atoms per unit cell are oriented toward the smaller trigonal pore (highlighted with pink arrows), while the remaining four halogen atoms are facing the larger hexagonal pore (blue arrows). For comparison, the nonhalogenated analog (W-A-H), in which halogen atoms were replaced by hydrogen, was used as a reference structure in the simulations.

Based on the structural DFT models and the PXRD patterns (Figure 1b–e), we performed Pawley refinement for all four COFs, adopting P-3 symmetry for W-A-H and P-1 for W-A-X (X = Cl, Br, I). The crystallographic information for the W-A-X COFs is provided in CIF format in the Supporting Information. The refined unit cell parameters are as follows: W-A-H (a = b = 4.236 nm, c = 0.411 nm), W-A-Cl (a = b = 4.265 nm, c = 0.408 nm), and W-A-Br (a = b = 4.263 nm, c = 0.409 nm). The experimental PXRD patterns for these COFs show excellent agreement between the simulated and experimental diffraction peaks with R_p_ values of 4.66%, 5.09%, and 5.64%, respectively. All observed diffraction peaks were successfully indexed to specific lattice planes, confirming the high crystallinity of the synthesized frameworks. The W-A-I (a = b = 4.263 nm, c = 0.408 nm) COF exhibits slightly lower crystallinity compared to the other three COFs. Nevertheless, its experimental PXRD pattern shows a good agreement with the simulated pattern of the dual-pore hexagonal structural model, with a refinement factor of R_p_ = 3.34%.

We note that minor additional reflections were observed in the PXRD patterns of the W-A-Cl and W-A-Br COFs. Specifically, diffraction peaks at 5.25° and 5.30°, respectively, could not be assigned to the simulated dual-pore hexagonal model, suggesting the presence of a minor phase impurity in these frameworks, possibly caused by the asymmetry of the halogenated anthracene linker.

To gain deeper insight into the structural properties of the synthesized COFs, High-ResolutionTransmission Electron Microscopy (HRTEM) analysis was performed for W-A-H, -Cl, -Br COFs as representatives of the series. HRTEM images reveal lattice fringes consistent with well-ordered stacked layers, see Figure 1f–h. The measured lattice fringe spacing of 3.70, 3.68, and 3.66 nm for W-A-H, W-A-Cl, and W-A-Br COFs, respectively (Figures S22–S24), agree well with the π–π stacking distances derived from PXRD modeling, which are 3.67 nm for W-A-H and W-A-Cl, and 3.69 nm for W-A-Br. Figure S25 further shows a minor additional phase in Br-COF, consistent with the extra signals observed in the PXRD pattern.

Furthermore, HRTEM images (Figure 1f–h and Figures S22–S24) displays crystalline domain sizes of 50–100 nm for W-A-H COF, up to 50 nm for W-A-Cl, and as large as 200–400 nm for W-A-Br. As a key structural parameter of COFs, crystallite size strongly influences their optoelectronic properties, with larger domains known to enhance charge transport.^54,55^ The difference in crystalline sizes can be rationalized by nucleation and growth dynamics governed by reaction conditions and linker reactivity: slower nucleation favors larger domains, while faster nucleation leads to smaller crystallites.^56,57^ Notably, W-A-Br forms significantly larger domains than W-A-H under identical reaction conditions, highlighting the influence of halogen substitution in the anthracene linker on COF nucleation.

Halogenation introduces polarization into the linker, creating distinct electrostatic potential (ESP) regions that can affect monomer interactions. This effect can modulate monomer–monomer interactions during nucleation. ESP calculations (Figure S29) show that heavier halogens (Br, I) exhibit a positive σ-hole, enabling directional interactions with electron-rich amino groups.^58–60^ These interactions can stabilize early assemblies, slow nucleation, and promote larger domain growth.^61,62^ Consistent with increasing polarizability (I > Br > Cl), σ-hole strength enhances such interactions.^63^ This likely contributes to the large domains observed for W-A-Br (200–400 nm).^64,65^ In contrast, overly strong interactions in W-A-I may disrupt framework ordering, resulting in reduced crystallinity.^66–69^

The morphologies of the COFs were evaluated via scanning electron microscopy (SEM). SEM images demonstrate that despite the similar crystal structure, morphologies of all four COFs differ (Figure S30). The pristine W-A-H COF consists of small platelets, W-A-Cl COF contains a mixture of platelets and spherical particles, W-A-Br COF is composed mainly of spherical particles and W-A-I COF exhibits rods. The morphology of COFs can be significantly influenced by solvent polarity and electrostatic repulsion, the latter of which may arise from the presence of electronegative halogen atoms.^70,71^ These factors affect layer spacing and solvation, possibly leading to different morphologies.

Nitrogen sorption isotherms were recorded to analyze the porosity of all four COFs (Figure S31). A gradual reduction in surface area is observed with increasing atomic radius of the halogen r (r­(H) < r­(Cl) < r­(Br) < r­(I)): W-A-H 548 m^2^ g^–1^, W-A-Cl 490 m^2^ g^–1^, W-A-Br 187 m^2^ g^–1^ and W-A-I 170 m^2^ g^–1^. These values are considerably lower than the theoretical network-accessible surface areas per gram calculated from perfect crystal structures using PoreBlazer:^72^ W-A-H, 897 m^2^ g^–1^; W-A-Cl, 806 m^2^ g^–1^; W-A-Br, 674 m^2^ g^–1^; and W-A-I, 653 m^2^ g^–1^ (Table S1). Nevertheless, the experimental pore volumes for W-A-H (0.35 cm^3^ g^–1^) and W-A-Cl (0.33 cm^3^ g^–1^) are in good agreement with the theoretical values of 0.364 cm^3^ g^–1^ and 0.317 cm^3^ g^–1^, respectively, indicating good crystallinity and stability during activation prior to the N_2_ adsorption experiment. By contrast, W-A-Br and W-A-I COFs show both substantially lower experimental pore volumes (0.18 cm^3^ g^–1^ and 0.13 cm^3^ g^–1^, respectively) and BET surface area than their theoretical counterparts. For the iodine derivative, this is attributed to reduced crystallinity compared with the other three materials (Figure 1e). Furthermore, the activation process (vacuum drying) of COFs may cause partial pore collapse. Due to steric hindrance, this effect may be more pronounced in materials functionalized with larger atoms, such as Br and I, compared to H and Cl,^32,73^ leading to a further reduction in the overall BET surface area and accessible pore volume.

Using nonlocal density functional theory (NLDFT)^39^ for slit and cylindrical pores for evaluation of the isotherms, average pore sizes were calculated to be 1.0 and 1.7 nm for W-A-H, 1.1 and 2.0 nm for W-A-Cl, 1.1 and 2.0 nm for W-A-Br, and 0.9 and 1.5 nm for W-A-I, respectively. Additionally, porosity parameters were simulated using Zeo++ software^74^ (Table S1 and Figure S32), providing theoretical pore sizes and pore volumes. The results indicated pore sizes of 0.7 and 2.0 nm for W-A-H, 0.7 and 1.9 nm for W-A-Cl, 0.7 and 1.9 nm for W-A-Br, and 0.7 and 1.8 nm for W-A-I. The deviations between the experimental and theoretical pore sizes can be attributed to the idealized nature of the theoretical models, which assume a perfectly ordered and defect-free COF structure. In reality, structural imperfections, defects, distortions, and variations in the distribution of halogen substituents can occur, leading to differences in the experimentally observed porosity parameters. Additionally, the same Zeo++ software was applied to calculate average unit cell densities, yielding 0.67 g cm^–3^ for W-A-H, 0.74 g cm^–3^ for W-A-Cl, 0.81 g cm^–3^ for W-A-Br, and 0.90 g cm^–3^ for W-A-I. The increase in unit cell density for halogenated COFs compared to the nonhalogenated W-A-H COF is attributed to the incorporation of halogen atoms, which increase the overall framework density. A comparison of theoretical and experimental porosity parameters is presented in Table S1.

Fourier Transform Infrared Spectroscopy (FTIR) analysis was conducted to confirm the formation of imine bonds following the condensation reaction of the monomers (Figure S33). All four COFs show an FTIR-band at 1609 cm^–1^ which is assigned to the newly formed imine (C=N) bonds.^75,76^ We note that the stretching vibrations of the carbon–halogen bonds are hidden in the fingerprint region (<600–840 cm^–1^ C–Cl, < 700 cm^–1^ C–Br, < 600 cm^–1^ C–I),^77^ therefore these vibration bands are not presented in FTIR spectra.

To further characterize the chemical structure of the four COFs, ^13^C cross-polarization magic angle spinning (CPMAS) analysis was performed (Figure S34). All COFs exhibited a distinct peak at 157 ppm, confirming imine bond formation. An additional peak at 93 ppm in the ^13^C spectrum of the W-A-I COF indicates the presence of a C–I bond. In contrast, the ^13^C NMR signals corresponding to carbons bonded to chlorine (123.14 ppm) and bromine (122.87 ppm) in the W-A-Cl and W-A-Br COFs, respectively, were less distinct due to overlap with other carbon resonances in the 109–140 ppm region of the spectrum.

A good thermal stability of the COFs was confirmed by thermogravimetric analysis (TGA), Figure S35, with decomposition temperatures occurring at 403, 419, 404 and 378 °C for W-A-H, W-A-Cl, W-A-Br and W-A-I COFs, respectively.

### 3.2 Optical Properties

The optical properties of the COFs were examined using UV-vis diffuse reflectance absorption spectroscopy and are presented as the Kubelka–Munk function (F­(R)) for solid materials.^78^ The optical band-gap energies were determined using Tauc plots (direct transition model), yielding 1.70 eV for W-A-H COF, 1.66 eV for W-A-Cl, 1.62 eV for W-A-Br, and 1.61 eV for W-A-I (Figure 2a, inset). The observed decrease in the optical band-gap energy correlates with the increasing size of the halogen atom.^28,31,38,79^ In perovskites, this optical change has been attributed to variations in orbital participation, however, a corresponding analysis of halogen effects in COFs has not yet been reported.^80,81^ Additionally, the optical properties of the W-A-H COF were compared to the previously reported^23^ Wurster-terephthalaldehyde (W-TA) COF, which is structurally similar to the anthracene moiety but lacks the additional fused benzene rings (Figure S36). UV–vis spectra revealed that the W-A-H COF exhibits a redshift of 97 nm relative to the W-TA COF, along with a significantly reduced optical gap energy (1.70 eV vs 1.89 eV). The shift toward longer wavelengths and the concomitant reduction in band gap can be attributed to the augmented conjugation provided by the trifused benzene rings present in anthracene.^13,82^

**Figure 2:**
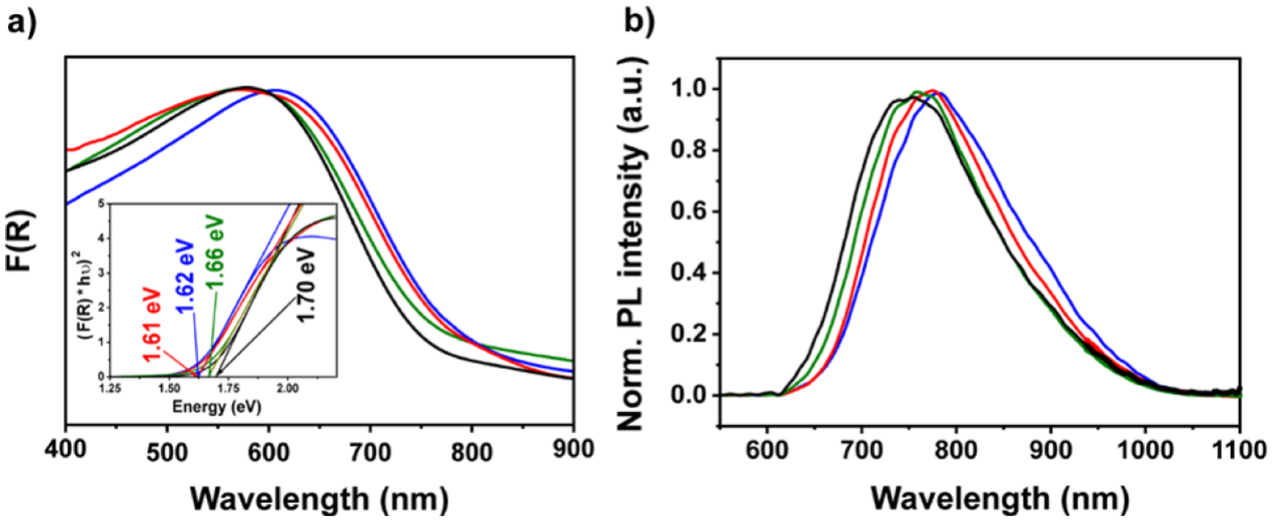
(a) F­(R) spectra with Tauc plots in inset and (b) PL spectra of W-A-H (black), W-A-Cl (green), W-A-Br (blue), W-A-I (red).

To further investigate the optical properties of the materials, photoluminescence (PL) spectroscopy was performed on the series of W-A-X COFs (Figure 2b). All anthracene-based frameworks exhibit strong emission in the red to near-infrared region, with emission maxima at 755 nm for W-A-H, 757 nm for W-A-Cl, 771 nm for W-A-Br, and 776 nm for W-A-I. Consistent with the trend observed in the UV–vis spectra, the emission profiles display a gradual redshift with increasing halogen atomic size, indicating enhanced π-conjugation and modulated electronic interactions within the framework. Additionally, PL measurements were performed for the W-TA COF to serve as a reference (Figure S37). In contrast to the anthracene-containing systems, W-TA exhibits a markedly blue-shifted emission maximum at 670 nm, consistent with its reduced conjugation.

Transient PL spectroscopy measurements were performed for the W-A-X COFs to investigate the excited-state lifetimes as a function of the halogen atom present in the anthracene linker, with the W-TA COF included as a reference (Figure S38). The W-A-Cl COF exhibits a significantly longer average lifetime of 7 ps, whereas the lifetimes of the other COFs fall below the resolution limit of 3 ps. Chlorine is the most electronegative halogen among those used in these COF systems, and the extended luminescence lifetime can be attributed to its enhanced electron-withdrawing character. The combination of the A-Cl-CHO linker with an electron donor such as W-NH_2_ enhances donor–acceptor interactions within the COF, thereby stabilizing the excited state and extending its lifetime.^83^

### 3.3 DFT and TD-DFT Calculations

To understand the observed redshift in UV–vis absorption spectra and the corresponding decrease in optical band gap energy across the W-A-X COF series (X = H, Cl, Br, I), DFT calculations were first performed on the COF models to determine their electronic structures. The band structure of W-A-H (Figure 3b) shows typical kagome-like band features^84^ along the in-plane high-symmetry path (Γ – M – K – Γ) with moderate band widths, while the band dispersions in the out-of-plane direction (Γ – A) are significantly stronger. Consistent with the strategy of tuning anthracene’s (opto-)­electronic properties through framework integration, the electronic band gap is observed to be relatively low, indicative of semiconducting behavior. The W-A-H COF has a direct electronic band gap of 0.85 eV. The halogenated COF derivatives exhibit the same electronic band features as well as a direct band gap of similar size (W-A-Cl: 0.85 eV, W-A-Br: 0.84 eV, W-A-I: 0.85 eV; see Figure 3e and Figure S39). This similarity reflects the fact that single-atom halogen substitution constitutes only a modest perturbation for the global band topology of the framework without clearly distinguishable differences among the halogenated COFs, in contrast to stronger symmetry-breaking modifications reported for idealized kagome systems.^85^ However, the asymmetric halogenated anthracene moieties break the 3-fold symmetry that underlies the distinct kagome-band-like features. This becomes noticeable in the band structure by a gap opening at the Dirac cone at the K point in the Brillouin zone (Figure 3a) in the halogenated COFs, as well as the emergence of small band dispersions for the former flat bands (Figure 3e, highlighted in green).

**Figure 3:**
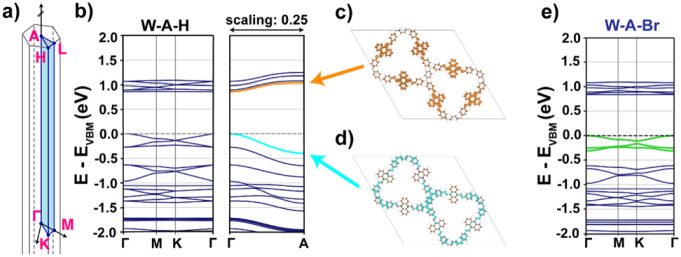
(a) Schematic representation of the high symmetry path in the Brillouin zone of a primitive hexagonal unit cell. (b) Electronic band structure of W-A-H with the partial charge densities of (c) LUMO band in orange and (d) HOMO band in blue. (e) Electronic band structure of W-A-Br with kagome-like band feature highlighted in green.

More detailed analyses of the HOMO and LUMO states show that they are highly localized on the electron-rich donor (Wurster) and electron-deficient acceptor (anthracene) fragments, as exemplarily shown in Figure 3d and c for W-A-H, respectively. The same localization tendencies are found in the halogenated COFs (see Figure S39), emphasizing the donor–acceptor character of all four COFs. The energetic comparison of the W-NH_2_ and (non)­halogenated anthracene (A-X, X = H, Cl, Br, I) building blocks in the gas phase (Figure S40) confirms the profound donor character of W-NH_2_ with an energetically high HOMO and the acceptor character of the A-X fragments (energetically low-lying LUMO).

After discussing the electronic band gap, we now focus on the optical band gap. Although the reduction in electronic band gaps of the W-A-X COFs compared to the COF building blocks W-NH_2_ and A-X (Figure S40) may show an analogous trend for optical gaps, this does not allow for a quantitative prediction of the latter. Unfortunately, explicit optical simulations of the W-A-X COFs are computationally not feasible due to size limitations. Therefore, to study the impact of anthracene halogenation on the optical properties of the W-A-X COFs, we simulated the UV–vis absorption spectrum of the halogenated and nonhalogenated anthracene molecules A-X (Figure 4a) with TD-DFT (more details in the Methods section). The spectra show a distinct redshift in the absorption maximum from anthracene (A-H) to the halogenated derivatives (A-Cl ≈ A-Br < A-I). These maxima are dominated by transitions between the HOMO and LUMO of the molecules, while other energetically close orbitals have substantially lower weights in these transitions. The energetic shift of the absorption maxima (A-Cl: −35 meV, A-Br: −40 meV, A-I: −59 meV) with respect to anthracene is consistent with the observed trend in the electronic HOMO–LUMO gaps of the halogenated anthracenes with respect to A-H (A-Cl: −31 meV, A-Br: −34 meV, A-I: −49 meV). To extrapolate these findings to the larger COF systems, a systematic extension of anthracene toward a combined anthracene-Wurster fragment of W-A-H, as present in the COF (see Figure 4b), was studied next. Already with phenyl substitution, we observe a redshift of the absorption maxima into the visible region (Figure 4c), accompanied by a significant lowering of the LUMO while the HOMO remains essentially unchanged (more details in the Supporting Information Figure S41). A similar, though smaller, redshift trend is observed for the halogenated A-X-2­(CN-Ph) fragments, (Figure S39, shifts of optical excitation energy relative to A-H-2­(CN-Ph) for Cl: −13 meV, Br: −16 meV, I: −27 meV), indicating that π-extension toward the Wurster fragment modulates the halogenation effect rather than simply adding to it. Based on these trends, we expect the COFs to inherit the fragment absorption properties, namely the strong redshift from π-extension and an additional, smaller halogen-induced redshift that fine-tunes the optical response of the W-A-X COFs, which is in agreement with the trend observed in the experimental optical spectra (Figure 2).

**Figure 4:**
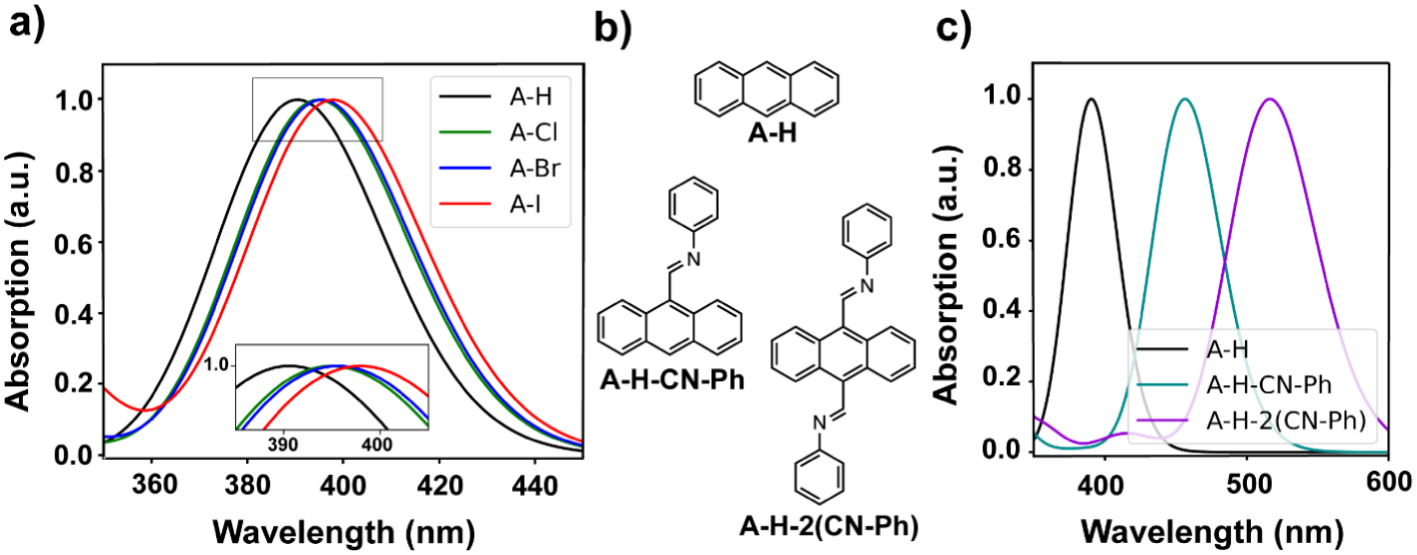
(a) Theoretical absorption spectrum of anthracene (A-H, black) and its halogenated derivatives (A-X, X = Cl (green), Br (blue), I (red)) with an inset showing the absorption maxima. (b) Molecular structure and (c) theoretical absorption spectrum of extended anthracene molecules A-H (black), A-H-CN-Ph (turquoise) and A-H-2­(CN-Ph) (purple) simulating the extension toward a combined Wurster-anthracene fragment.

Interestingly, we note that the experimental optical band gaps of the COFs (1.60–1.70 eV) do not coincide with the calculated electronic band gaps (∼0.85 eV). Typically, one would expect a significant exciton binding energy to reduce the electronic band gap toward the optical band gap. In the present case, however, the calculated electronic band gap is substantially lower than the observed optical band gap, and a negative exciton binding energy is physically implausible. To gain insight into this apparent discrepancy, we analyzed the spatial localization of the HOMO and LUMO states (see Figure 3d and c). This would indicate a charge-transfer-type transition as the lowest-energy excitation around the electronic band gap; however, such transitions are typically weaker than localized molecular-type excitations.^86^ In contrast, our theoretical results from the gradual extension of the anthracene units indicate a predominantly local, anthracene-based excitation rather than a charge-transfer-type transition. Figure S43a presents an orbital-resolved analysis of the electronic bands in W-A-H, distinguishing states originating primarily from the anthracene units from those dominated by contributions of the Wurster nodes. This analysis shows that an anthracene-dominated optical transition requires about 1.91 eV. Accounting for the exciton binding energy, this value is close to and consistent with the experimental optical band gap. Similar conclusions can be drawn for the halogenated COFs (see Figure S43b–c; local halogenated-anthracenes transitions for W-A-Cl: 1.93 eV, W-A-Br: 1.93 eV, W-A-I: 1.93 eV), while the influence of the halogen atoms is more clearly discernible in the optical simulation data.

## 4 Conclusion

We present a new strategy for tuning the structural and optical properties of covalent organic frameworks through single-atom halogen substitution of anthracene-based linkers. By systematically synthesizing and characterizing the W-A-X COF series (X = H, Cl, Br, I), we demonstrate that even minimal molecular modifications lead to pronounced changes in crystallinity, morphology, and optical response. Larger, more polarizable halogens such as bromine promote the growth of substantially larger crystalline domains compared to the unsubstituted W-A-H COF. UV–vis absorption and photoluminescence measurements reveal a clear redshift across the halogen series, in excellent agreement with DFT and TD-DFT results. Incorporation of anthracene in the framework enhances π-conjugation, directly impacting the optical response of the W-A-X COFs, while additional monohalogenation of anthracene provides a further, finely tunable modulation of the absorption maxima. Band structure analyses confirm the donor–acceptor character of the materials and show that local anthracene-based excitations dominate the optical band gap, rather than charge-transfer transitions. In addition, the markedly longer excited-state lifetime observed for W-A-Cl COF underscores the strengthened donor–acceptor interactions imparted by the more electron-withdrawing chlorine substituent. Together, these results establish halogenation as a powerful and modular approach for fine-tuning the optoelectronic properties of COFs through atom-level design. We anticipate that this effective strategy will broaden the design space for functional framework materials and enable the development of COF-based devices with tailored optical characteristics, including photocatalysts and light-management components for next-generation optoelectronic technologies.

## Supplementary Material











## Notes

Synthetic route to A-X-CHO linkers (Scheme S1); ^1^H and ^13^C NMR spectra of A-X-CHO linker and intermediate compounds (Figures S1–S15); FT-IR spectra of W-NH_2_ and A-X-CHO linkers (Figure S16); simulated structures of W-A-X COFs (Figures S17–S20); simulated structures and PXRD patterns of W-A-Br COF with different halogen positions (Figure S21); HRTEM images of W-A-H, W-A-Cl, and W-A-Br (Figures S22–S28); ESP maps of W-NH_2_ and A-X-CHO linkers (Figure S29); SEM images of W-A-X COFs (Figure S30); nitrogen sorption isotherms and pore size distributions of W-A-X COFs (Figure S31); theoretical and experimental geometric parameters of W-A-X COFs (Table S1); simulated pore size distribution of W-A-X COFs (Figure S32); FT-IR spectra of W-A-X COFs (Figure S33); ^13^C solid-state NMR spectra of W-A-X COFs (Figure S34); TGA analysis of W-A-X COFs (Figure S35); optical absorption spectra and Tauc plots of W-A-X and W-TA COFs (Figure S36); PL spectra of W-A-X and W-TA COFs (Figure S37); time-resolved PL measurements of W-A-X and W-TA COFs (Figure S38); electronic band structures of W-A-Cl and W-A-I COFs and partial charge densities of LUMO and HOMO bands of W-A-Br (Figure S39); Kohn–Sham eigenvalues of W-NH_2_ and 2-X-anthracene (X = H, Cl, Br, I) (Figure S40); Kohn–Sham eigenvalues of the extension of anthracene toward a combined anthracene-Wurster fragment (Figure S41); theoretical absorption spectrum of (non)­halogenated extensions of anthracene toward a combined Wurster-anthracene fragment (Figure S42); projected band structure of W-A-X orbitals located on the anthracene units (Figure S43) (PDF)
The crystallographic data of W-A-H (CIF)
The crystallographic data of W-A-Cl (CIF)
The crystallographic data of W-A-Br (CIF)
The crystallographic data of W-A-I (CIF)

## References

[1] Côté A. P., Benin A. I., Ockwig N. W., O’Keeffe M., Matzger A. J., Yaghi O. M. (2005). Porous, Crystalline, Covalent Organic
Frameworks. Science.

[2] Feng X., Ding X., Jiang D. (2012). Covalent Organic
Frameworks. Chem. Soc. Rev..

[3] He S., Yin B., Niu H., Cai Y. (2018). Targeted Synthesis of Visible-Light-Driven
Covalent Organic Framework Photocatalyst via Molecular Design and
Precise Construction. Appl. Catal., B.

[4] Welch G. C., Perez L. A., Hoven C. V., Zhang Y., Dang X.-D., Sharenko A., Toney M. F., Kramer E. J., Nguyen T.-Q., Bazan G. C. (2011). A Modular Molecular
Framework for Utility in Small-Molecule
Solution-Processed Organic Photovoltaic Devices. J. Mater. Chem..

[5] Liu X., Huang D., Lai C., Zeng G., Qin L., Wang H., Yi H., Li B., Liu S., Zhang M., Deng R., Fu Y., Li L., Xue W., Chen S. (2019). Recent Advances in Covalent Organic Frameworks (COFs)
as a Smart Sensing Material. Chem. Soc. Rev..

[6] Gao Q., Li X., Ning G.-H., Xu H.-S., Liu C., Tian B., Tang W., Loh K. P. (2018). Covalent Organic Framework with Frustrated
Bonding Network for Enhanced Carbon Dioxide Storage. Chem. Mater..

[7] Jung J. W., Liu F., Russell T. P., Jo W. H. (2015). Anthracene-Based Medium Bandgap Conjugated
Polymers for High Performance Polymer Solar Cells Exceeding 8% PCE
Without Additive and Annealing Process. Adv.
Energy Mater..

[8] Faraghally F. A., Musa A. F., Chen C., Chen Y., Chen Y., Yeh C., Wei T. (2024). Double Anthracene-Based Sensitizers for High-Efficiency
Dye-Sensitized Solar Cells under Both Sunlight and Indoor Light. Small Struct..

[9] Zuo J., Liu K., Harrell J., Fang L., Piotrowiak P., Shimoyama D., Lalancette R. A., Jäkle F. (2024). Near-IR Emissive
B–N Lewis Pair-Functionalized Anthracenes via Selective LUMO
Extension in Conjugated Dimer and Polymer. Angew.
Chem..

[10] Kyushin S., Suzuki Y. (2022). Cooperation of σ–π and Σ*−Π*
Conjugation in the UV/Vis and Fluorescence Spectra of 9,10-Disilylanthracene. Molecules.

[11] Aydemir M., Haykir G., Selvitopi H., Yildirim O. C., Arslan M. E., Abay B., Turksoy F. (2023). Exploring
the Potential of Anthracene
Derivatives as Fluorescence Emitters for Biomedical Applications. J. Mater. Chem. B.

[12] Zhu W., Zhao B., Fang S., Zhu H., Huang F. (2024). An Anthracene-Containing
Crown Ether: Synthesis, Host–Guest Properties and Modulation
of Solid State Luminescence. Chem. Sci..

[13] Liu Y., Zhao Z., Xu W., Gong W. (2024). Extending 2D Covalent
Organic Frameworks by Inserting Anthracene for Promoted White-Light-Mediated
Photocatalysis. Catal. Sci. Technol..

[14] Jeon J., Kim Y. J., Joo S. H., Noh H., Kwak S. K., Baek J. (2023). Benzotrithiophene-based Covalent
Organic Framework Photocatalysts
with Controlled Conjugation of Building Blocks for Charge Stabilization. Angew. Chem..

[15] Li J., Wu N. (2015). Semiconductor-Based
Photocatalysts and Photoelectrochemical Cells
for Solar Fuel Generation: A Review. Catal.
Sci. Technol..

[16] Faheem M., Aziz S., Jing X., Ma T., Du J., Sun F., Tian Y., Zhu G. (2019). Dual Luminescent Covalent Organic
Frameworks for Nitro-Explosive Detection. J.
Mater. Chem. A.

[17] Huang N., Ding X., Kim J., Ihee H., Jiang D. (2015). A Photoresponsive
Smart Covalent Organic Framework. Angew. Chem.,
Int. Ed..

[18] Ma X., Kang J., Cao W., Wu Y., Pang C., Li S., Yi Z., Xiong Y., Li C., Wang M. (2024). Anthracene-Based Dual Channel Donor-Acceptor Triazine-Containing
Covalent Organic Frameworks for Superior Photoelectrochemical Sensing. J. Colloid Interface Sci..

[19] Cheng Y., Xin J., Xiao L., Wang X., Zhou X., Li D., Gui B., Sun J., Wang C. (2023). A Fluorescent Three-Dimensional Covalent
Organic Framework Formed by the Entanglement of Two-Dimensional Sheets. J. Am. Chem. Soc..

[20] Lin M. C., Kuo S. W., Mohamed M. G. (2024). High-Performance
Anthracene-Linked
Covalent Triazine Frameworks with Dual Functions for CO2 Capture and
Supercapacitor Applications. Mater. Adv..

[21] Zhao L., Gao Y., Fu X., Chen Y., Zhang B., Xuan F. (2025). Photodual-Responsive
Anthracene-Based 2D Covalent Organic Framework for Optoelectronic
Synaptic Devices. Small Methods.

[22] Haldar S., Chakraborty D., Roy B., Banappanavar G., Rinku K., Mullangi D., Hazra P., Kabra D., Vaidhyanathan R. (2018). Anthracene-Resorcinol Derived Covalent
Organic Framework
as Flexible White Light Emitter. J. Am. Chem.
Soc..

[23] Rotter J. M., Guntermann R., Auth M., Mähringer A., Sperlich A., Dyakonov V., Medina D. D., Bein T. (2020). Highly Conducting
Wurster-Type Twisted Covalent Organic Frameworks. Chem. Sci..

[24] Zhang H.-G., Yu W.-T., Yan S.-N., Cheng C., Tao X.-T. N. (2006). N, N′,
N ′-Tetraphenyl-1,1′-Biphenyl-4,4′-Diamine. Acta Crystallogr. Sect. E.

[25] Guntermann R., Helminger D., Frey L., Zehetmaier P. M., Wangnick C., Singh A., Xue T., Medina D. D., Bein T. (2024). Tunable Isometric Donor-Acceptor
Wurster-Type Covalent Organic Framework
Photocathodes. Angew. Chem., Int. Ed..

[26] Wang L., Chakraborty J., Deng M., Sun J., Van Der
Voort P. (2024). Donor-Acceptor Pyrene-Based Covalent Organic Framework for Blue Light
Photocatalytic Oxidative Coupling of Amines. ChemCatChem.

[27] Zhai L., Cui S., Tong B., Chen W., Wu Z., Soutis C., Jiang D., Zhu G., Mi L. (2020). Bromine-Functionalized
Covalent Organic Frameworks for Efficient Triboelectric Nanogenerator. Chem.- Eur. J..

[28] Wang M., Wang Z., Shan M., Wang J., Qiu Z., Song J., Li Z. (2023). Fluorine-Substituted
Donor-Acceptor
Covalent Organic Frameworks for Efficient Photocatalyst Hydrogen Evolution. Chem. Mater..

[29] Bittner E.-A., Merkel K., Ortmann F. (2024). Engineering
the Electrostatic Potential
in a COF’s Pore by Selecting Quadrupolar Building Blocks and
Linkages. Npj 2D Mater. Appl..

[30] Yang Y., Guo L., Wang X., Li Z., Zhou W. (2024). Halogen Modified Organic
Porous Semiconductors in Photocatalysis: Mechanism, Synthesis, and
Application. Adv. Powder Mater..

[31] Cao D., Guan J., Du J., Sun Q., Ma J., Li J., Liu J., Sheng G. (2024). Halogen-Functionalized
Covalent Organic
Frameworks for Photocatalytic Cr­(VI) Reduction under Visible Light. J. Hazard. Mater..

[32] Zhao K., Qiao H., Wang S., Xu X., Wang C., Jiao M., Yang L., Kong X., Zhu Z., Qin N., Zhai L. (2024). Halogen Regulation in Vinylene-Linked
Covalent Organic
Frameworks for Efficient Photocatalytic C–H Thiolation of Quinone
Derivatives. ACS Mater. Lett..

[33] Hou H., Wu Y., Wan J., Luo R., Wu L., Zhao Y., Wu X., Lei J. (2025). P−π
Conjugation-Promoted Electrochemiluminescence
of Halogenated Covalent Organic Framework Nanoemitters. Angew. Chem., Int. Ed..

[34] Li Q., Xu Y., Lin Z., Sun Z., Liu Y. (2025). Fluorinated Covalent
Organic Frameworks Enhanced CO2 Adsorption for Boosting Photocatalytic
CO2 Reduction. Sep. Purif. Technol..

[35] Liu S., Hao C., Meng C., Liu S., Zhai W., Zhu Q., Li W., Wei S., Wang Z., Lu X. (2023). Nanoporous Fluorinated
Covalent Organic Framework for Efficient C 2 H 2 /CO 2 Separation
with High C 2 H 2 Uptake. ACS Appl. Nano Mater..

[36] Hamzehpoor E., Ruchlin C., Tao Y., Liu C.-H., Titi H. M., Perepichka D. F. (2023). Efficient Room-Temperature Phosphorescence of Covalent
Organic Frameworks through Covalent Halogen Doping. Nat. Chem..

[37] Liu Y., Guo Y., Sathishkumar N., Liu M., Li L., Sang Z., Feng R., Sun Z., Sun C., Luo M., Deng X., Lu G., Guo S. (2025). Halogen Atom-Induced
Local Asymmetric Electron in Covalent Organic Frameworks Boosts Photosynthesis
of Hydrogen Peroxide from Water and Air. Matter.

[38] Othman K. A., Azeez Y. H., Omer R. A., Kareem R. O. (2024). Theoretical Exploration
of Halogenated Anthracene Derivatives: Unraveling Electronic and Molecular
Insights. Kondens. Sredy i Mezhfaznye Granitsy
= Condens. Matter Interphases.

[39] Kupgan G., Liyana-Arachchi T. P., Colina C. M. (2017). NLDFT Pore Size Distribution in Amorphous
Microporous Materials. Langmuir.

[40] Kresse G., Hafner J. (1994). Ab Initio Molecular-Dynamics Simulation of the Liquid-Metal–Amorphous-Semiconductor
Transition in Germanium. Phys. Rev. B.

[41] Kresse G., Hafner J. (1993). Ab Initio Molecular Dynamics for Liquid Metals. Phys. Rev. B.

[42] Kresse G., Furthmüller J. (1996). Efficient Iterative Schemes for Ab Initio Total-Energy
Calculations Using a Plane-Wave Basis Set. Phys.
Rev. B.

[43] Kresse G., Furthmüller J. (1996). Efficiency of Ab-Initio Total Energy
Calculations for
Metals and Semiconductors Using a Plane-Wave Basis Set. Comput. Mater. Sci..

[44] Kresse G., Joubert D. (1999). From Ultrasoft Pseudopotentials
to the Projector Augmented-Wave
Method. Phys. Rev. B.

[45] Blöchl P. E. (1994). Projector
Augmented-Wave Method. Phys. Rev. B.

[46] Perdew J. P., Burke K., Ernzerhof M. (1996). Generalized Gradient Approximation
Made Simple. Phys. Rev. Lett..

[47] Grimme S., Ehrlich S., Goerigk L. (2011). Effect of the Damping Function in
Dispersion Corrected Density Functional Theory. J. Comput. Chem..

[48] Krukau A. V., Vydrov O. A., Izmaylov A. F., Scuseria G. E. (2006). Influence of the
Exchange Screening Parameter on the Performance of Screened Hybrid
Functionals. J. Chem. Phys..

[49] Bechstedt, F. Many-Body Approach to Electronic Excitations; Springer: Berlin Heidelberg, 2015, Vol. 181. DOI: 10.1007/978-3-662-44593-8.

[50] Frisch, M. J. ; Trucks, G. W. ; Schlegel, H. B. ; Scuseria, G. E. ; Robb, M. A. ; Cheeseman, J. R. ; Scalmani, G. ; Barone, V. ; Petersson, G. A. ; Nakatsuji, H. , Gaussian 16, Revision B.01; Gaussian, Inc.: Wallingford CT, 2016.

[51] Weigend05. Gaussian.Com. https://gaussian.com/glossary/weigend05/%0Ahttps://gaussian.com/glossary/weigend06/%0A. accessed 11 April 2026.

[52] Weigend06. Gaussian.Com. https://gaussian.com/glossary/weigend06/. accessed 11 April 2026.

[53] Otto, P. Fluorescent Cytotoxic Compounds Specific for the Cellular Polyamine Transport System; University of Central Florida, 2013.

[54] Evans A. M., Parent L. R., Flanders N. C., Bisbey R. P., Vitaku E., Kirschner M. S., Schaller R. D., Chen L. X., Gianneschi N. C., Dichtel W. R. (2018). Seeded Growth of Single-Crystal Two-Dimensional Covalent
Organic Frameworks. Science.

[55] Keller N., Bein T. (2021). Optoelectronic Processes
in Covalent Organic Frameworks. Chem. Soc. Rev..

[56] Yang B., Abyzov A. S., Zhuravlev E., Gao Y., Schmelzer J. W. P., Schick C. (2013). Size and Rate Dependence
of Crystal Nucleation in Single
Tin Drops by Fast Scanning Calorimetry. J. Chem.
Phys..

[57] Zheng Y. (2024). Size-Independent
Nucleation and Growth Model of Potassium Sulfate from Supersaturated
Solution Produced by Stirred Crystallization. Molecules.

[58] Seidler M., Li N. K., Luo X., Xuan S., Prendergast D., Zuckermann R. N., Balsara N. P., Jiang X. (2022). The Importance of the
σ-Hole in the Self-Assembly of Halogenated Polypeptoids. Microsc. Microanal..

[59] Donald, K. J. Sigma Hole Supported Interactions: Qualitative Features, Various Incarnations, and Disputations. In Exploring Chemical Concepts Through Theory and Computation; Wiley, 2024; pp. 285–316. DOI: 10.1002/9783527843435.ch11.

[60] Uran E., Fotović L., Bedeković N., Stilinović V., Cinčić D. (2021). The Amine
Group as Halogen Bond Acceptor in Cocrystals
of Aromatic Diamines and Perfluorinated Iodobenzenes. Crystals.

[61] Ding X.-H., Chang Y.-Z., Ou C.-J., Lin J.-Y., Xie L.-H., Huang W. (2020). Halogen Bonding in the Co-Crystallization of Potentially Ditopic
Diiodotetrafluorobenzene: A Powerful Tool for Constructing Multicomponent
Supramolecular Assemblies. Natl. Sci. Rev..

[62] Nemec V., Lisac K., Liović M., Brekalo I., Cinčić D. (2020). Exploring
the Halogen-Bonded Cocrystallization Potential of a Metal-Organic
Unit Derived from Copper­(Ii) Chloride and 4-Aminoacetophenone. Materials.

[63] Ibrahim M. A. A., Mahmoud A. M. M., Shehata M. N. I., Saeed R. R. A., Moussa N. A. M., Sayed S. R. M., Abd El-Rahman M. K., Shoeib T. (2024). σ-Hole Site-Based Interactions within Hypervalent
Pnicogen, Halogen, and Aerogen-Bearing Molecules with Lewis Bases:
A Comparative Study. ACS Omega.

[64] Lázaro I. A., Almora-Barrios N., Tatay S., Martí-Gastaldo C. (2021). Effect of
Modulator Connectivity on Promoting Defectivity in Titanium–Organic
Frameworks. Chem. Sci..

[65] Hou S., Zhang G., Qiao Z., Bai Y., Di H., Hua Y., Hao T., Xu H. (2025). Diffusion/Modulator
Dual-Mediated
Solid-Liquid/Vapor Interfacial Synthesis of Crystalline Covalent Organic
Framework Membranes. Angew. Chem., Int. Ed..

[66] Lang T., Zhang X., Meng L., Zeng Y. (2016). Mutual Enhancing Effects
of the σ-Hole Interactions and Halogen/Hydrogen-Bonded Interactions
in the Iodine-Ylide Containing Complexes. Struct.
Chem..

[67] Dautzenberg E., Claassen F. W., de Smet L. C. P. M. (2023). Functionalized
Modulators in Imine-Linked
Covalent Organic Frameworks (COFs). Microporous
Mesoporous Mater..

[68] Calik M., Sick T., Dogru M., Döblinger M., Datz S., Budde H., Hartschuh A., Auras F., Bein T. (2016). From Highly Crystalline to Outer
Surface-Functionalized Covalent Organic Frameworks—A Modulation
Approach. J. Am. Chem. Soc..

[69] Safarifard V., Morsali A. (2014). Influence of an Amine
Group on the Highly Efficient
Reversible Adsorption of Iodine in Two Novel Isoreticular Interpenetrated
Pillared-Layer Microporous Metal–Organic Frameworks. CrystEngComm.

[70] Zhang N., Wang T., Wu X., Jiang C., Chen F., Bai W., Bai R. (2018). Self-Exfoliation
of 2D Covalent Organic Frameworks:
Morphology Transformation Induced by Solvent Polarity. RSC Adv..

[71] Varadwaj A., Marques H. M., Varadwaj P. R. (2019). Is the
Fluorine in Molecules Dispersive?
Is Molecular Electrostatic Potential a Valid Property to Explore Fluorine-Centered
Non-Covalent Interactions?. Molecules.

[72] Sarkisov L., Bueno-Perez R., Sutharson M., Fairen-Jimenez D. (2020). Materials
Informatics with PoreBlazer v4.0 and the CSD MOF Database. Chem. Mater..

[73] Song B., Sikma R. E., McKeown C., Leung K., Sava Gallis D. F., Ilgen A. G. (2025). Influence of Linker
Halogenation on Selective Lanthanide
Adsorption in Nanoporous Zr­(IV)-Based Metal–Organic Frameworks. ACS Appl. Nano Mater..

[74] Willems T. F., Rycroft C. H., Kazi M., Meza J. C., Haranczyk M. (2012). Algorithms
and Tools for High-Throughput Geometry-Based Analysis of Crystalline
Porous Materials. Microporous Mesoporous Mater..

[75] Waller P. J., Alfaraj Y. S., Diercks C. S., Jarenwattananon N. N., Yaghi O. M. (2018). Conversion of Imine to Oxazole and Thiazole Linkages
in Covalent Organic Frameworks. J. Am. Chem.
Soc..

[76] Zhang Y., Shi W., Zhao Y., Zhang C., Zhi Y. (2023). Linkage Design in Two-Dimensional
Covalent Organic Frameworks for High Iodine Uptake. Macromol. Rapid Commun..

[77] Palleros, D. R. Infrared Spectroscopy” in Experimental Organic Chemistry; Wiley: New York, 2000.

[78] Alcaraz
de la Osa R., Iparragirre I., Ortiz D., Saiz J. M. (2020). The Extended
Kubelka–Munk Theory and Its Application to Spectroscopy. ChemTexts.

[79] Fu C.-F., Zhao C., Zheng Q., Li X., Zhao J., Yang J. (2020). Halogen Modified Two-Dimensional
Covalent Triazine Frameworks as
Visible-Light Driven Photocatalysts for Overall Water Splitting. Sci. China: Chem..

[80] Varadwaj P. R., Varadwaj A., Marques H. M., Yamashita K. (2018). Halogen in
Materials Design: Chloroammonium Lead Triiodide Perovskite (ClNH3PbI3)
a Dynamical Bandgap Semiconductor in 3D for Photovoltaics. J. Comput. Chem..

[81] Labdelli A., Bendahma F., Mana M., Benderdouche N. (2023). Evolution
of Electronic Bandgap by Anion Variation to Explore Niobium New Halide
Double Perovskites Cs2GeNbX6 (X = Cl, Br, I) for Solar Cells and Thermoelectric
Applications: First Principles Analysis. Rev.
Mex. Fís..

[82] Mbarek M., Chemek M., Wery J., Duvail J. L., Alimi K. (2014). The Effect
of Conjugation Length Distribution on the Properties of Modified PPV. J. Phys. Chem. Solids.

[83] Ma B., Lin X., Zhu T., Zheng X., Zhu J. (2024). Donor–Acceptor
Type COFs with Multiple Fluorine Groups as Electron Storage Units
to Promote Antimicrobial Performance. Colloids
Surf., B.

[84] Jiang W., Ni X., Liu F. (2021). Exotic Topological
Bands and Quantum States in Metal–Organic
and Covalent–Organic Frameworks. Acc.
Chem. Res..

[85] Merkel K., Greiner J., Ortmann F. (2023). Understanding the Electronic
Pi-System
of 2D Covalent Organic Frameworks with Wannier Functions. Sci. Rep..

[86] Panhans M., Hutsch S., Benduhn J., Schellhammer K. S., Nikolis V. C., Vangerven T., Vandewal K., Ortmann F. (2020). Molecular
Vibrations Reduce the Maximum Achievable Photovoltage in Organic Solar
Cells. Nat. Commun..

