# Supplementary material for: Single-Atom Tuning of Structural and Optoelectronic Properties in Halogenated Anthracene-Based Covalent Organic Frameworks
Source: ACS Omega. 2026 May 19;11(21):31432–42. doi: 10.1021/acsomega.6c01720 (PMC13234796; doi:10.1021/acsomega.6c01720)
Supplement: Supplementary file 1 [file ao6c01720_si_001.pdf]

Supporting Information

Single-Atom Tuning of Structural and Optoelectronic Properties in Halogenated Anthracene-Based Covalent Organic Frameworks

Klaudija Paliušytė[#a], Laura Fuchs[#b], Zehua Xu[a], Kuangjie Liu[a], Kornel Roztocki[c], Shuo Sun[a], Hendrik Zipse[a], Achim Hartschuh[a], Frank Ortmann[b]* and Jenny Schneider[a]*

[a]Department of Chemistry and Center for NanoScience (CeNS), University of Munich (LMU), Munich 81377, Germany
[b]Department of Chemistry, TUM School of Natural Sciences, and Atomistic Modeling Center, Munich Data Science Institute, Technische Universität München, 85748 Garching b. München, Germany
[c]Faculty of Chemistry, Adam Mickiewicz University, 61-614 Poznań, Poland

[#]K.P. and L.F. contributed equally.

1. Linker synthesis 2
1.1. 2-Chloro-9,10-dihydro-9,10-[4,5]epidioxoloanthracen-13-one (A-Cl-epO) 3
1.2. 2-Chloro-9,10-dihydro-9,10-ethanoanthracene-11,12-diol (A-Cl-$(OH)_2$) 4
1.3. 2-Chloroanthracene-9,10-dicarbaldehyde (A-Cl-CHO) 6
1.4. 2-Bromo-9,10-dihydro-9,10-[4,5]epidioxoloanthracen-13-one (A-Br-epO) 9
1.5. 2-Bromo-9,10-dihydro-9,10-ethanoanthracene-11,12-diol (A-Br-$(OH)_2$) 10
1.6. 2-Bromoanthracene-9,10-dicarbaldehyde (A-Br-CHO) 12
1.7. 2-Iodoantracene (A-I) 14
1.8. 2-Iodo-9,10-dihydro-9,10-[4,5]epidioxoloanthracen-13-one (A-I-epO) 16
1.9. 2-Iodo-9,10-dihydro-9,10-ethanoanthracene-11,12-diol (A-I-$(OH)_2$) 17
1.10. 2-Iodoanthracene-9,10-dicarbaldehyde (A-I-CHO) 19
1.11. FT-IR analysis of linkers 22
2. COF synthesis 22
2.1. W-A-H COF synthesis 22
2.2. W-A-Cl COF synthesis 23
2.3. W-A-Br COF synthesis 24
2.4. W-A-I COF synthesis 25
2.5. W-TA COF synthesis 26
3. Simulations of different halogen positions 27
4. HRTEM images 28

5. ESP calculations 33
6. SEM images 34
7. Sorption and porosity parameters 35
8. FT-IR analysis of COFs 37
9. Solid-state ^{13}C NMR of COFs 37
10. TGA analysis 38
11. Experimental optical properties 38
12. Calculated optical properties 40
13. References 42

1. Linker synthesis

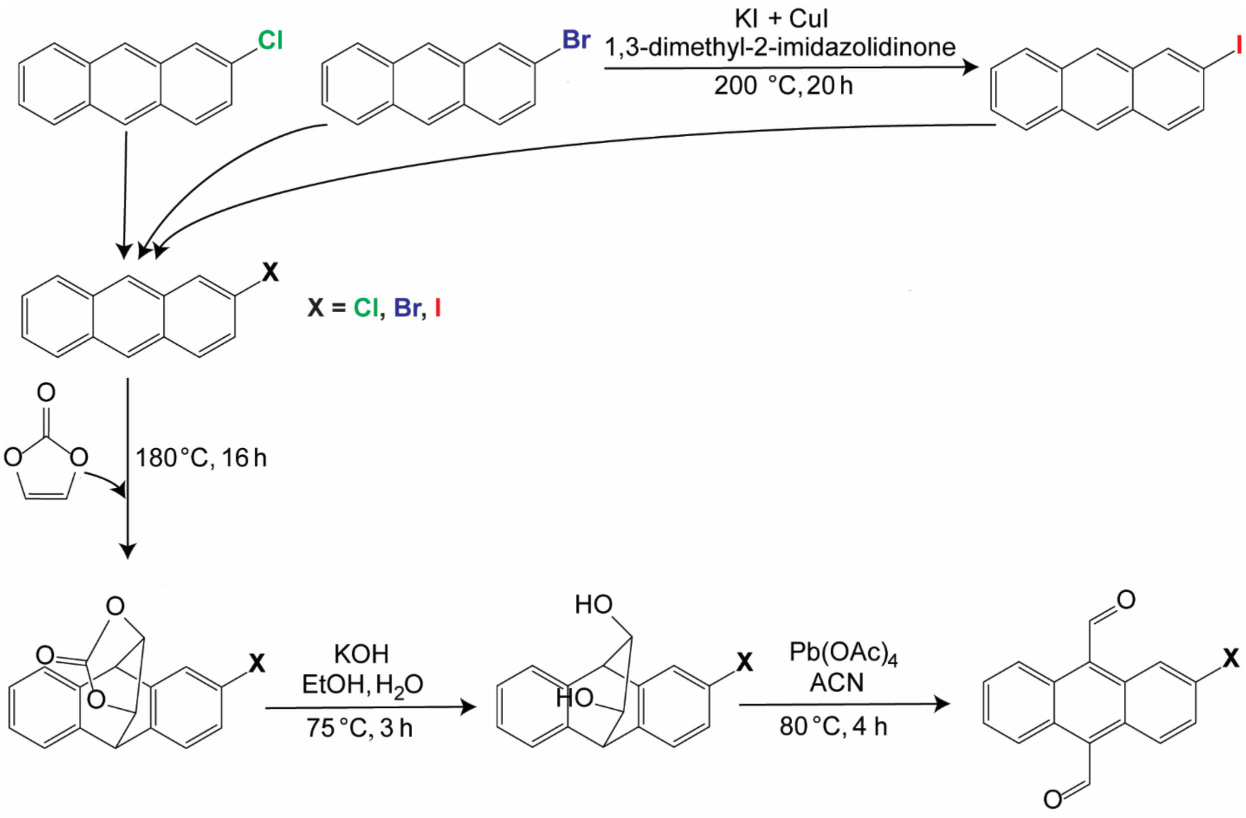

Scheme S1. Synthetic route to 2-halogen-9,10-anthracenedialdehyde (A-X-CHO, X = Cl, Br, I) linkers.

1.1. 2-Chloro-9,10-dihydro-9,10-[4,5]epidioxoloanthracen-13-one (A-Cl-epO)

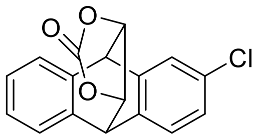

The following procedure was adapted from the previously published literature.[1] 2-Chloroanthracene (1.175 g, 5.541 mmol, 1 equiv.) and vinylene carbonate (3.484 g, 40.452 mmol, 7.3 equiv.) were heated under reflux with stirring for 18 hours, slowly forming a dark brown solution. The consumption of 2-chloroanthracene was monitored by thin-layer chromatography (CH_2Cl_2/hexane 1:49 , R=0.30). The mixture underwent rotary evaporation under high vacuum to remove the excess vinylene carbonate, providing 2-chloro-9,10-dihydro-9,10-[4,5]epidioxoloanthracen-13-one (A-Cl-epO) as a light-brown solid (1.62 g, 5.43 mmol, 98.1 %). The product was used for the further reaction without additional purification.

^{1}H NMR (400 MHz, $CDCl_3$) δ 7.35 – 7.11 (m, 7H), 4.86 – 4.75 (m, 2H), 4.67 – 4.58 (m, 2H).

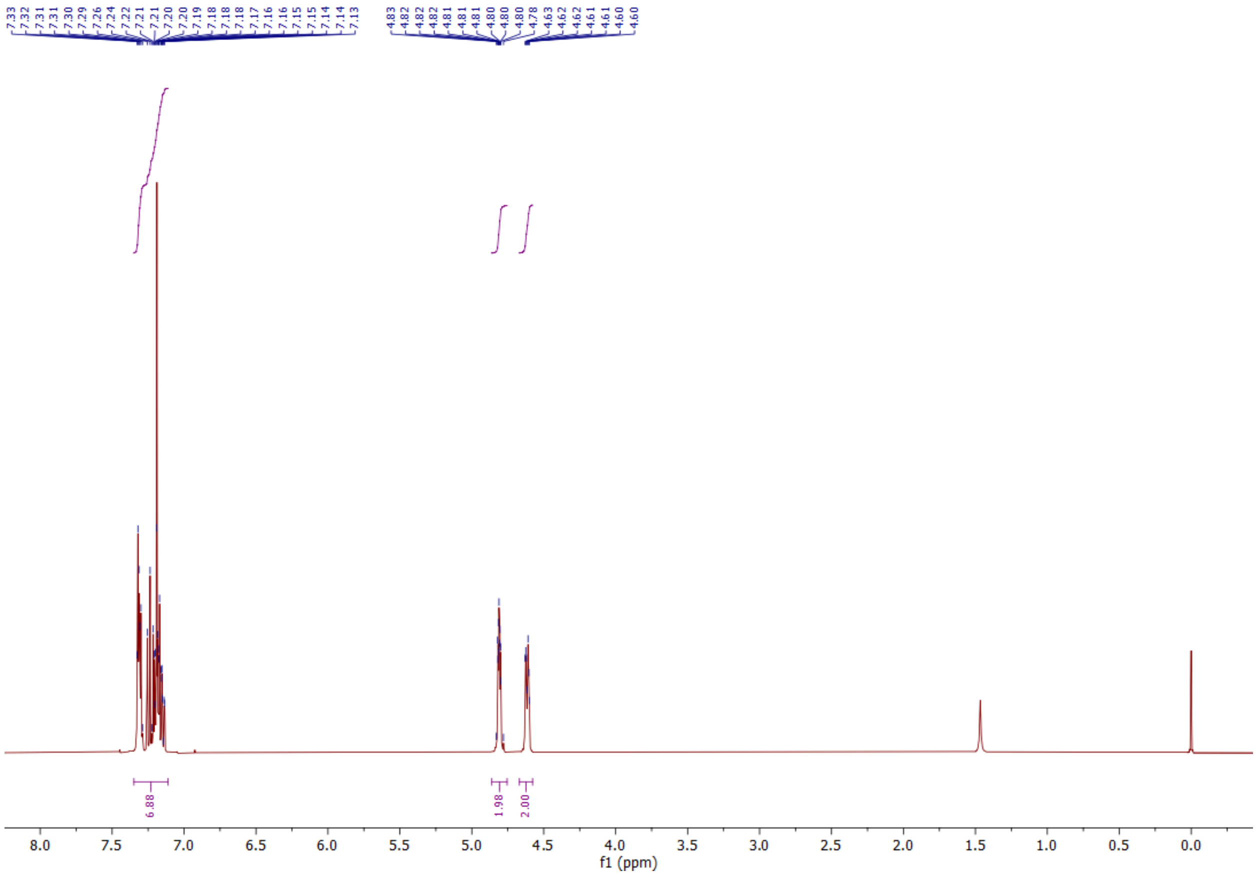

Figure S1. ^{1}H NMR spectra (400 MHz in $CDCl_3$) of A-Cl-epO.

1.2. 2-Chloro-9,10-dihydro-9,10-ethanoanthracene-11,12-diol (A-Cl-(OH)$_2$)

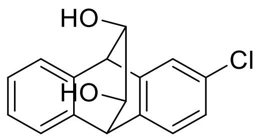

The following procedure was adapted from the previously published literature.[1] Solid potassium hydroxide (1.175 g, 5.225 mmol, 4 equiv.), deionized water (19.60 mL), and absolute ethanol (2.1 mL) were added to the A-Cl-epO (1.568 g, 5.225 mmol, 1 equiv.). The solution was stirred at 75° C for 3 hours. The consumption of the A-Cl-epO was monitored through thin-layer chromatography (100% CH_2Cl_2, R=0.30). Afterwards, the solution underwent rotary evaporation under reduced pressure to remove the ethanol and roughly half of the water volume. Additional water (39.2 mL) was added to the solution and the solution was stirred at room temperature for

one hour, resulting in the formation of a light-tan solid. The contents were vacuum-filtered and then washed with deionized water. The vacuum-filtration receiving flask was changed and the solid was washed with ethyl acetate through the filter paper. The ethyl acetate was removed through rotary evaporation, leaving a yellow solid residue. The product was purified through column chromatography (hexane/ethyl acetate 1:1, R=0.55 & R=0.50), providing two isomers of 2-chloro-9,10-dihydro-9,10-ethanoanthracene-11,12-diol (A-Cl-$(OH)_2$) as a white solid (1.006 g, 3.70 mmol, 70.8 %).

^{1}H NMR (400 MHz, $CDCl_3$) δ (ppm): 7.30 (d, J = 2.0 Hz, 1H), 7.27 – 7.19 (m, 3H), 7.15 – 7.07 (m, 3H), 4.36 – 4.29 (m, 2H), 4.04 – 3.97 (m, 2H)

^{1}H NMR (400 MHz, $CDCl_3$) δ (ppm): 7.30 (dd, J = 5.4, 3.3 Hz, 2H), 7.26 – 7.04 (m, 5H), 4.33 (dd, J = 7.3, 2.5 Hz, 2H), 4.03 – 3.95 (m, 2H)

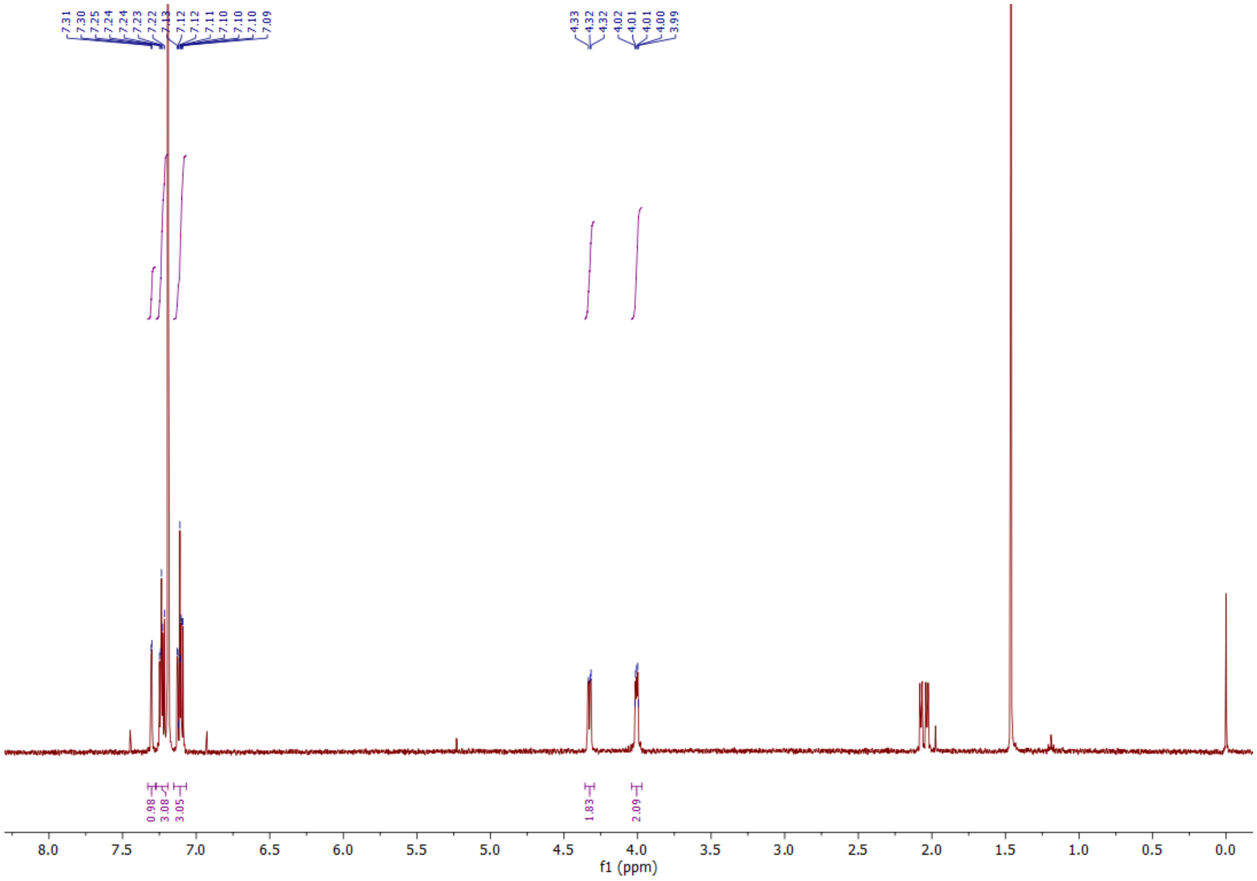

Figure S2. ^{1}H NMR spectra (400 MHz in $CDCl_3$) of A-Cl-$(OH)_2$ (isomer 1).

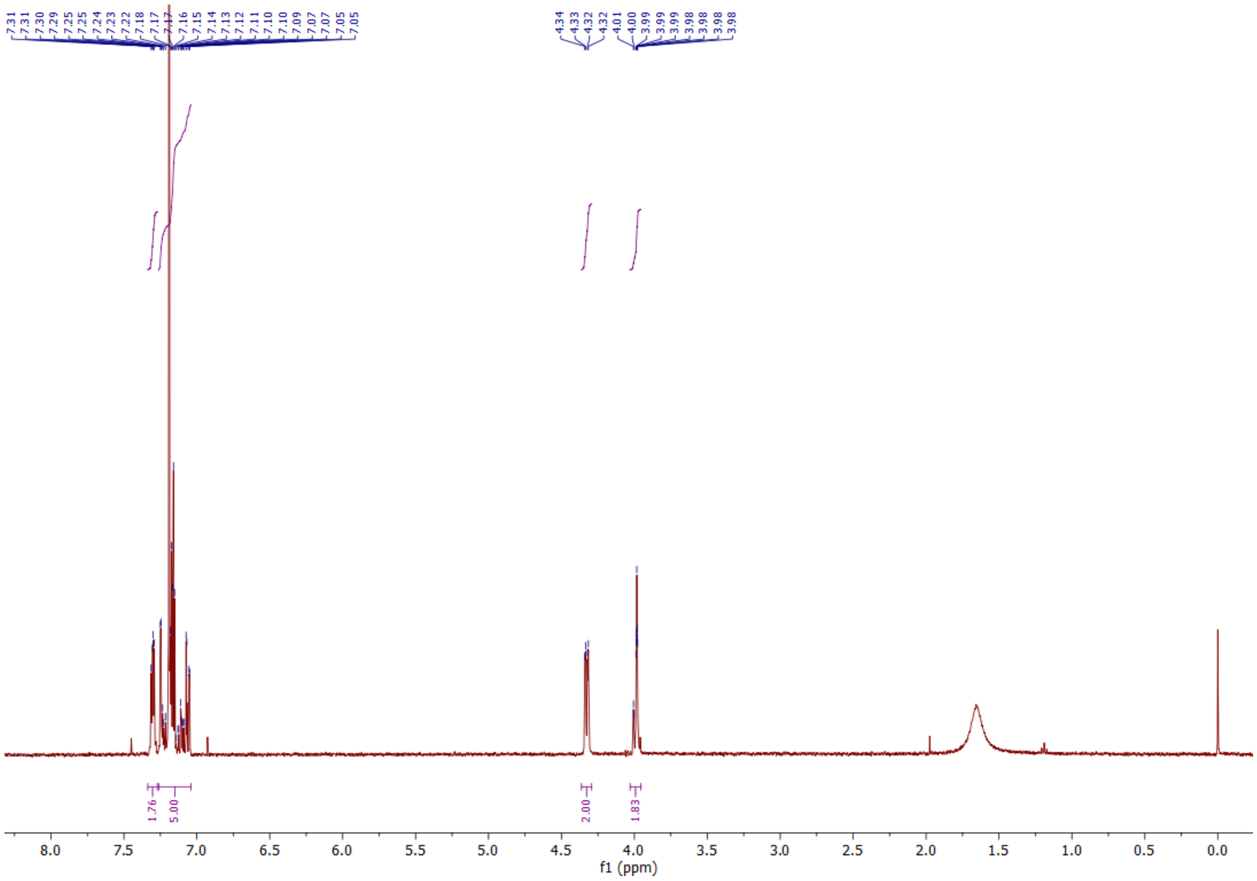

Figure S3. ^{1}H NMR spectra (400 MHz in $CDCl_3$) of A-Cl-$(OH)_2$ (isomer 2).

1.3. 2-Chloroanthracene-9,10-dicarbaldehyde (A-Cl-CHO)

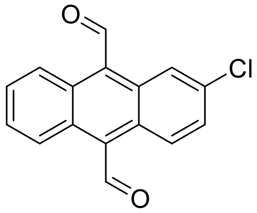

The following procedure was adapted from the previously published literature.[1] The A-Cl-$(OH)_2$ (0.995 g, 3,66 mmol, 1 equiv.) was dissolved in anhydrous acetonitrile (63 mL) and heated to reflux with stirring, forming a transparent, light-tan solution. Lead tetraacetate (3.243 g, 7.316 mmol, 2 equiv.) was added portion-wise to the solution over a period of 5 minutes, quickly turning the solution opaque and brown in color. The solution was then stirred at reflux for 4 hours, resulting in the formation of a dark-orange precipitate within the brown solution. The consumption of the A-Cl-$(OH)_2$ was monitored through thin-layer chromatography (CH_2Cl_2/ethyl acetate 8:2, R=0.50 & R=0.33). After the reaction was complete, the reaction mixture was concentrated via rotary

evaporation and gave a burgundy residue. 10% Aqueous sodium carbonate (70 mL) was added to the residue, allowing the residue to be suspended in the aqueous solution. The organic contents were extracted from the aqueous layer using dichloromethane. The opaque, yellow organic layer was dried over anhydrous sodium sulfate, filtered, and concentrated to give a yellow-orange solid residue. The residue was purified by column chromatography (hexane/CH_2Cl_2/ethyl acetate/hexane 10:1:1, R=0.3), providing 2-chloro-9,10-anthracenedialdehyde (A-Cl-CHO) as an orange solid (0.630 g, 2.350 mmol). Yield 64.26 %.

^{1}H NMR (400 MHz, $CDCl_3$) δ 11.39 (s, 1H), 11.37 (s, 1H), 8.80 (d, J = 2.1 Hz, 1H), 8.71 – 8.60 (m, 3H), 7.72 – 7.62 (m, 2H), 7.57 (dd, J = 9.5, 2.1 Hz, 1H).

^{13}C NMR (101 MHz, $CDCl_3$) δ 193.88, 193.40, 135.30, 133.05, 132.79, 131.33, 130.41, 129.62, 128.98, 128.55, 128.06, 126.13, 124.19, 123.94, 123.14.

HRMS-EI: Measured (*m*/*z*): 268.03. Theoretical (*m*/*z*): 268.70.

Elemental analysis: Measured C (69.06 %), H (3.23 %), Cl (13.20 %). Theoretical: C (71.52 %), H (3.80 %), Cl (13.19 %).

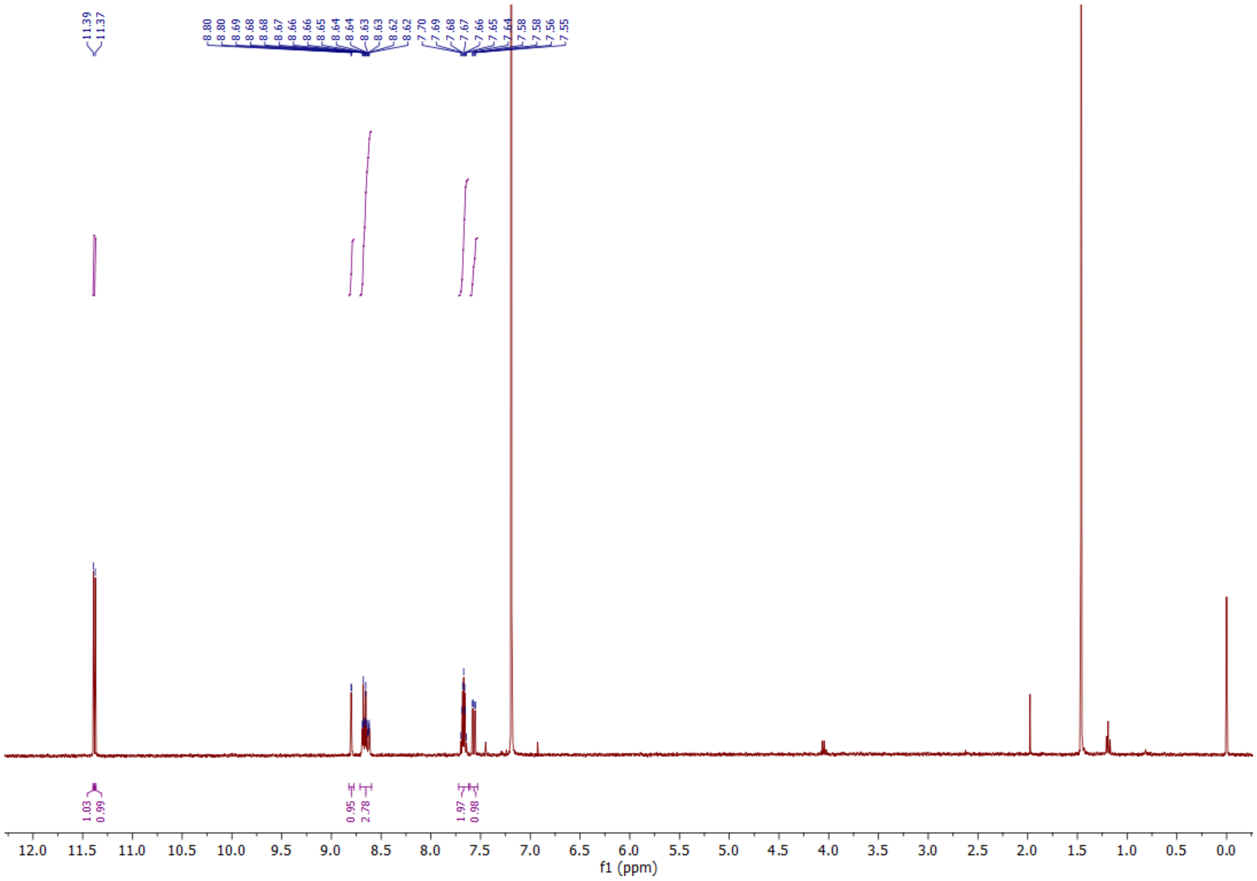

Figure S4. ^{1}H NMR spectra (400 MHz in $CDCl_3$) of A-Cl-CHO.

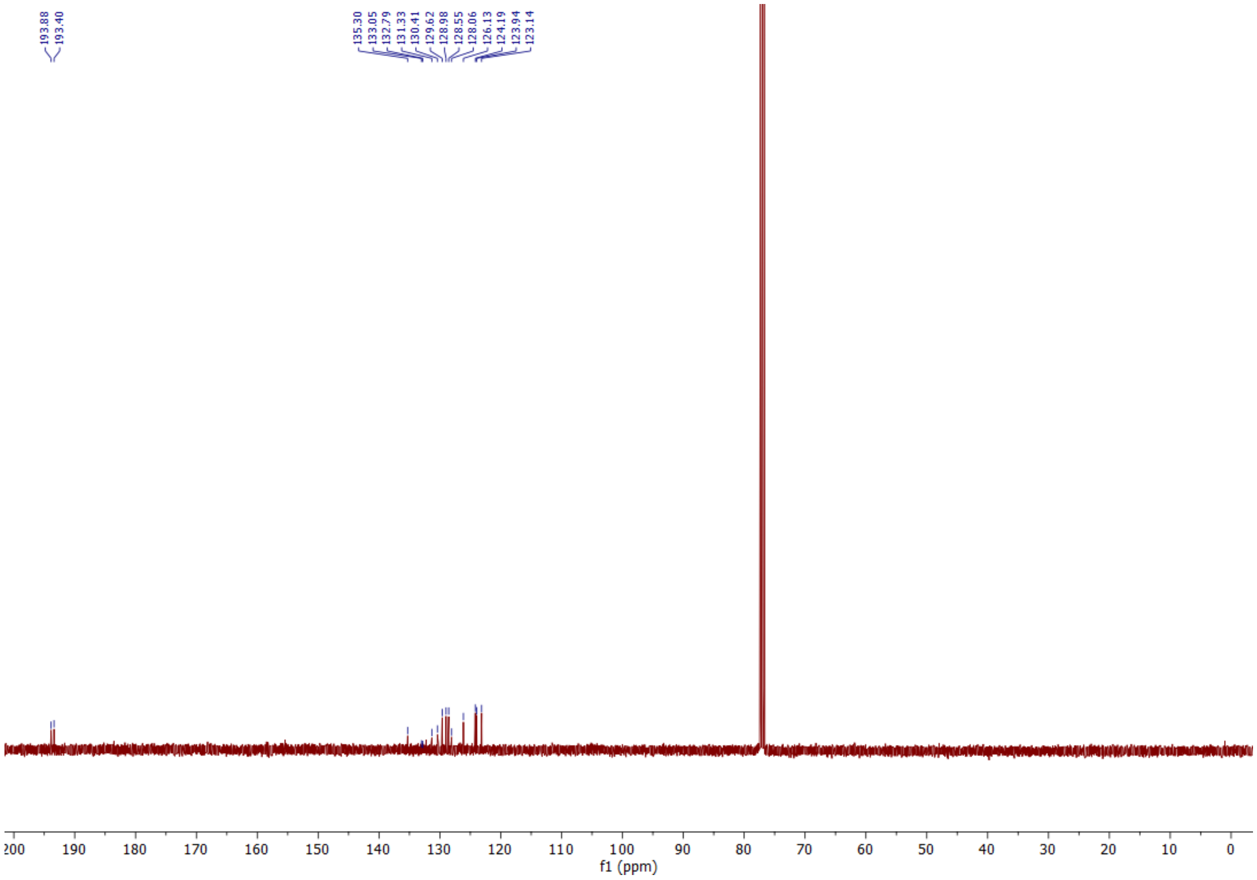

Figure S5. ^{13}C NMR spectra (101 MHz in $CDCl_3$) of A-Cl-CHO.

1.4. 2-Bromo-9,10-dihydro-9,10-[4,5]epidioxoloanthracen-13-one (A-Br-epO)

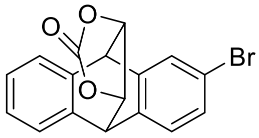

The following procedure was adapted from the previously published literature.[1] 2-Bromoanthracene (2.4 g, 9.39 mmol, 1 equiv.) and vinylene carbonate (5.9 g, 58.56 mmol, 7.3 equiv.) were heated under reflux with stirring for 18 hours, slowly forming a dark brown solution. The consumption of 2-bromoanthracene was monitored by thin-layer chromatography (CH_2Cl_2/hexane 1:49 , R=0.30). The mixture underwent rotary evaporation under high vacuum to remove the excess vinylene carbonate, providing the as 2-bromo-9,10-dihydro-9,10-[4,5]epidioxoloanthracen-13-one (A-Br-epO) light-brown solid (3.18 g, 9.30 mmol, 66.6%). The product was used for the further reaction without additional purification.

^{1}H NMR (400 MHz, $CDCl_3$) δ 7.46 (dd, J = 3.6, 1.9 Hz, 1H), 7.35 – 7.26 (m, 3H), 7.23 – 7.14 (m, 3H), 4.81 (dt, J = 3.7, 1.8 Hz, 2H), 4.61 (dq, J = 7.1, 1.6 Hz, 2H).

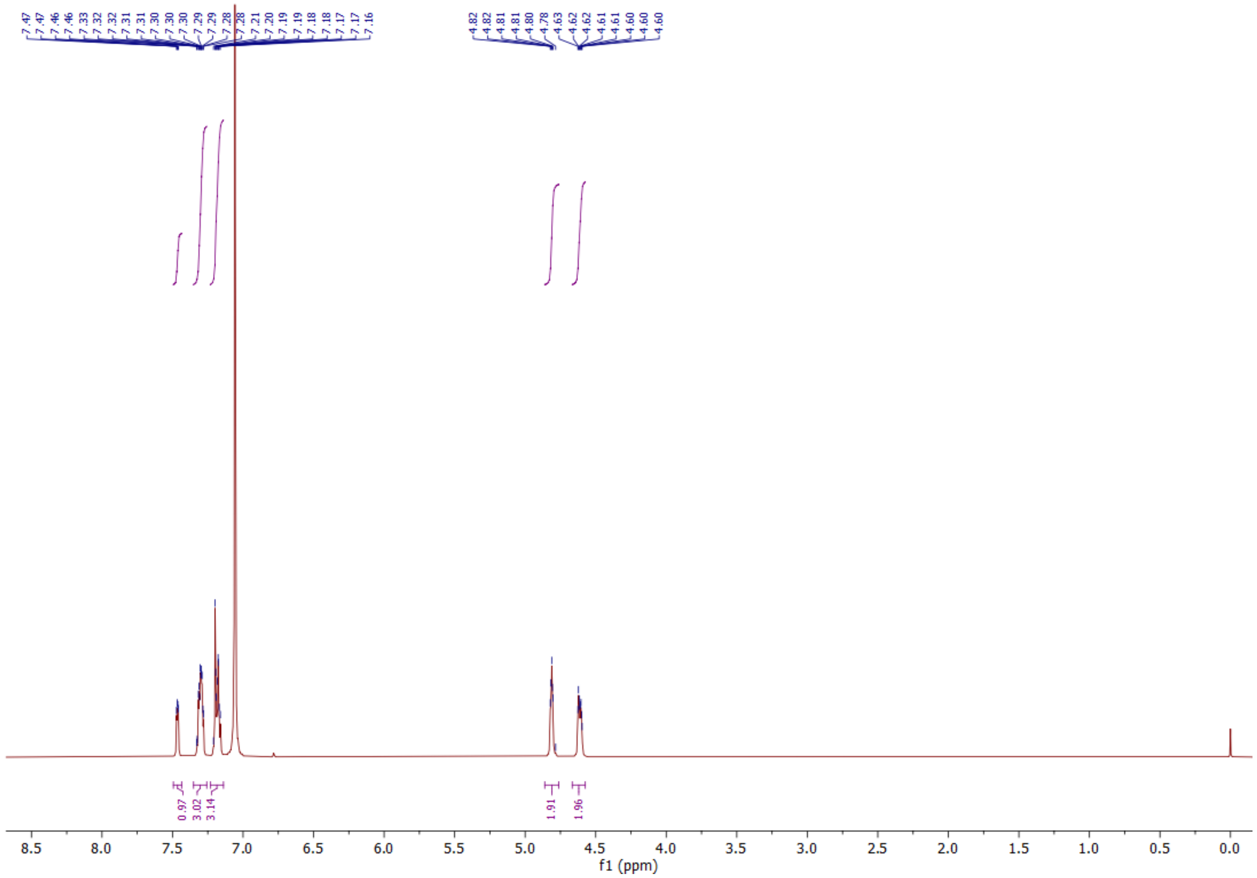

Figure S6. ^{1}H NMR spectra (400 MHz in $CDCl_3$) of A-Br-epO.

1.5. 2-Bromo-9,10-dihydro-9,10-ethanoanthracene-11,12-diol (A-Br-(OH)$_2$)

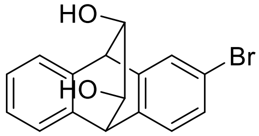

The following procedure was adapted from the previously published literature.[1] Solid potassium hydroxide (2.56 g, 45.59 mmol, 5.1 equiv.), deionized water (42.6 mL), and absolute ethanol (4.5 mL) were added to the A-Br-epO (3.06 g, 8.94 mmol, 1 equiv.). The solution was stirred at 75° C for 3 hours. The consumption of the A-Br-epO was monitored through thin-layer chromatography (100% CH_2Cl_2, R=0.30). Afterwards, the solution underwent rotary evaporation under reduced pressure to remove the ethanol and roughly half of the water volume. Additional water (85 mL) was added to the solution and the solution was stirred at room temperature for one hour, resulting

in the formation of a light-tan solid. The contents were vacuum-filtered and then washed with deionized water. The vacuum-filtration receiving flask was changed and the solid was washed with ethyl acetate through the filter paper. The ethyl acetate was removed through rotary evaporation, leaving a yellow solid residue. The product was purified through column chromatography (CH_2Cl_2/ethyl acetate 9:1, R=0.30 & R=0. 15), providing two isomers of 2-bromo-9,10-dihydro-9,10-ethanoanthracene-11,12-diol (A-Br-$(OH)_2$) as a white solid (2.01 g, 79%).

^{1}H NMR (400 MHz, $CDCl_3$) δ 7.40 (d, J = 1.9 Hz, 1H), 7.34 – 7.06 (m, 6H), 4.32 (dd, J = 6.1, 2.4 Hz, 2H), 4.01 – 3.94 (m, 2H).

Elemental analysis calculated: C (60.59 %), H (4.07 %). Theoretical: C (60.59 %). H (4.13 %).

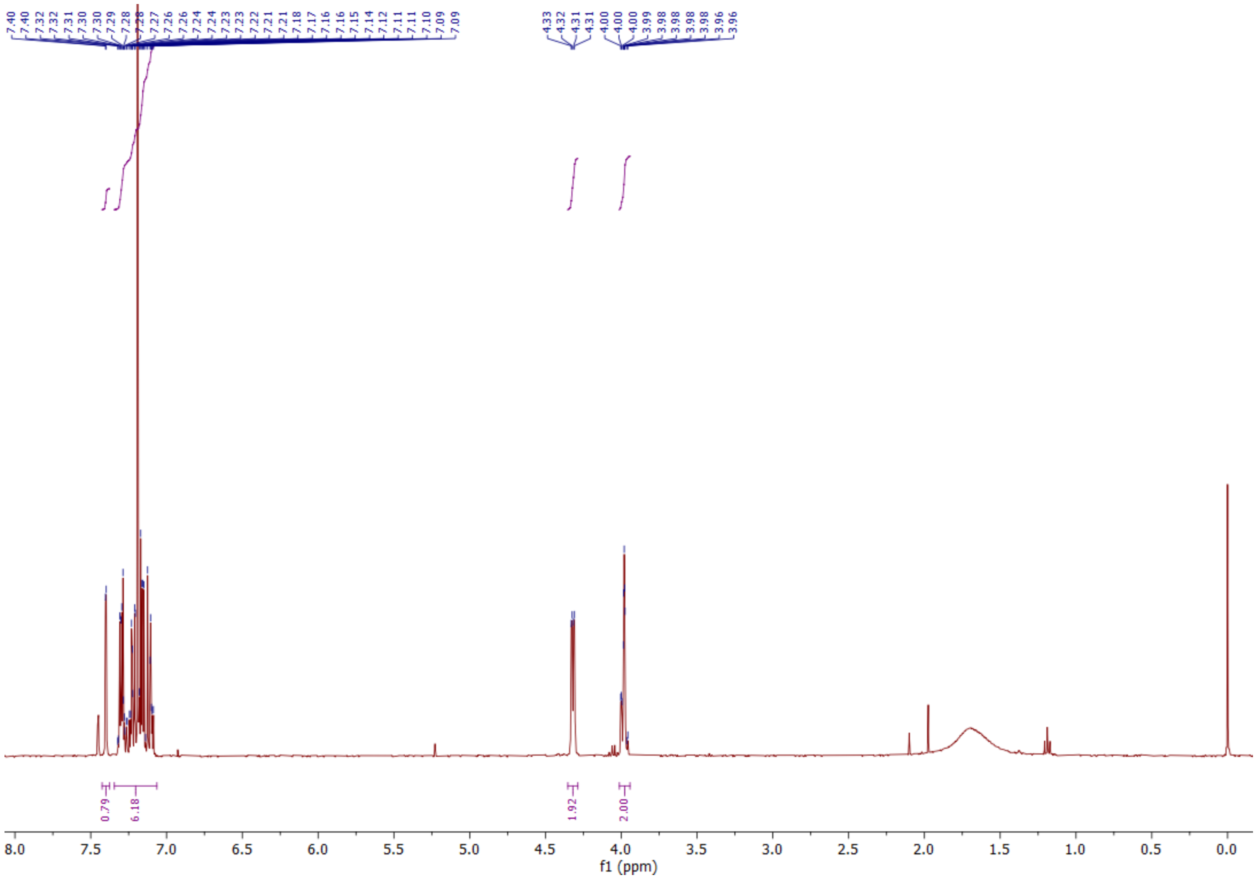

Figure S7. ^{1}H NMR spectra (400 MHz in $CDCl_3$) of A-Br-$(OH)_2$ (mixture of two isomers).

1.6. 2-Bromoanthracene-9,10-dicarbaldehyde (A-Br-CHO)

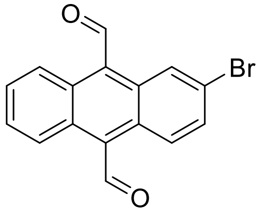

The following procedure was adapted from the previously published literature.[1] A-Br-$(OH)_2$ (2.00 g, mmol, 1 equiv.) was dissolved in anhydrous acetonitrile (127 mL) and heated to reflux with stirring, forming a transparent, light-tan solution. Lead tetraacetate (5.595 g, mmol, 2 equiv.) was added portion-wise to the solution over a period of 5 minutes, quickly turning the solution opaque and brown in color. The solution was then stirred at reflux for 4 hours, resulting in the formation of a dark-orange precipitate within the brown solution. The consumption of the A-Br-$(OH)_2$ was monitored through thin-layer chromatography (CH_2Cl_2/ethyl acetate 8:2, R=0.50 & R=0.33). After the reaction was complete, the reaction mixture was concentrated via rotary evaporation and gave a burgundy residue. 10% Aqueous sodium carbonate (130 mL) was added to the residue, allowing the residue to be suspended in the aqueous solution. The organic contents were extracted from the aqueous layer using dichloromethane. The opaque, yellow organic layer was dried over anhydrous sodium sulfate, filtered, and concentrated to give a yellow-orange solid residue. The residue was purified by column chromatography (ethyl acetate/hexane 2:8, R=0.3), providing 2-bromo-9,10-anthracenedialdehyde (A-Br-CHO) as an orange solid (1.71 g, 5.465 mmol). Yield 86.35 %.

^{1}H NMR (400 MHz, $CDCl_3$) δ 11.39 (s, 1H), 11.37 (s, 1H), 8.97 (dd, J = 1.9, 0.5 Hz, 1H), 8.71 – 8.55 (m, 3H), 7.73 – 7.63 (m, 3H).

^{13}C NMR (101 MHz, $CDCl_3$) δ 192.75, 192.40, 131.21, 130.92, 130.14, 129.63, 129.38, 129.23, 127.95, 127.57, 127.11, 125.50, 124.96, 123.15, 122.97, 122.87.

HRMS-EI: Measured (m/z): 313.98. Theoretical (m/z): 313.99.

Elemental analysis: Measured C (60.44 %), H (2.98 %). Theoretical: C (61.37 %), H (2.90 %).

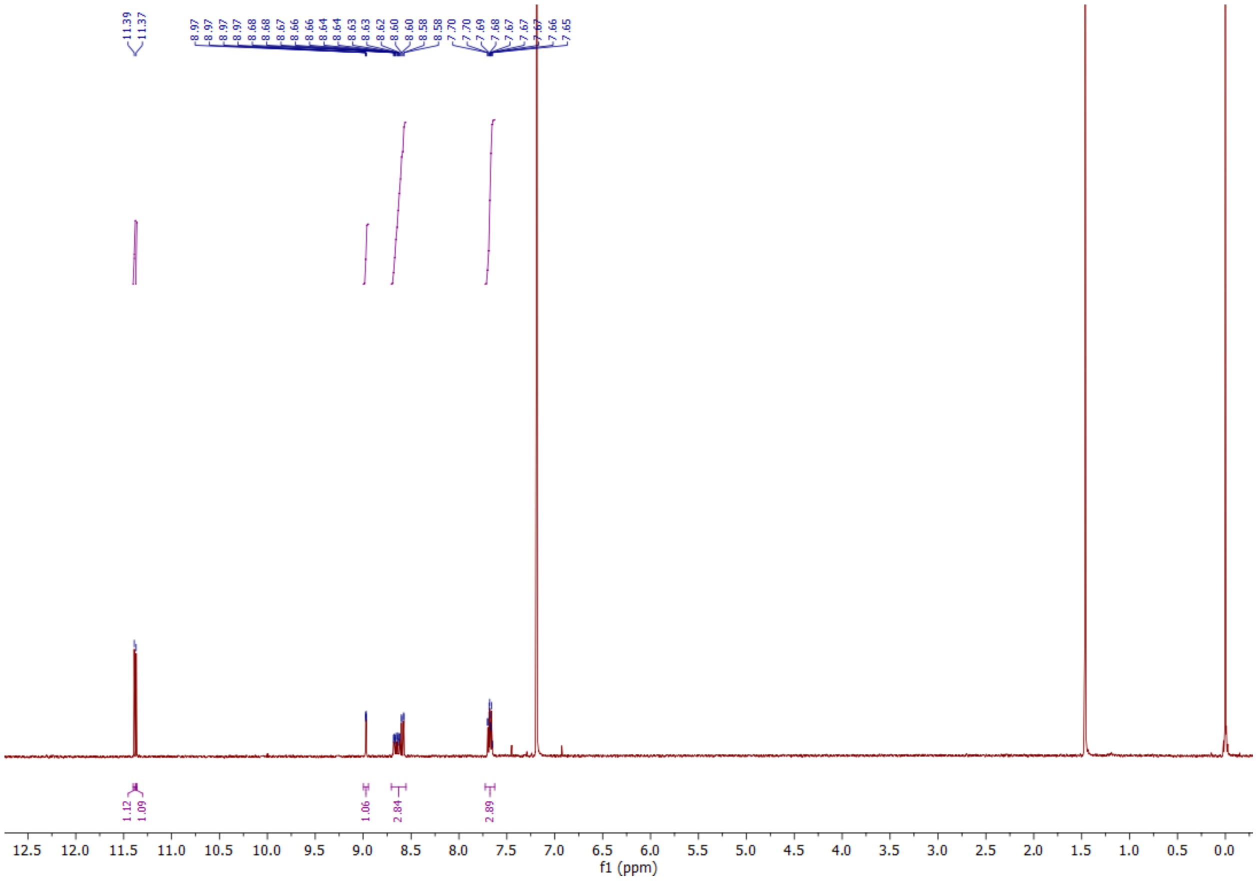

Figure S8. ^{1}H NMR spectra (400 MHz in $CDCl_3$) of A-Br-CHO.

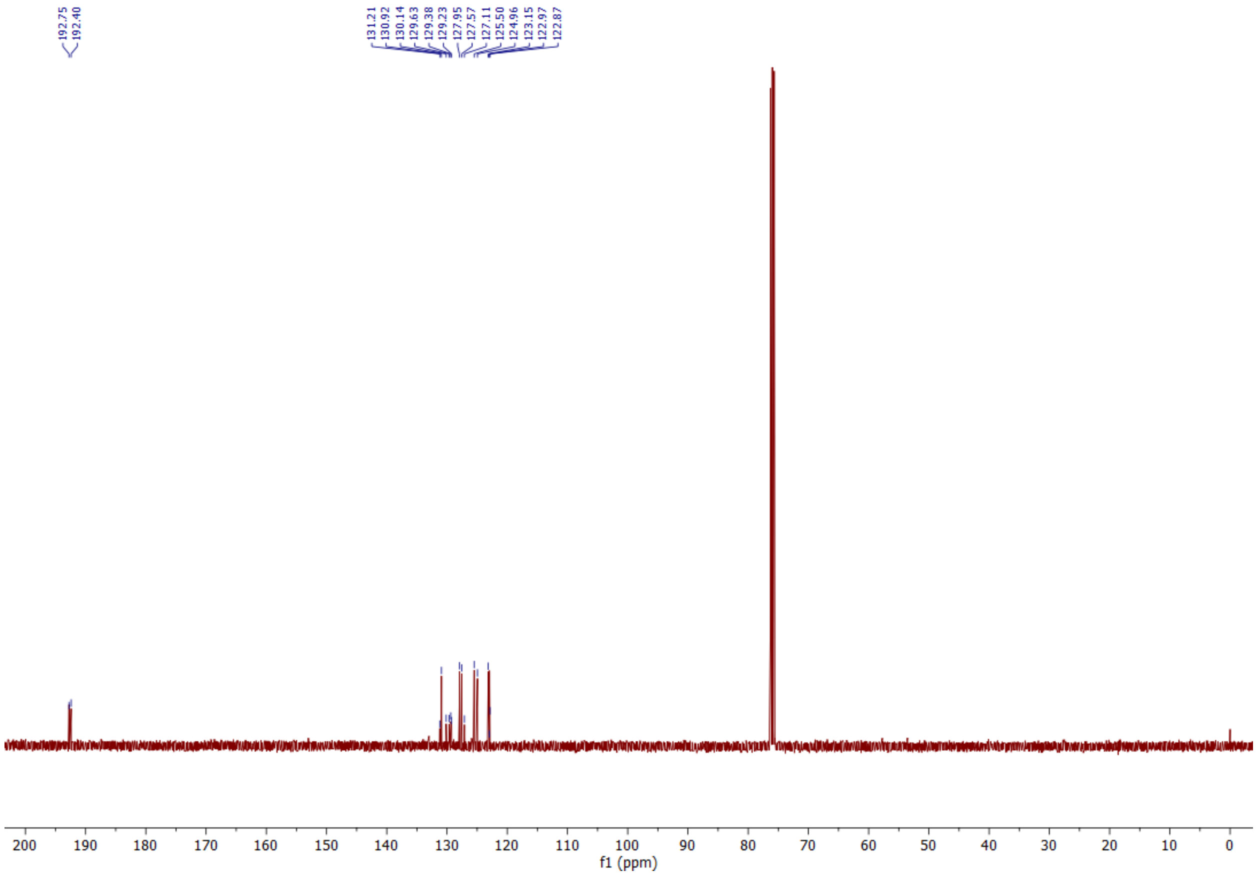

Figure S9. ^{13}C NMR spectra (101 MHz in $CDCl_3$) of A-Br-CHO.

1.7. 2-Iodoantracene (A-I)

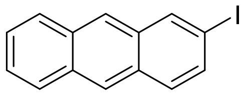

The following procedure was adapted from the previously published literature[2]. A mixture of 2-bromoanthracene (2.582 g, 10.09 mmol), KI (15.12 g, 91.09 mmol, 9.03 equiv.) and CuI (5.934 g, 31.16 mmol, 3.09 equiv.) in 38.7 ml 1,3-dimethyl-2-imidazolidinone was placed in 100 ml flask. The mixture was purged with N_2 and heated with vigorous stirring at 200 °C for 20 hours. After cooling to room temperature, brine and ice were added. The reaction vessel was placed in an ice bath for several hours, then precipitated inorganic salts were removed by filtration. The mixture underwent rotary evaporation under high vacuum to remove the excess of 1,3-dimethyl-2-imidazolidinone. The solid residue was extracted with DCM and the product was purified through

column chromatography (hexane/$CHCl_3$/ethyl acetate 5:3:1, R=0.9), providing 2-iodoanthracene (A-I) as a yellow solid (1.466 g, 4.82 mmol). Yield 47.8 %.

^{1}H NMR (400 MHz, $CDCl_3$) δ 8.36 (dt, J = 1.5, 0.8 Hz, 1H), 8.31 (s, 1H), 8.23 (s, 1H), 7.96 – 7.89 (m, 2H), 7.70 – 7.64 (m, 1H), 7.58 (dd, J = 9.0, 1.6 Hz, 1H), 7.47 – 7.38 (m, 2H).

^{13}C NMR (101 MHz, $CDCl_3$) δ 136.71, 133.49, 132.77, 131.79, 131.52, 129.76, 129.48, 128.07, 126.38, 126.04, 125.70, 125.17, 125.03, 91.03.

HRMS-EI: Measured (m/z): 303.97. Theoretical (m/z): 303.97.

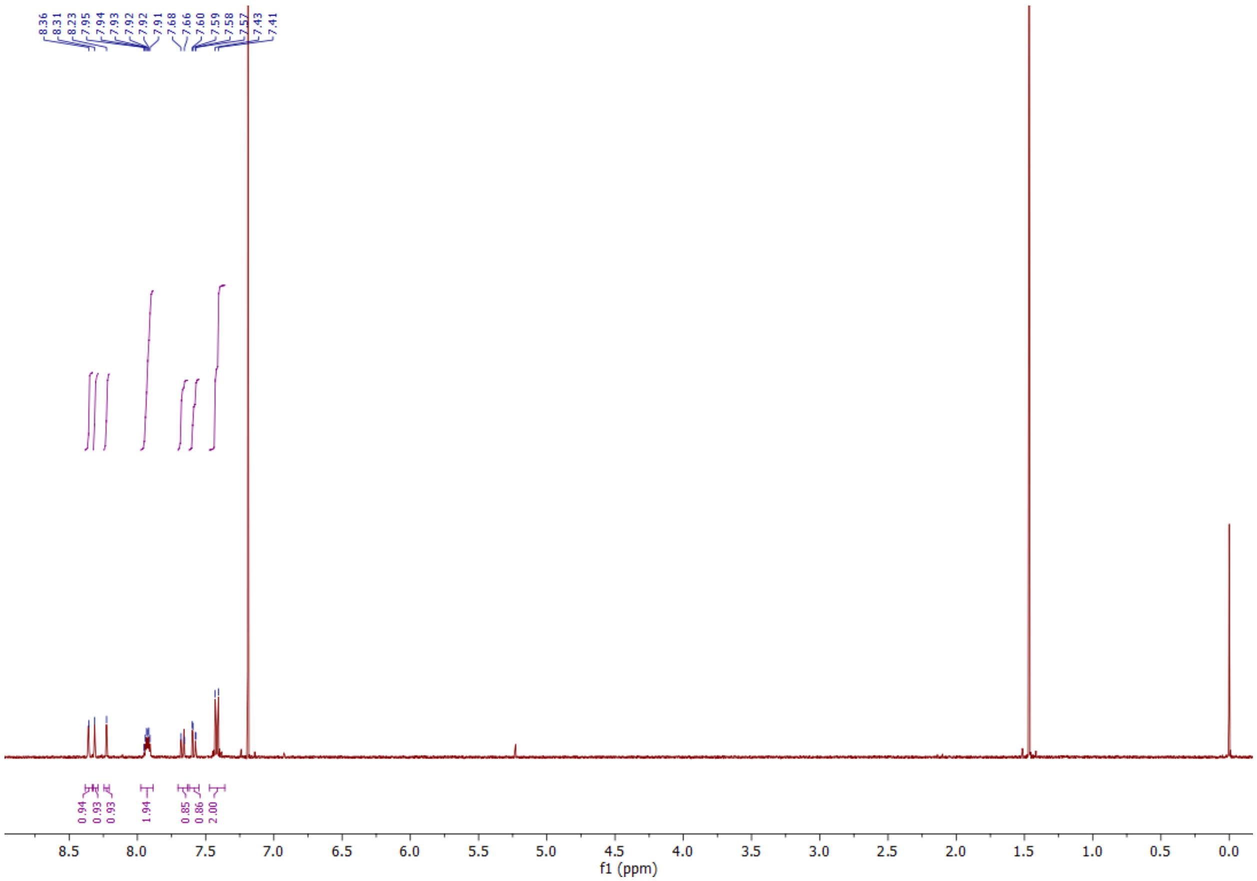

Figure S10. ^{1}H NMR spectra (400 MHz in $CDCl_3$) of A-I.

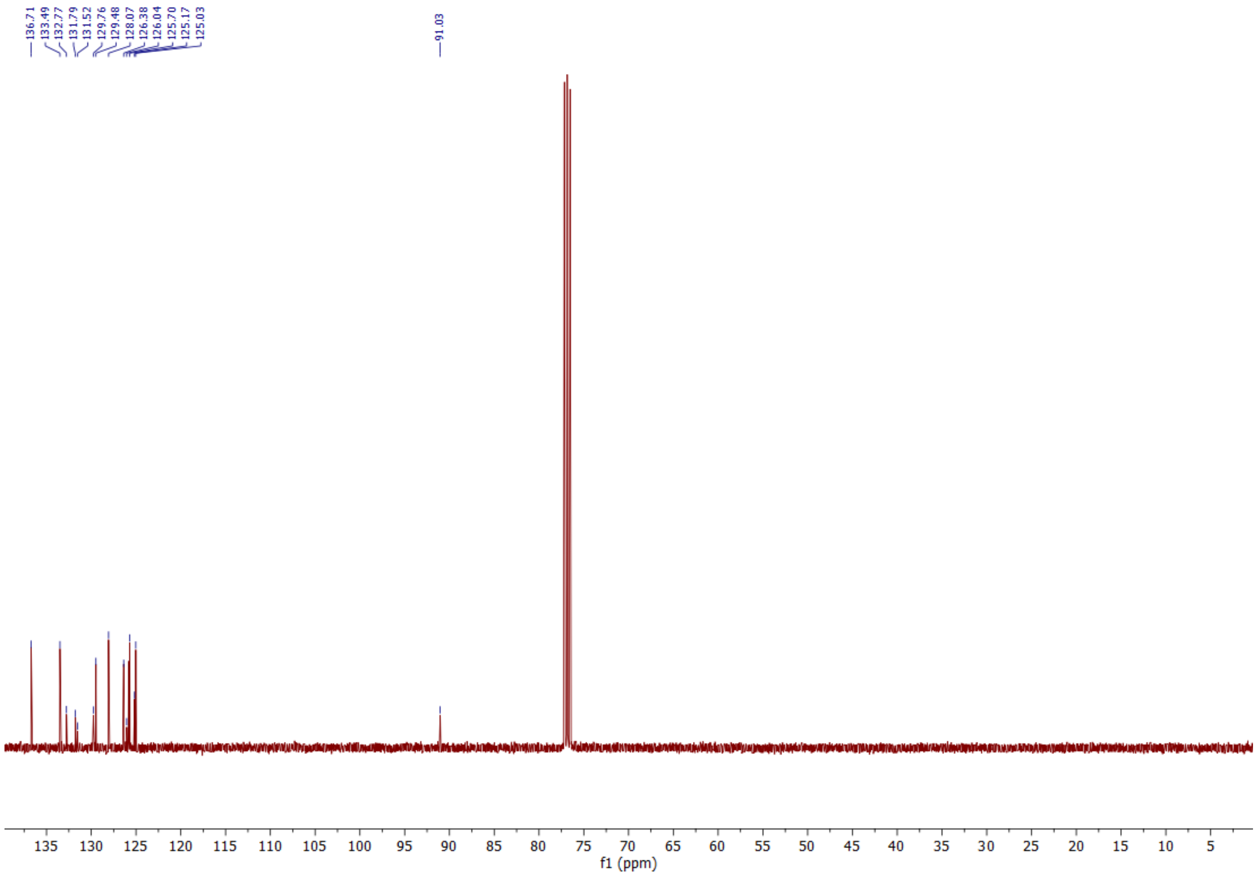

Figure S11. ^{13}C NMR spectra (101 MHz in $CDCl_3$) of A-I.

1.8. 2-Iodo-9,10-dihydro-9,10-[4,5]epidioxoloanthracen-13-one (A-I-epO)

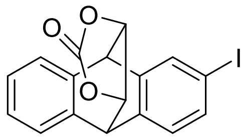

The following procedure was adapted from the previously published literature.[1] A-I (1.446 g, 4.76 mmol, 1 equiv.) and vinylene carbonate (2.99 g, 34.73 mmol, 7.3 equiv.) were heated under reflux with stirring for 18 hours, slowly forming a dark brown solution. The consumption of A-I was monitored by thin-layer chromatography (CH_2Cl_2/hexane 1:49 , R=0.30). The mixture underwent rotary evaporation under high vacuum to remove the excess vinylene carbonate, providing the 2-iodo-9,10-dihydro-9,10-[4,5]epidioxoloanthracen-13-one (A-I-epO) as a dark-brown solid (1.73 g, 92.8 %). The product was used for the further reaction without additional purification.

^{1}H NMR (400 MHz, $CDCl_3$) δ 7.64 (s, 1H), 7.54 – 7.41 (m, 1H), 7.34 – 7.23 (m, 2H), 7.16 (dd, *J* = 9.2, 4.0 Hz, 2H), 7.03 (dd, *J* = 7.7, 2.9 Hz, 1H), 4.83 – 4.70 (m, 2H), 4.56 (d, *J* = 9.4 Hz, 2H).

HRMS-EI: Measured (*m*/*z*): 389.96. Theoretical (*m*/*z*): 391.99.

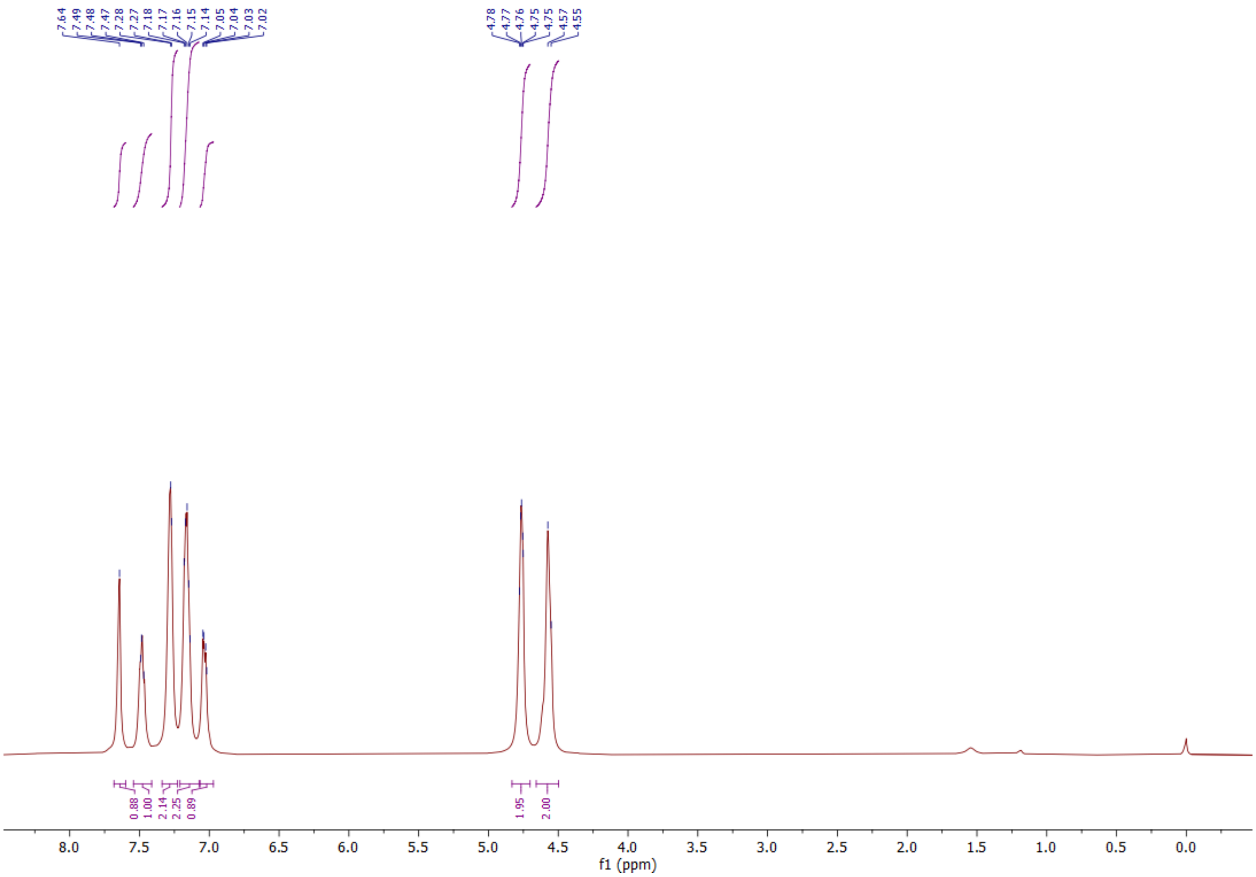

Figure S12. ^{1}H NMR spectra (400 MHz in $CDCl_3$) of A-I-epO.

1.9. 2-Iodo-9,10-dihydro-9,10-ethanoanthracene-11,12-diol (A-I-$(OH)_2$)

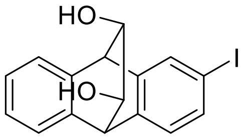

The following procedure was adapted from the previously published literature.[1] Solid potassium hydroxide (0.985 g, 17.6 mmol, 5.1 equiv.), deionized water (16.4 mL), and absolute ethanol (1.75 mL) were added to the A-I-epO (1.72 g, 4.39 mmol, 1 equiv.). The solution was stirred at 75° C for 3 hours. The consumption of the A-I-epO intermediate was monitored through thin-layer

chromatography (100% CH_2Cl_2, R=0.30). Afterwards, the solution underwent rotary evaporation under reduced pressure to remove the ethanol and roughly half of the water volume. Additional water (33 mL) was added to the solution and the solution was stirred at room temperature for one hour, resulting in the formation of light-tan solid. The contents were vacuum-filtered and then washed with deionized water. The vacuum-filtration receiving flask was changed and the solid was washed with ethyl acetate through the filter paper. The ethyl acetate was removed through rotary evaporation, leaving a yellow solid residue. The product was purified through column chromatography (CH_2Cl_2/ethyl acetate 8:1, R=0.30 & R=0.15), providing a mixture of two isomers of 2-iodo-9,10-dihydro-9,10-ethanoanthracene-11,12-diol (A-I-$(OH)_2$) as a white-yellowish solid (1.189 g, 3.25 mmol), Yield 74.0 %.

^{1}H NMR (400 MHz, $CDCl_3$) δ 7.65 (d, J = 1.7 Hz, 1H), 7.48 (dd, J = 7.8, 1.7 Hz, 1H), 7.23 (dd, J = 5.3, 3.3 Hz, 2H), 7.12 – 7.03 (m, 3H), 4.30 (dd, J = 7.3, 2.6 Hz, 2H), 4.07 – 3.96 (m, 2H).

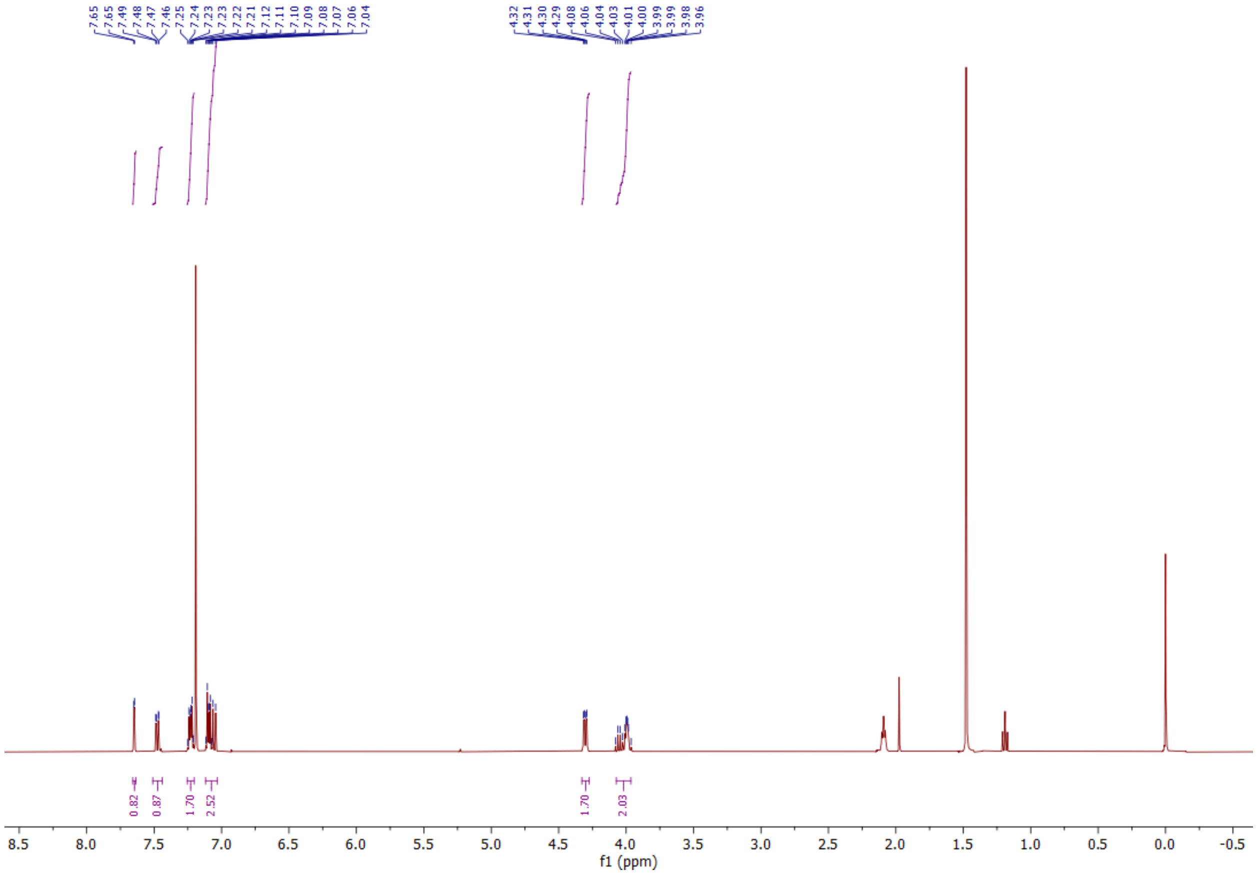

Figure S13. ^{1}H NMR spectra (400 MHz in $CDCl_3$) of A-I-$(OH)_2$.

1.10. 2-Iodoanthracene-9,10-dicarbaldehyde (A-I-CHO)

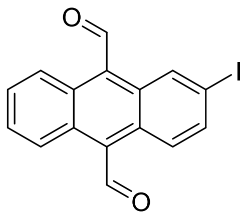

The following procedure was adapted from the previously published literature.[1] A-I-$(OH)_2$ (0.159 g, 0.43 mmol, 1 equiv.) was dissolved in anhydrous acetonitrile (10 mL) and heated to reflux with stirring, forming a transparent, light-tan solution. Lead tetraacetate (0.380 g, 0.86 mmol, 2 equiv.) was added portion-wise to the solution over a period of 5 minutes, quickly turning the solution opaque and brown in color. The solution was then stirred at reflux for 4 hours, resulting in the formation of a dark-orange precipitate within the brown solution. The consumption of the diol intermediates were monitored through thin-layer chromatography (CH_2Cl_2/ethyl acetate 8:2, R=0.50 & R=0.33). After the reaction was complete, the reaction mixture was concentrated via rotary evaporation and gave a burgundy residue. 10% Aqueous sodium carbonate (30 mL) was added to the residue, allowing the residue to be suspended in the aqueous solution. The organic contents were extracted from the aqueous layer using dichloromethane. The opaque, yellow organic layer was dried over anhydrous sodium sulfate, filtered, and concentrated to give a yellow-orange solid residue. The residue was purified by column chromatography (ethyl acetate/hexane 2:8, R=0.3), providing 2-iodo-9,10-anthracenedialdehyde (A-I-CHO) as an orange solid (0.414 g, 1.15 mmol). Yield 36.0 %.

^{1}H NMR (400 MHz, $CDCl_3$) δ 11.35 (s, 1H), 11.33 (s, 1H), 9.13 (d, J = 1.7 Hz, 1H), 8.62 (ddt, J = 10.8, 7.1, 3.3 Hz, 2H), 8.39 (d, J = 9.4 Hz, 1H), 7.82 (dd, J = 9.4, 1.7 Hz, 1H), 7.68 – 7.62 (m, 2H).

^{13}C NMR (101 MHz, $CDCl_3$) δ 192.68, 192.52, 135.82, 132.31, 131.10, 129.88, 129.80, 129.47, 129.19, 127.90, 127.67, 127.35, 124.55, 123.13, 123.09, 95.10.

HRMS-EI: Measured (m/z): 360.02. Theoretical (m/z): 359.96.

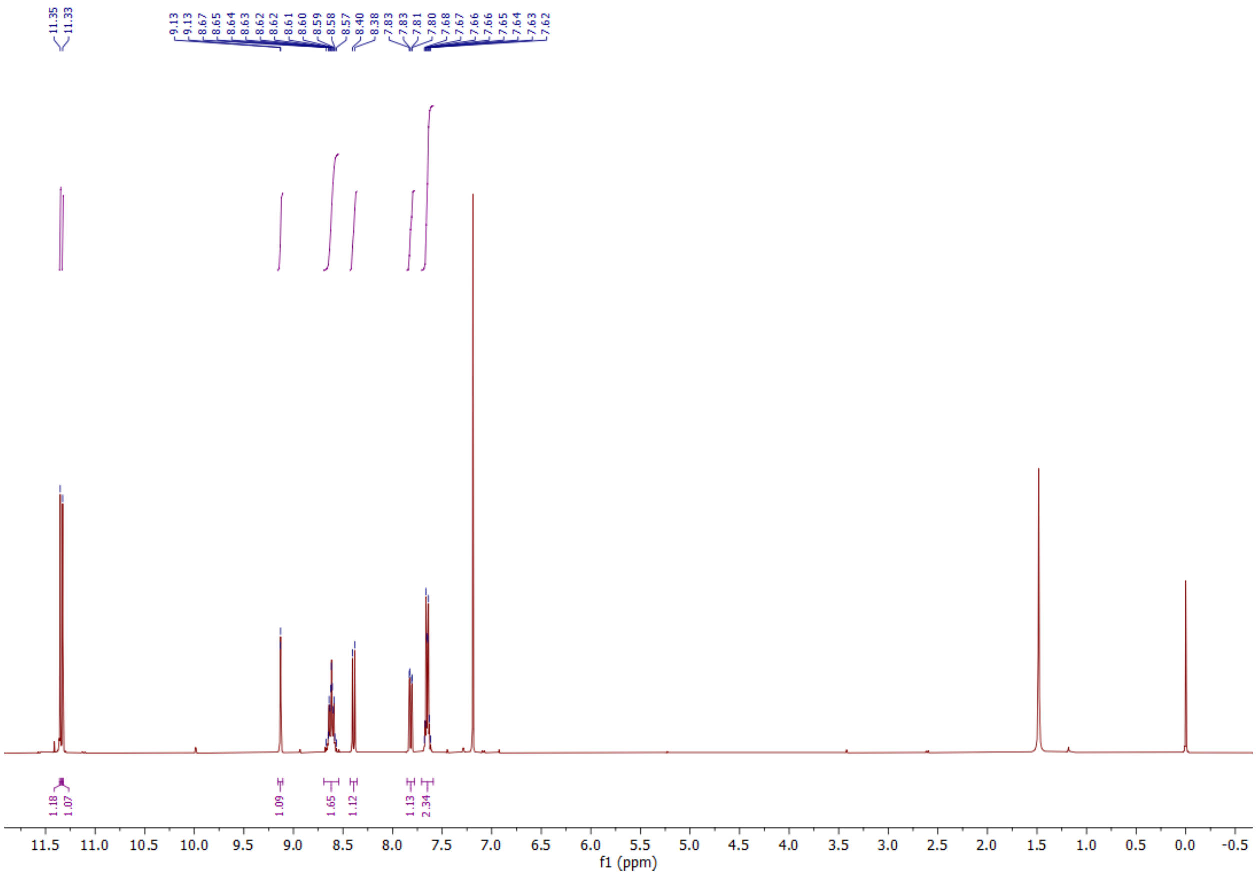

Figure S14. ^{1}H NMR spectra (400 MHz in $CDCl_3$) of A-I-CHO.

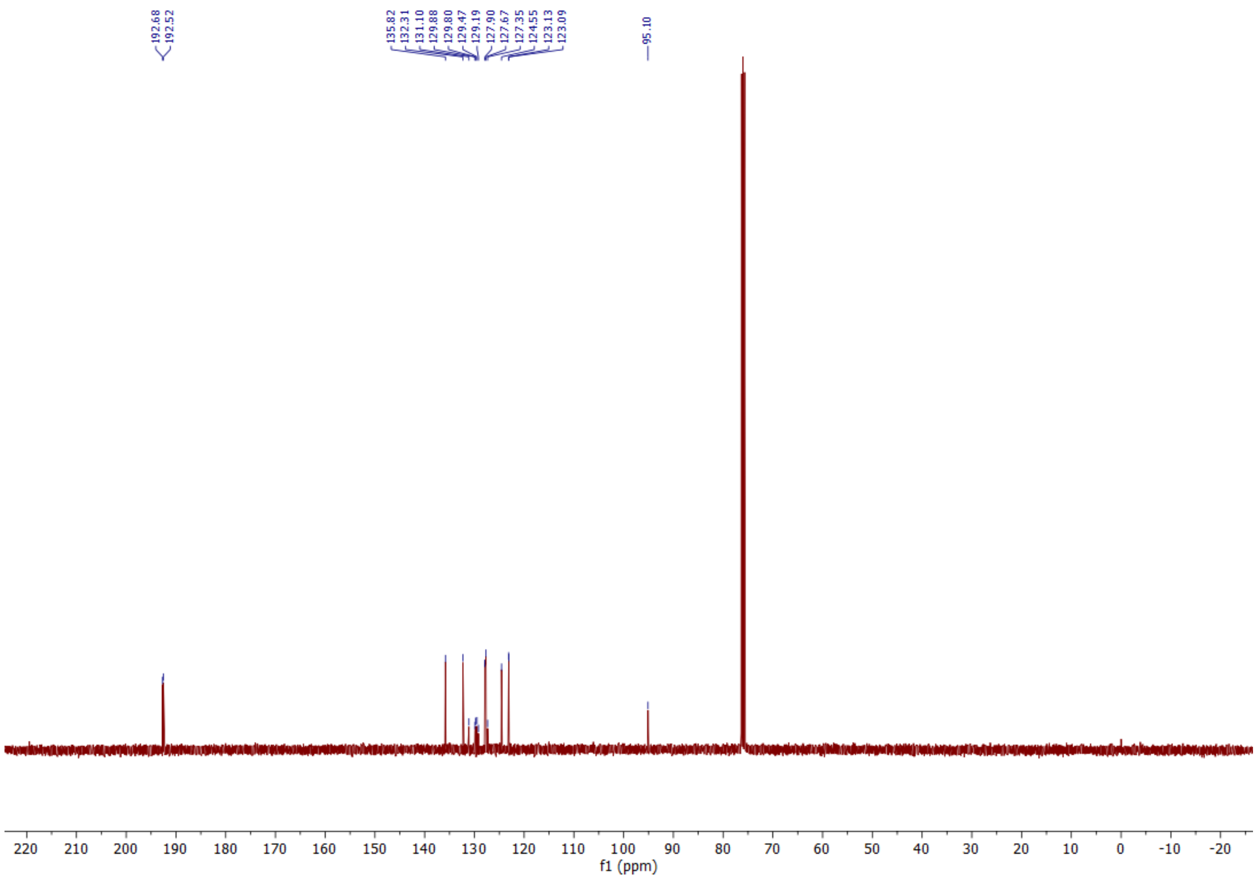

Figure S15. ^{13}C NMR spectra (101 MHz in $CDCl_3$) of A-I-CHO.

1.11. FT-IR analysis of linkers

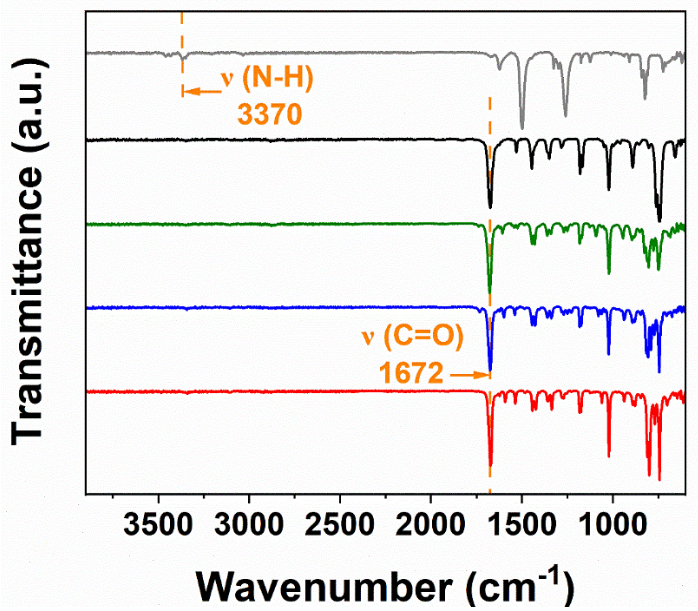

Figure S16. FT-IR spectra of N,N,N',N'-tetrakis(4-aminophenyl)-1,4-phenylenediamine (W-NH_2) (grey), anthracene-9,10-dicarbaldehyde (A-H-CHO) (black), A-Cl-CHO (green), A-Br-CHO (blue) and A-I-CHO (red). The vibration of the N–H group in the W–NH_2 moiety is visible at 3370 cm^{-1}.[3] The carbonyl stretch of the A–X–CHO linker appears at 1672 cm^{-1}.[4,5]

2. COF synthesis

2.1. W-A-H COF synthesis

W-NH_2 (9.22 mg, 19.52 µmol, 1.0 eq.) and A-H-CHO (9.14 mg, 39.05 µmol, 2.0 eq.) were filled into a 6 mL pyrex tube followed by the addition of chlorobenzene (400 µL), benzyl alcohol (BnOH) (400 µL), and 6 M acetic acid (100 µL). The tube was sealed and the reaction mixture was heated at 100 °C for 3 d. After cooling to room temperature, the resulting dark red precipitate was suction filtered, Soxhlet-extracted with dry THF, and dried under reduced pressure.

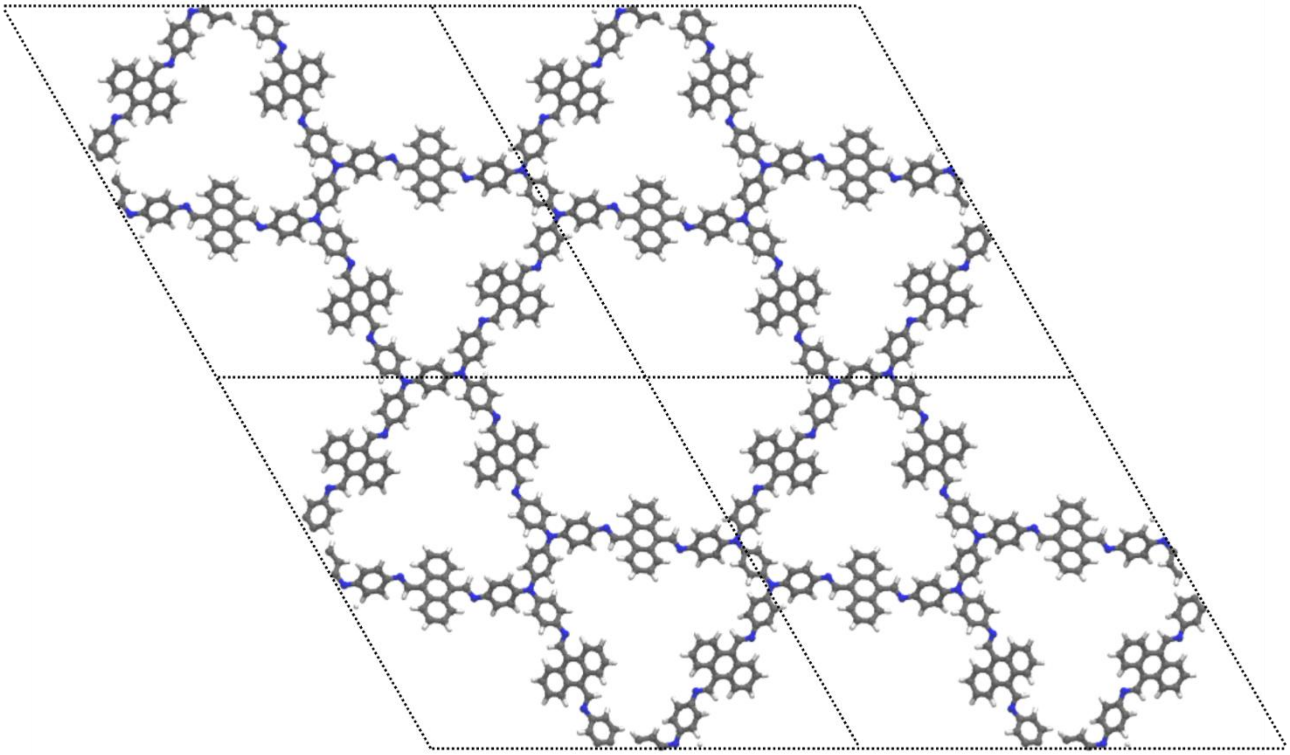

Figure S17. Simulated structure of W-A-H COF.

2.2. W-A-Cl COF synthesis

W-NH_2 (9.22 mg, 19.52 µmol, 1.0 eq.) and A-Cl-CHO (10.47 mg, 39.05 µmol, 2.0 eq.) were filled into a 6 ml pyrex tube, followed by the addition of $CHCl_3$ (400 µL), BnOH (400 µL), and 6 M acetic acid (100 µL). The tube was sealed and the reaction mixture was heated at 100 °C for 3 d. After cooling to room temperature, the resulting dark red precipitate was suction filtered, Soxhlet-extracted with dry THF, and dried under reduced pressure.

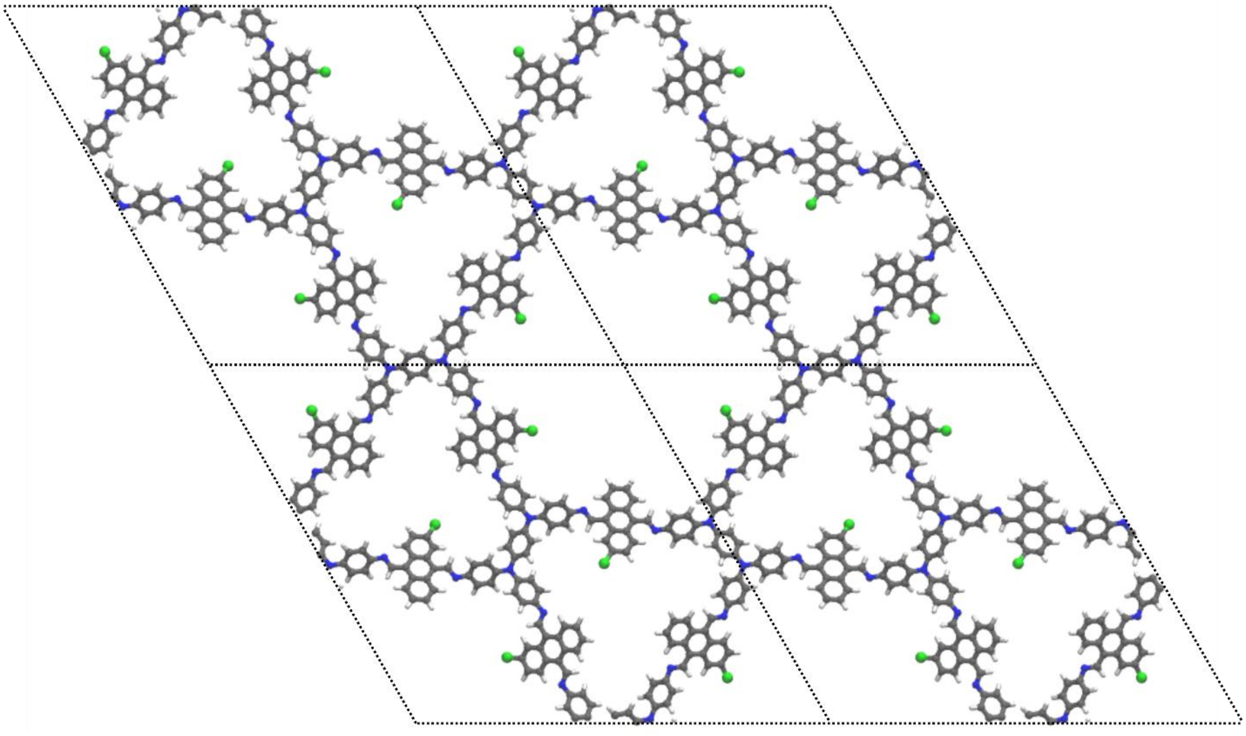

Figure S18. Simulated structure of W-A-Cl.

2.3. W-A-Br COF synthesis

W-NH_2 (9.22 mg, 19.52 μmol, 1.0 eq.) and A-Br-CHO (12.18 mg, 39.05 μmol, 2.0 eq.) were filled into a6 ml pyrex tube, followed by the addition of chlorobenzene (400 μL), BnOH (400 μL), and 6 M acetic acid (100 μL). The tube was sealed and the reaction mixture was heated at 100 °C for 3 d. After cooling to room temperature, the resulting dark red precipitate was suction filtered, Soxhlet-extracted with dry THF, and dried under reduced pressure.

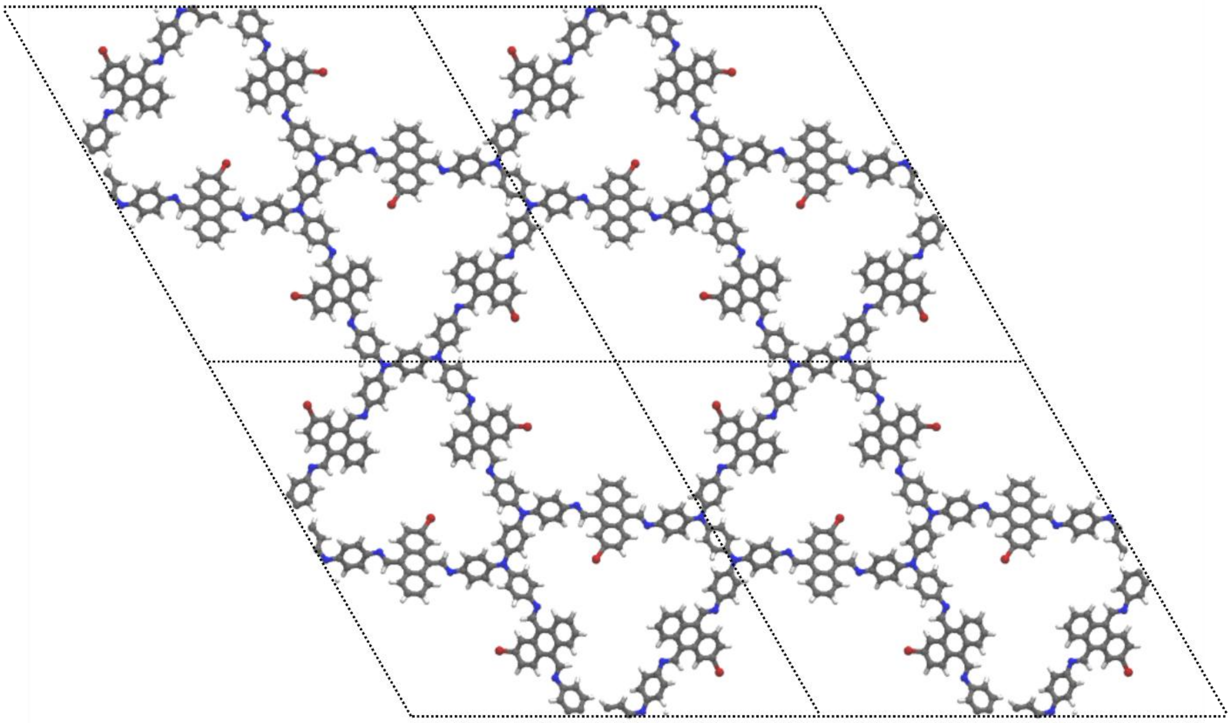

Figure S19. Simulated structure of W-A-Br COF.

2.4. W-A-I COF synthesis

W-NH_2 (9.22 mg, 19.52 μmol, 1.0 eq.) and A-I-CHO (14.40 mg, 39.05 μmol, 2.0 eq.) were filled into a 6 ml pyrex tube, followed by the addition of $CHCl_3$ (400 μL), BnOH (400 μL), and 6 M acetic acid (100 μL). The tube was sealed and the reaction mixture was heated at 100 °C for 3 d. After cooling to room temperature, the resulting dark red precipitate was suction filtered, Soxhlet-extracted with dry THF, and dried under reduced pressure.

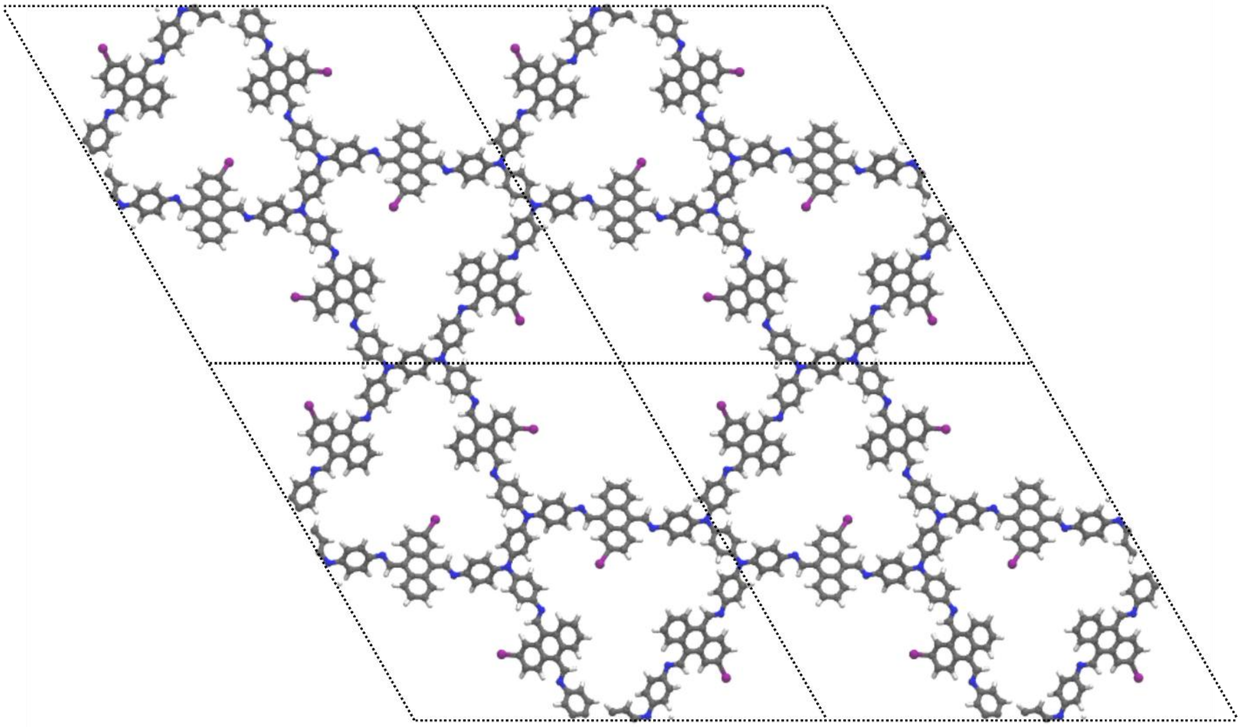

Figure S20. Simulated structure of W-A-I COF.

2.5. W-TA COF synthesis

The synthesis procedure for W-TA COF was adapted from the literature.[6] W-NH_2 (18.00 mg, 40.00 μmol, 1.0 eq.) and terephthalaldehyde (TA) (10.20 mg, 80.00 μmol, 2.0 eq.) were filled into a 6 ml pyrex tube, followed by the addition of mesitylene (1500 μL), BnOH (1500 μL), and 6 M acetic acid (150 μL). The tube was sealed and the reaction mixture was heated at 100 °C for 3 d. After cooling to room temperature, the resulting dark red precipitate was suction filtered, Soxhlet-extracted with dry THF, and dried under reduced pressure.

3. Simulations of different halogen positions

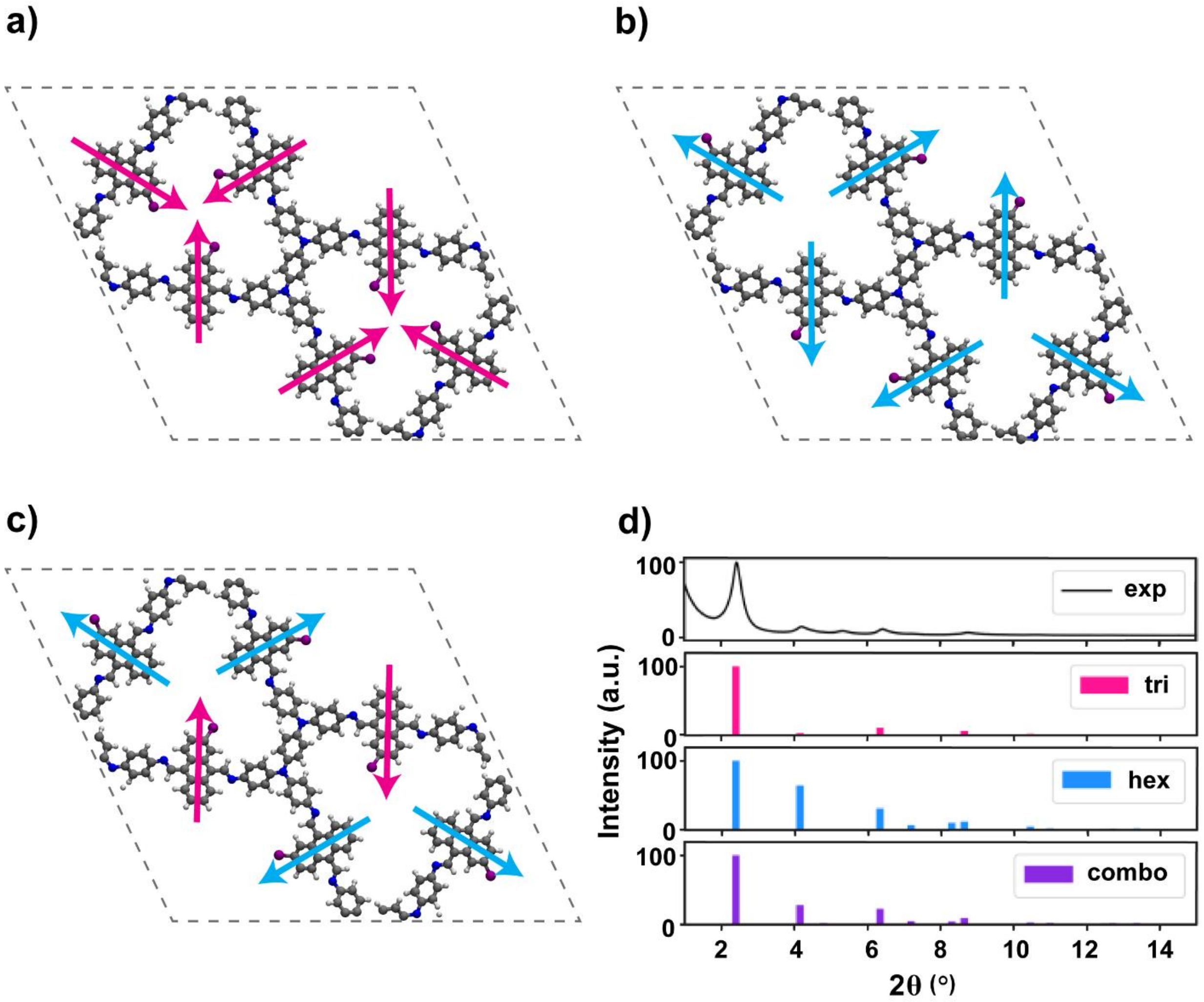

Figure S21. Simulated structures of W-A-Br COF when halogens atoms are facing (a) exclusively into the trigonal pore (pink arrows), (b) facing exclusively into the hexagonal pore (blue arrows) and (c) with a combination of two halogens facing trigonal pores and four halogens facing the hexagonal pores per unit cell. (d) Experimental PXRD pattern for W-A-Br COF (black) and simulated PXRD patterns for simulated structures of all halogens facing into the trigonal pores (pink) and into the hexagonal pores (blue), as well as the simulated PXRD pattern for the combinational structure with halogens facing both types of pores (purple).

4. HRTEM images

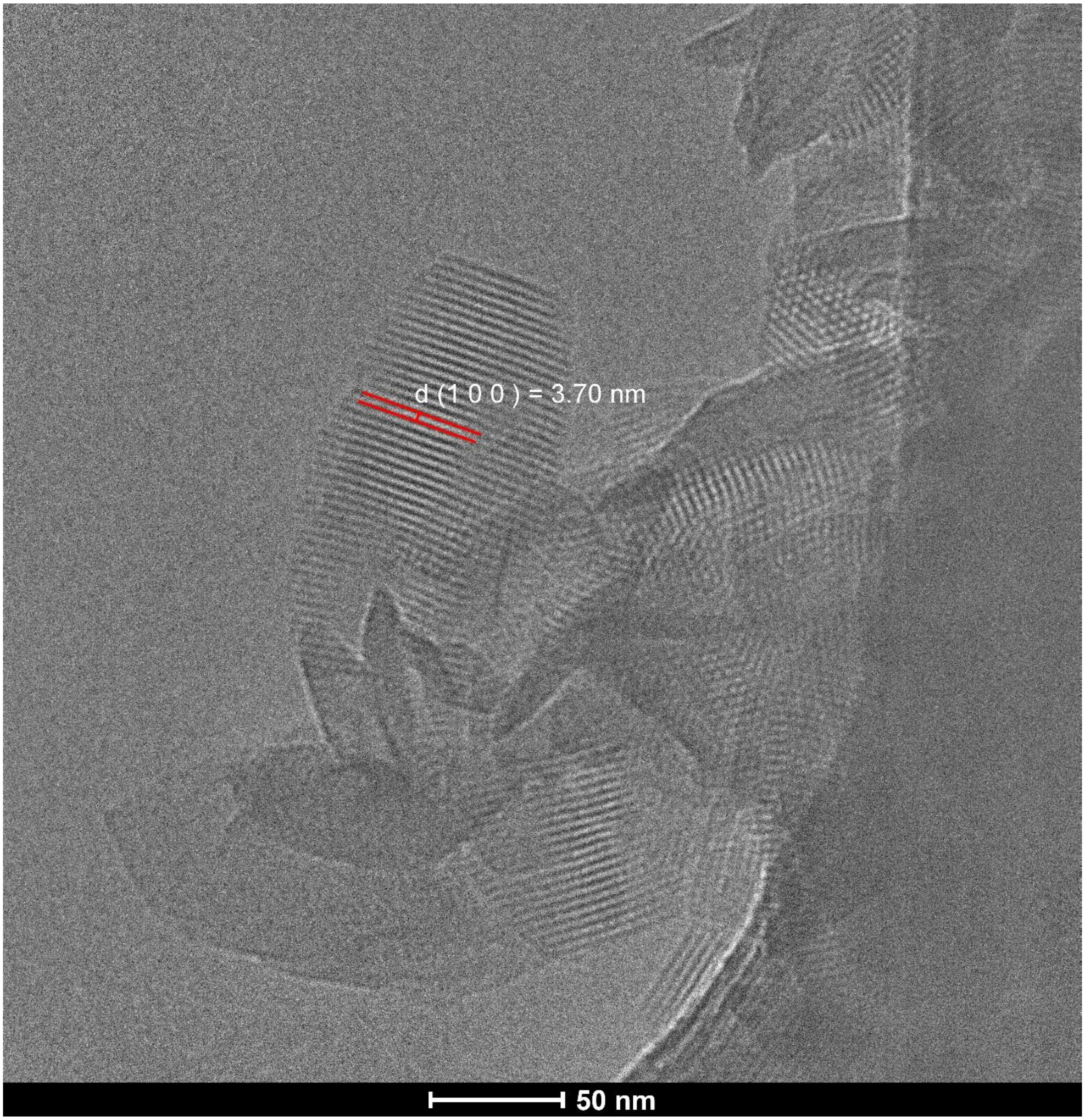

Figure S22. HRTEM image of W-A-H COF showing interlayer distance of 3.70 nm.

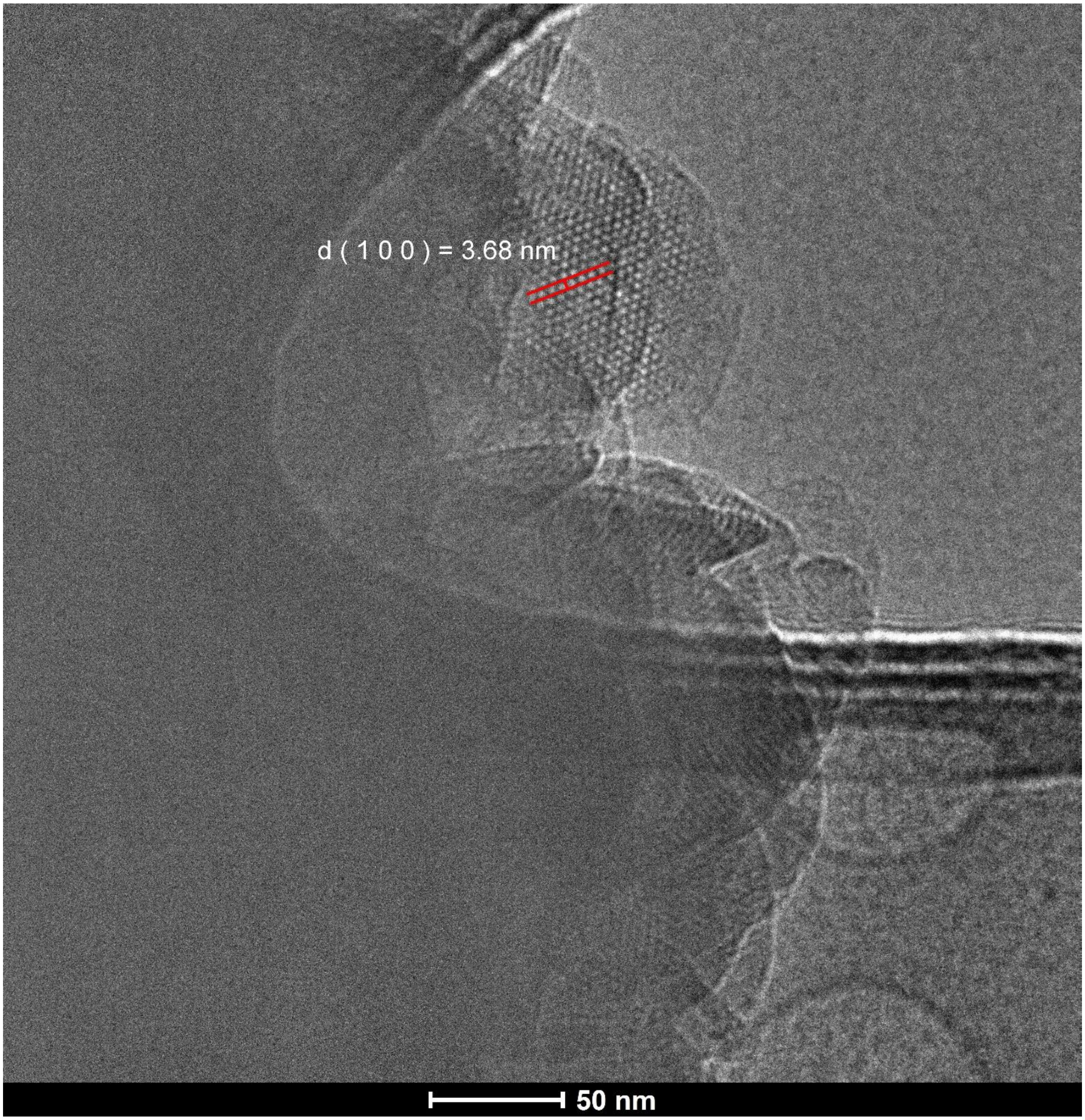

Figure S23. HRTEM image of W-A-Cl COF showing interlayer distance of 3.68 nm.

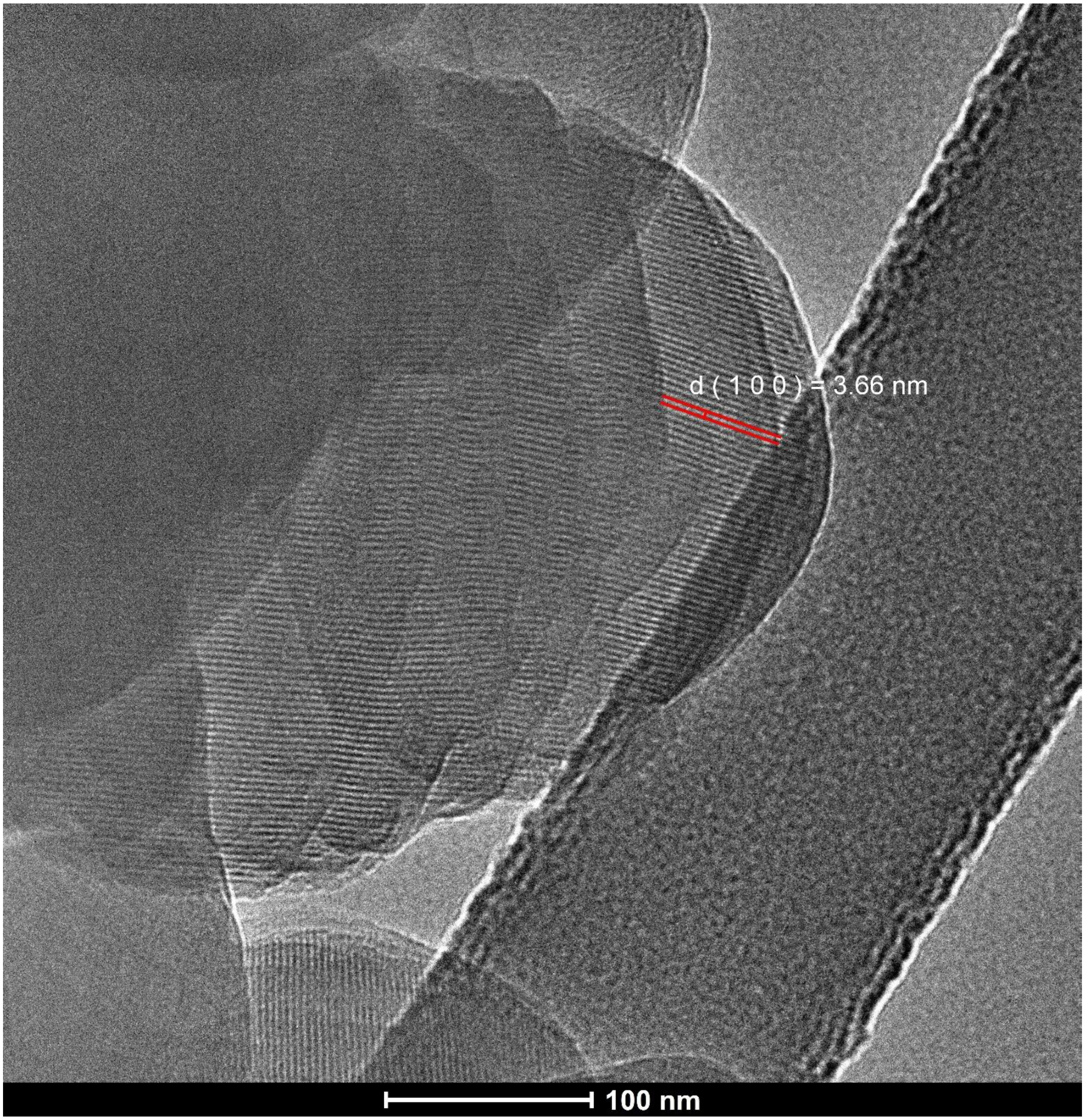

Figure S24. HRTEM image of W-A-Br COF showing interlayer distance of 3.66 nm.

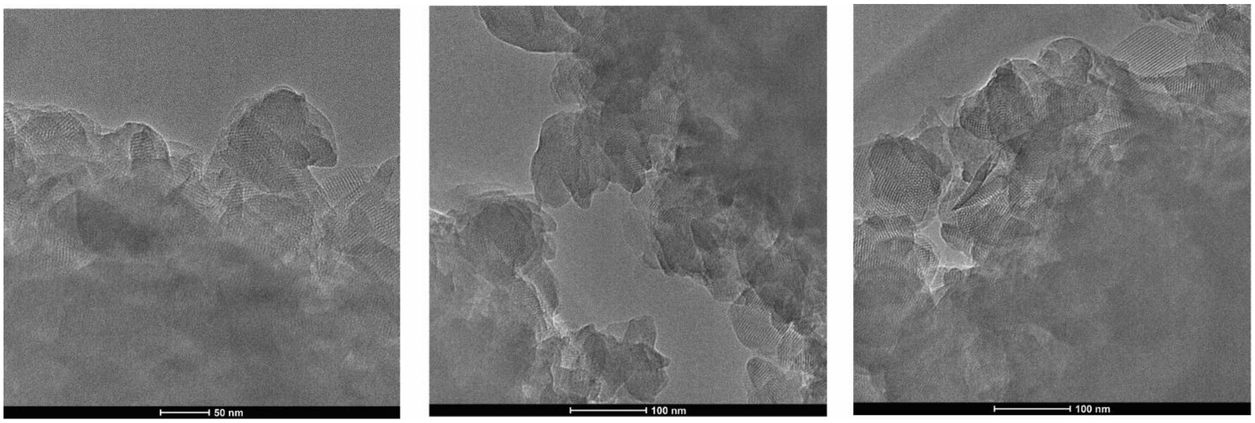

Figure S25. HRTEM images of W-A-H COF.

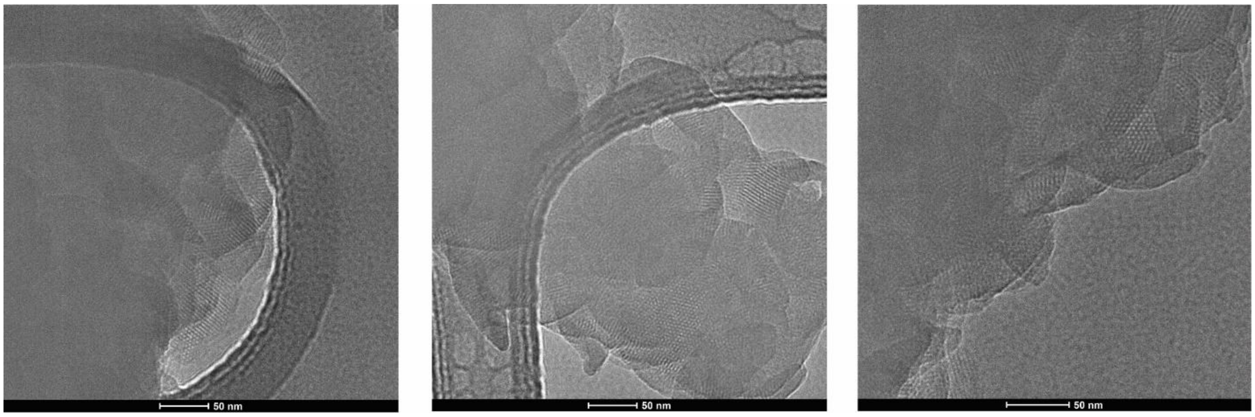

Figure S26. HRTEM images of W-A-Cl COF.

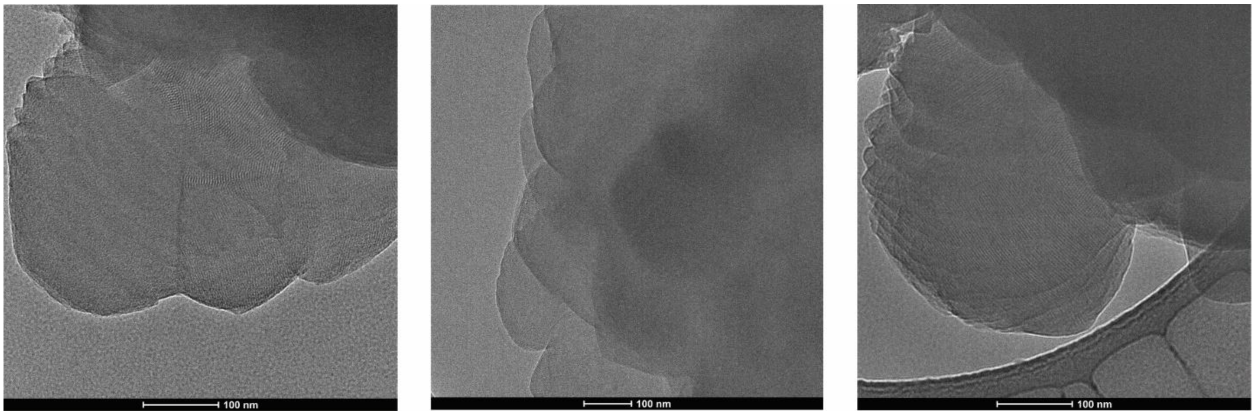

Figure S27. HRTEM images of W-A-Br COF.

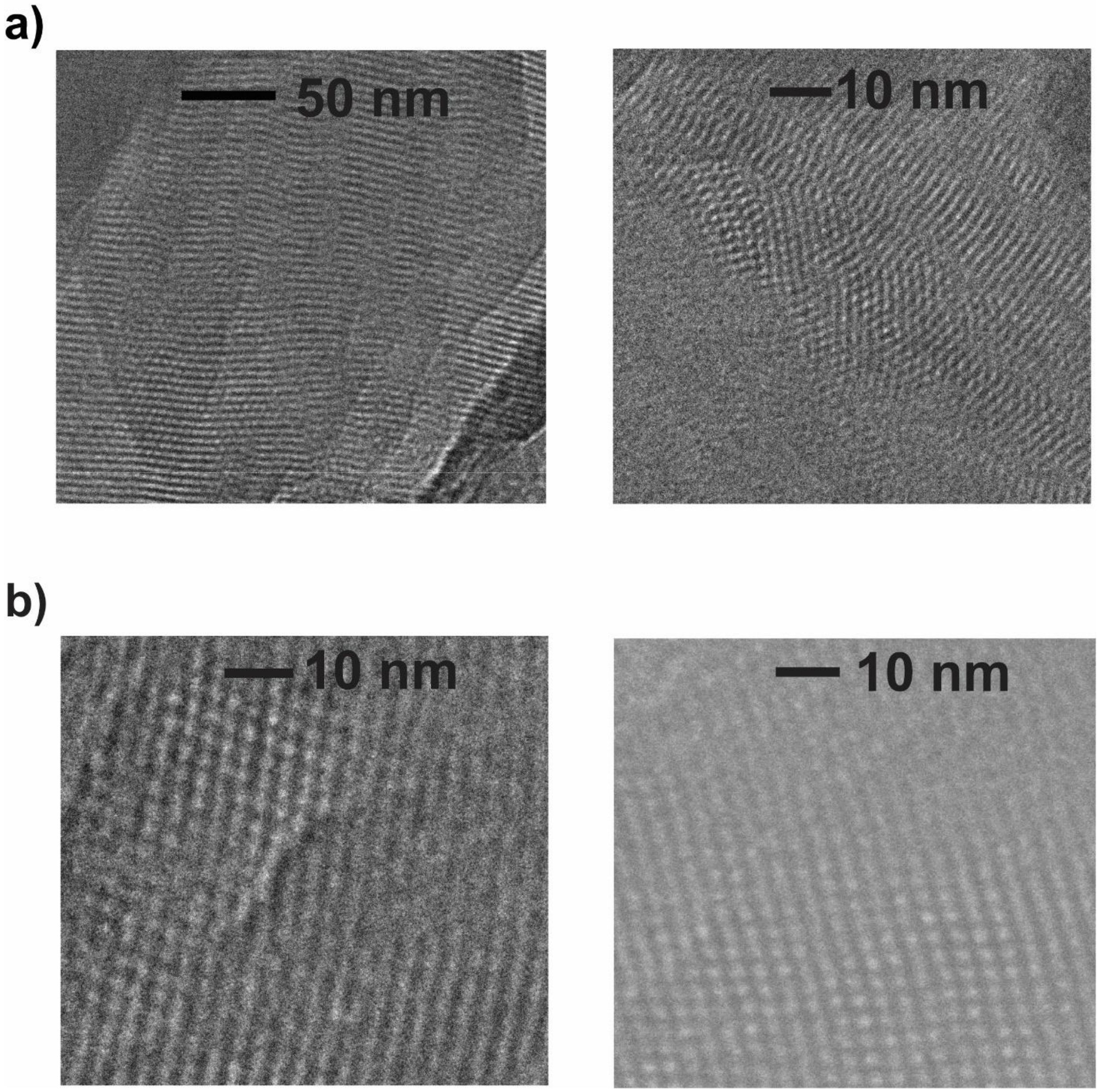

Figure S28. HRTEM images of W-A-Br COF showing (a) dominant Kagome structure with 120° angle and (b) distorted crystalline structure and revealing a lattice angles of approximately 100°.

5. ESP calculations

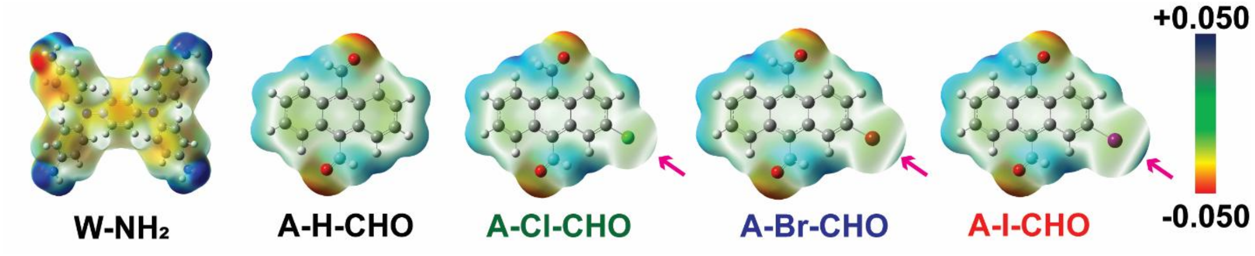

Figure S29. ESP maps superimposed on the M06-2X/def2-SVPP optimized geometry of W-NH_2 and anthracene-based linkers A-X-CHO (X = H, Cl, Br, I). The ESP values in atomic units (a.u.) are mapped onto the total electron density surface with an isovalue of 0.001 highlighting electron-rich (red) and electron-deficient (blue) regions. Introduction of Cl, Br, and I induces progressively larger σ-holes (positive ESP regions) opposite the C-X bonds (highlighted with pink arrows).

6. SEM images

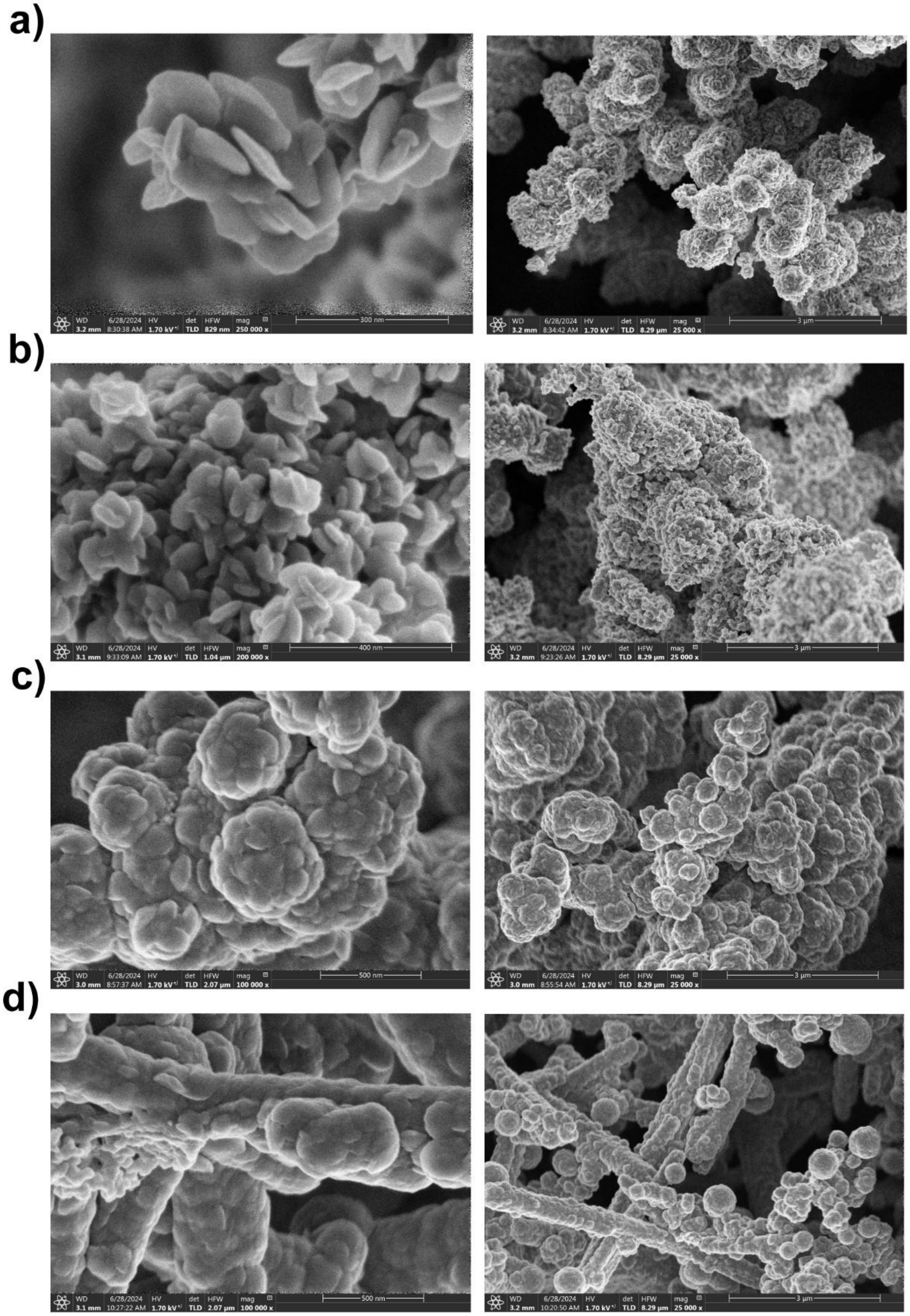

Figure S30. SEM images of (a) W-A-H, (b) W-A-Cl, (c) W-A-Br, (d) W-A-I.

7. Sorption and porosity parameters

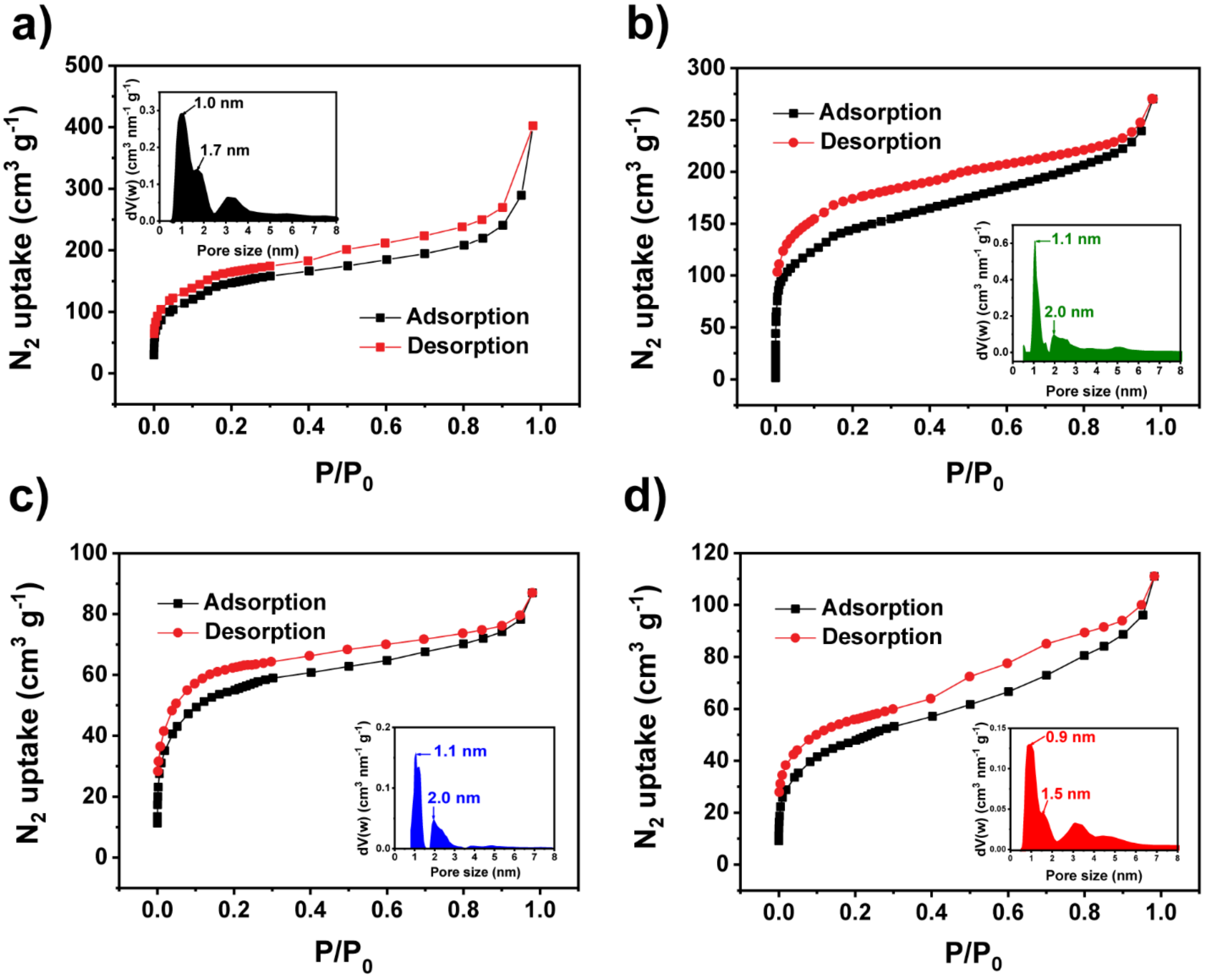

Figure S31. Nitrogen sorption isotherms and pore size distributions (insets) of the COFs (a) W-A-H, (b) W-A-Cl, (c) W-A-Br, (d) W-A-I.

Table S1. Theoretical (*Zeo++[7] and **PoreBlazer v4.0[8]) and experimental geometric porosity parameters of the COFs (probe radius corresponds to N_2; 0.18 nm). *** Experimental pore volume was calculated based on the average uptake from the desorption branch in the relative pressure range P/P_0 = 0.60-0.80.

COF	Pore	*Maximum pore diameter (m_{pd}) (nm)	*Pore window size (p_{ws}) (nm)	**Theoretical pore volume (V_{pt}) ($cm^3 g^{-1}$)	Experimental pore volume*** (V_{et}) ($cm^3 g^{-1}$)	*Unit cell density ($g cm^{-3}$)	Experimental BET surface area ($m^2 g^{-1}$)	Theoretical network-accessible surface area ($m^2 g^{-1}$)

W-A-H	Trigonal	0.73	0.70	0.364	0.35	0.67	548	897
	Hexagonal	2.08	2.07					
W-A-Cl	Trigonal	0.68	0.62	0.317	0.33	0.74	490	806
	Hexagonal	1.88	1.86					
W-A-Br	Trigonal	0.69	0.63	0.293	0.11	0.81	187	674
	Hexagonal	1.87	1.84					
W-A-I	Trigonal	0.66	0.61	0.260	0.13	0.90	170	653
	Hexagonal	1.84	1.80					

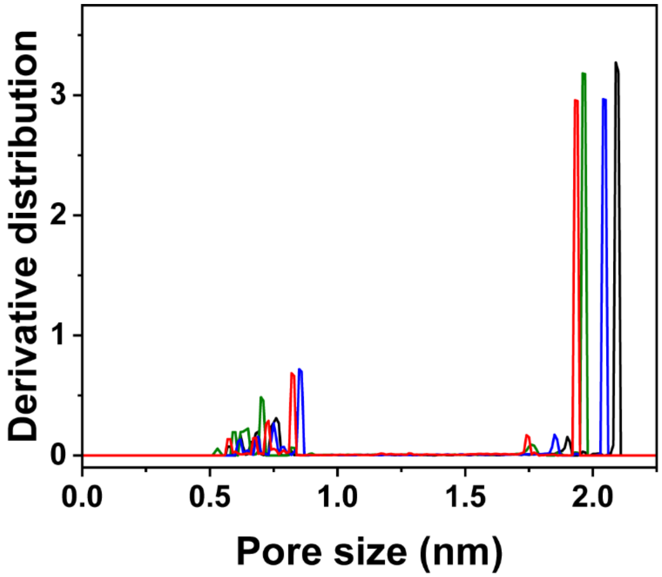

Figure S32. Simulated pore size distribution of the COFs W-A-H (black), W-A-Cl (green), W-A-Br (blue) and W-A-I (red) using Zeo++.[7]

8. FT-IR analysis of COFs

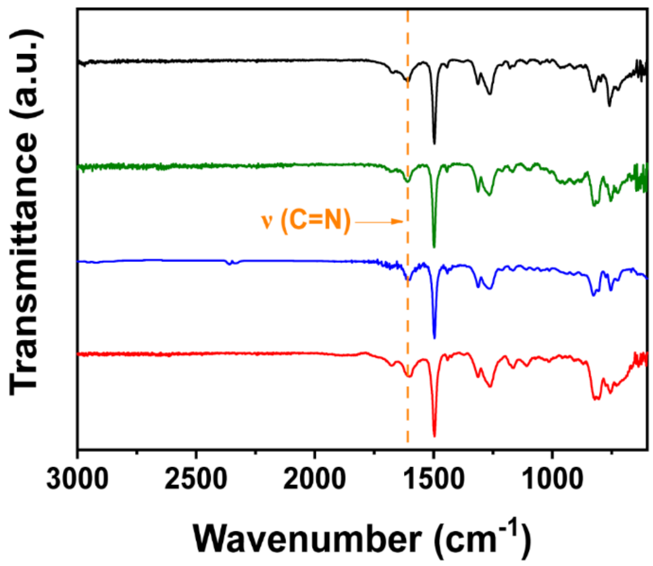

Figure S33. FT-IR spectra of the COFs W-A-H (black), W-A-Cl (green), W-A-Br (blue) and W-A-I (red).

9. Solid-state ^{13}C NMR of COFs

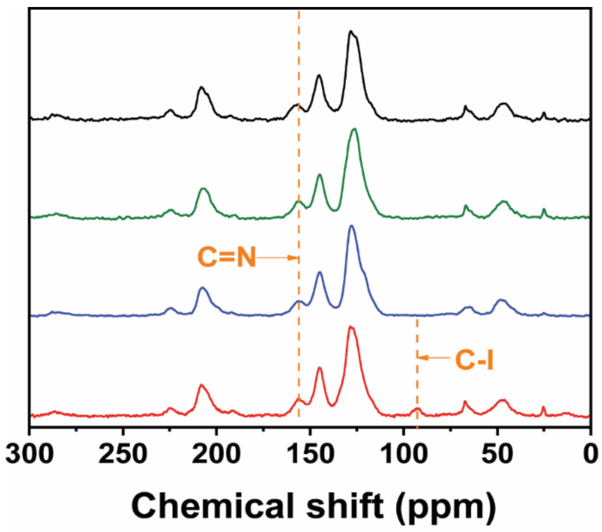

Figure S34. ^{13}C NMR spectra of the COFs W-A-H (black), W-A-Cl (green), W-A-Br (blue) and W-A-I (red).

10. TGA analysis

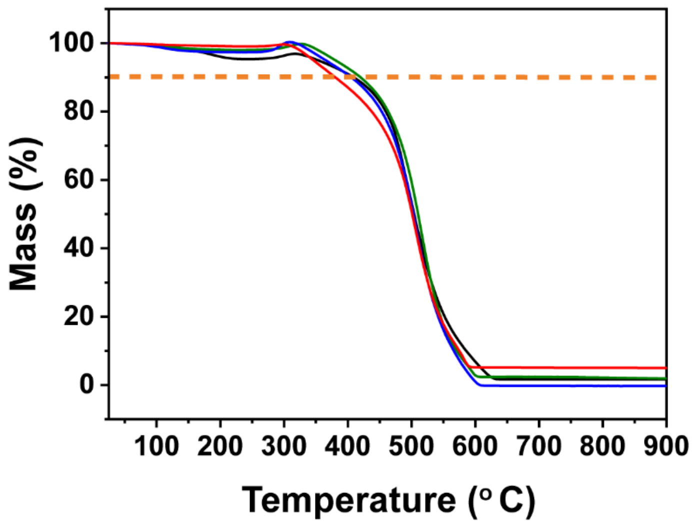

Figure S35. TGA analysis of the COFs W-A-H (black), W-A-Cl (green), W-A-Br (blue) and W-A-I (red), with decomposition temperatures determined at 10% mass loss.

11. Experimental optical properties

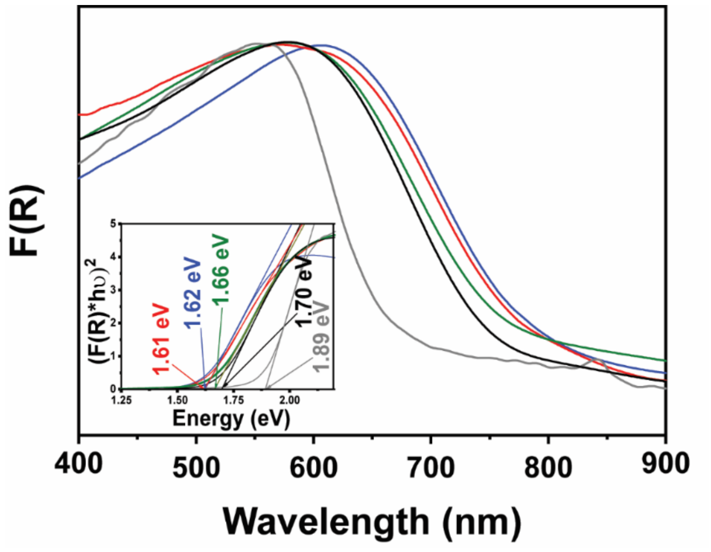

Figure S36. Optical absorption spectra (Kubelka Munk function F(R)) and Tauc plots of the COFs W-TA (grey), W-A-H (black), W-A-Cl (green), W-A-Br (blue), W-A-I (red).

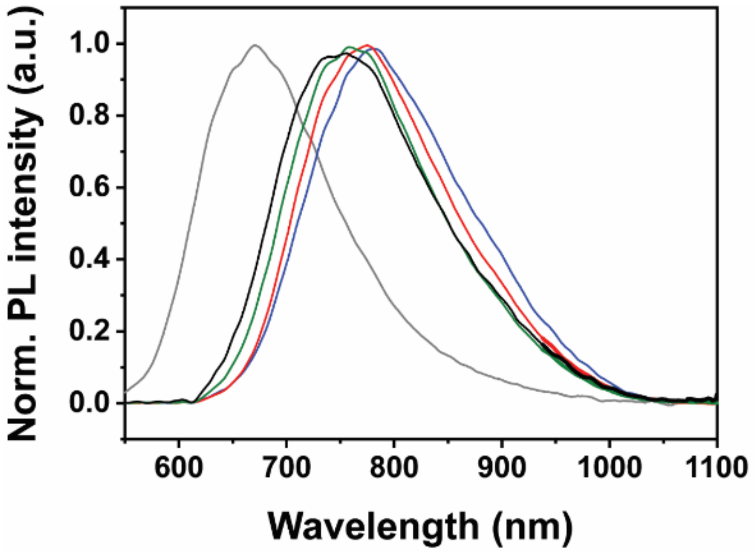

Figure S37. PL of the COFs W-TA (grey), W-A-H (black), W-A-Cl (green), W-A-Br (blue), W-A-I (red).

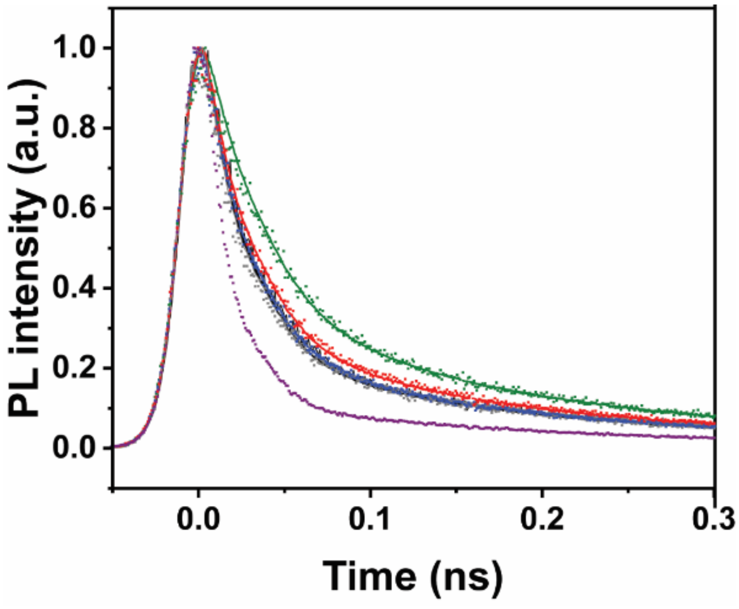

Figure S38. Time-resolved PL lifetime measurements of the COFs W-TA (grey), W-A-H (black), W-A-Cl (green), W-A-Br (blue), W-A-I (red). The purple curve represents the instrument response function (IRF).

12. Calculated optical properties

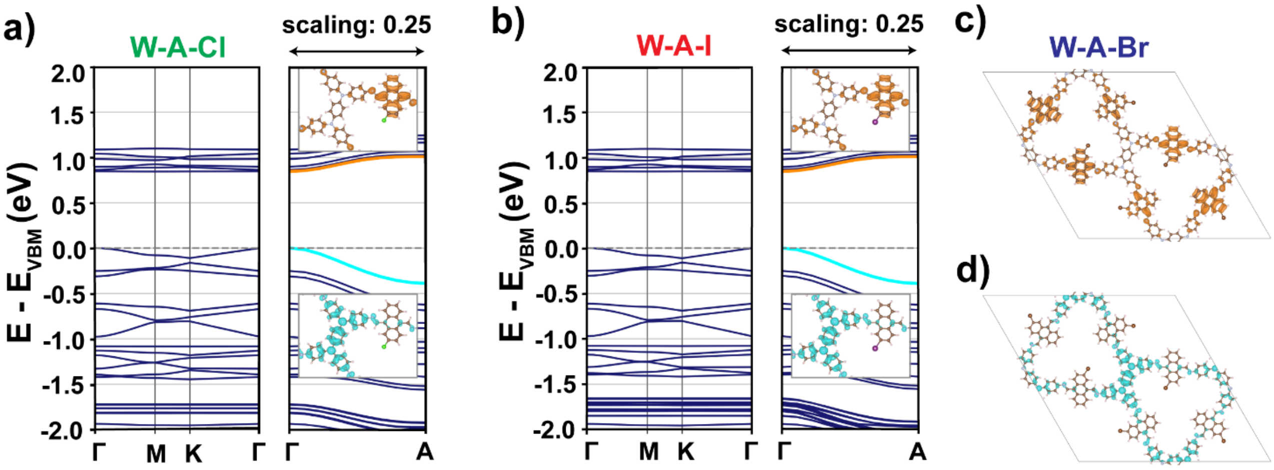

Figure S39. Electronic band structures of (a) W-A-Cl and (b) W-A-I COFs with insets of the partial charge densities of LUMO (orange) and HOMO (blue) bands. Partial charge densities of (c) LUMO band and (d) HOMO band of W-A-Br.

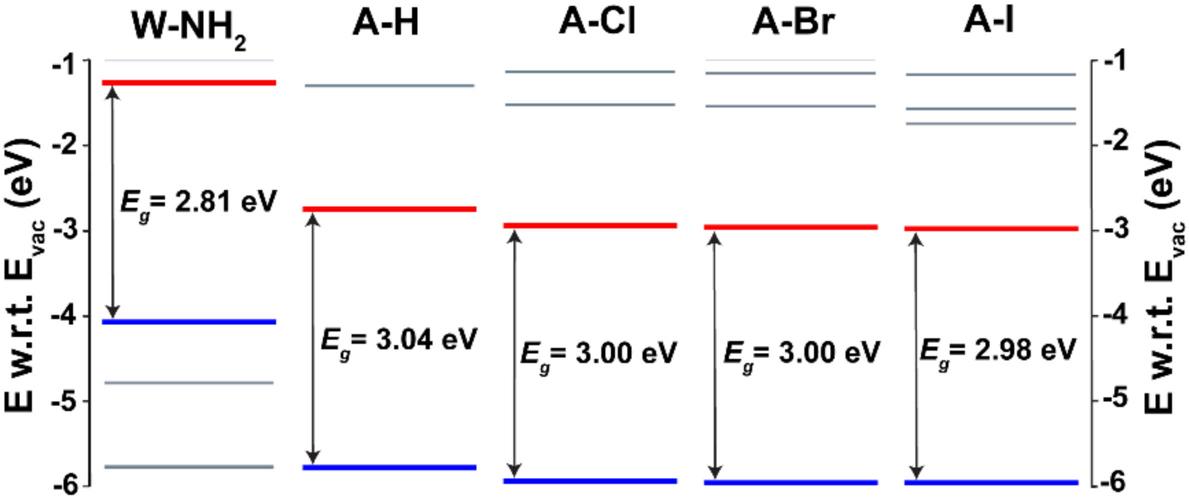

Figure S40. Kohn-Sham eigenvalues of $W-NH_2$, and (non-)halogenated anthracene (A-X, X = H, Cl, Br, I) building blocks with highlighted HOMO (blue) and LUMO (red).

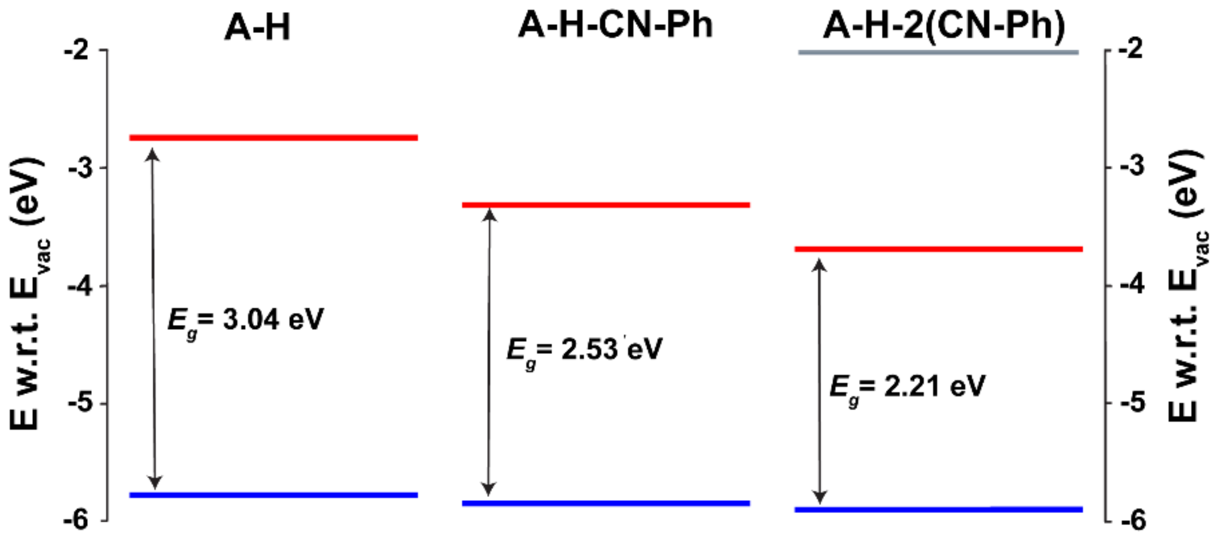

Figure S41. Kohn-Sham eigenvalues of the systematic extension of anthracene (A-H) toward a combined anthracene-Wurster fragment of W-A-H (A-H-2(CN-Ph)).

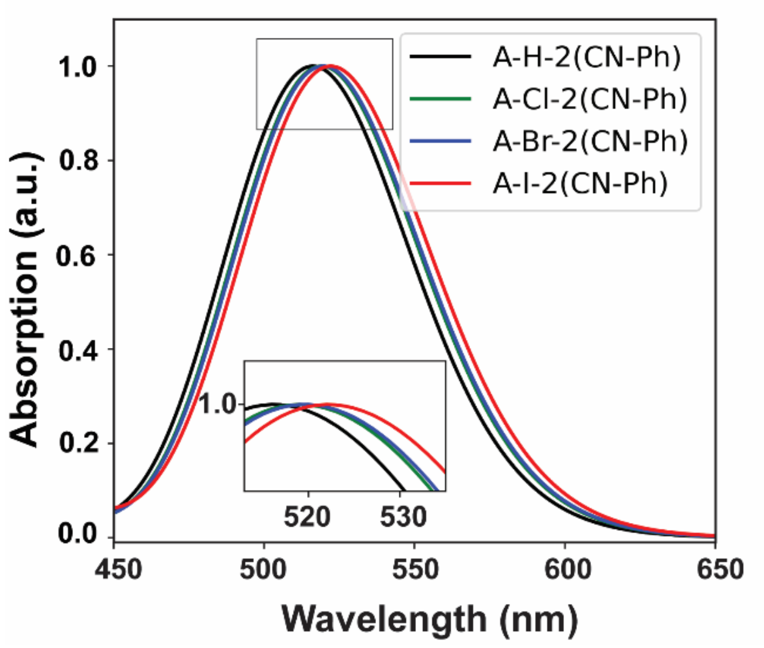

Figure S42. Theoretical absorption spectrum of (non-)halogenated extensions of anthracene towards a combined Wurster-anthracene fragment; inset shows close-up of the absorption maxima.

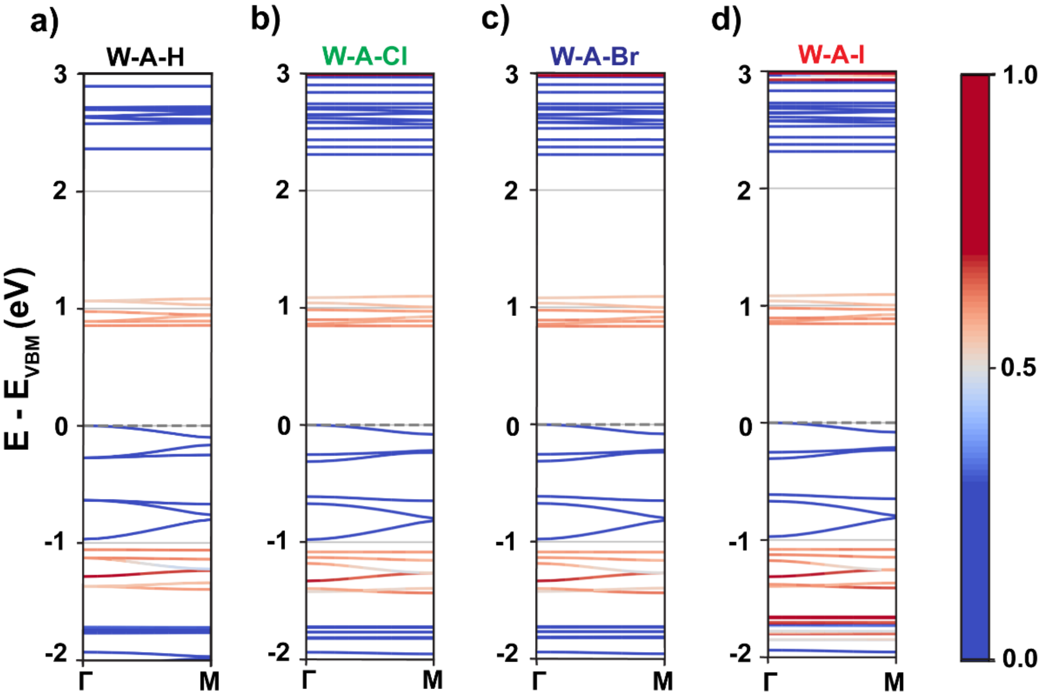

Figure S43. Projected band structure of (a) W-A-H, (b) W-A-Cl, (c) W-A-Br, and (d) W-A-I orbitals located on the (halogenated) anthracene atoms. Dark red color indicates a band with exclusive contribution from (halogenated) anthracene, whereas dark blue corresponds to a band formed by Wurster orbitals.

13. References

(1) Otto Phanstiel. Fluorescent Cytotoxic Compounds Specific for the Cellular Polyamine Transport System, United States Patent : 5861366, **2013**.

(2) Yamashita, K. I.; Tsuboi, M.; Asano, M. S.; Sugiura, K. I. Facile Aromatic Finkelstein Iodination (AFI) Reaction in 1,3-Dimethyl-2-Imidazolidinone (DMI). *Synth. Commun.* **2012**, *42* (2), 170–175. https://doi.org/10.1080/00397911.2010.523152.

(3) Kawamata, K.; Chowdhury, P. K.; Ito, F.; Sugawara, K.; Nakanaga, T. Investigation of the N−H Stretching Vibrations of the Aniline−Pyrrole Binary Complex and Its Cation by Infrared Depletion Spectroscopy. *J. Phys. Chem. A* **1998**, *102* (25), 4788–4793. https://doi.org/10.1021/jp9811373.

(4) Jebasingh Kores, J.; Antony Danish, I.; Sasitha, T.; Gershom Stuart, J.; Jimla Pushpam, E.; Winfred Jebaraj, J. Spectral, NBO, NLO, NCI, Aromaticity and Charge Transfer Analyses of Anthracene-9,10-Dicarboxaldehyde by DFT. *Heliyon* **2021**, *7* (11), e08377. https://doi.org/10.1016/j.heliyon.2021.e08377.

(5) Bree, A.; Kydd, R. A. Infrared Spectrum of Anthracene- d 10. *J. Chem. Phys.* **1969**, *51* (3), 989–995. https://doi.org/10.1063/1.1672168.

(6) Rotter, J. M.; Guntermann, R.; Auth, M.; Mähringer, A.; Sperlich, A.; Dyakonov, V.; Medina, D. D.; Bein, T. Highly Conducting Wurster-Type Twisted Covalent Organic Frameworks. *Chem. Sci.* **2020**, *11* (47), 12843–12853. https://doi.org/10.1039/D0SC03909H.

(7) Willems, T. F.; Rycroft, C. H.; Kazi, M.; Meza, J. C.; Haranczyk, M. Algorithms and Tools for High-Throughput Geometry-Based Analysis of Crystalline Porous Materials. *Microporous Mesoporous Mater.* **2012**, *149* (1), 134–141. https://doi.org/10.1016/j.micromeso.2011.08.020.

(8) Sarkisov, L.; Bueno-Perez, R.; Sutharson, M.; Fairen-Jimenez, D. Materials Informatics with PoreBlazer v4.0 and the CSD MOF Database. *Chem. Mater.* **2020**, *32* (23), 9849–9867. https://doi.org/10.1021/acs.chemmater.0c03575.
